# Integrative Systems Genomics and Pharmacology Define Shared Immune–Vascular Programs and Prioritize an Experimentally Testable Candidate Linking Sleep Loss to Human Atherosclerosis

**DOI:** 10.3390/biomedicines14091947

**Published:** 2026-08-29

**Authors:** Lutfi Cagatay Onar, Ibrahim Yilmaz

**Affiliations:** 1 Department of Cardiovascular Surgery, Dr. Ismail Fehmi Cumalioglu City Hospital, Ministry of Health, Republic of Turkey, Tekirdag 59020, Turkey; 2 Unit of Pharmacovigilance, Dr. Ismail Fehmi Cumalioglu City Hospital, Ministry of Health, Republic of Turkey, Tekirdag 59020, Turkey

**Keywords:** sleep loss, atherosclerosis, transcriptional programs, directional concordance, RNA splicing, *SF3A3*, Mendelian randomization, participant-grouped cross-validation

## Abstract

**Background:** Sleep loss is epidemiologically linked to atherosclerotic cardiovascular disease, yet shared-transcriptome studies typically intersect differentially expressed gene lists and treat the overlap as a shared response, assuming rather than testing directional agreement. **Methods:** Controlled human sleep restriction (GSE39445; 438 whole-blood samples from 26 participants) and paired carotid plaque versus patient-matched intact artery (GSE43292; 32 patients) were compared across biological compartments at three resolutions: individual genes, annotated gene sets, and redundancy-collapsed programs. The resulting architecture was transferred unmodified to acute total sleep deprivation, plaque progression, and aneurysm enlargement, and was evaluated by participant-grouped cross-validation, network topology, exact-cis Mendelian randomization with colocalization, and single-cell localization. **Results:** Convergence was resolution dependent. The cohorts shared more differentially expressed genes than expected under independence (274 of 16,869; *p* = 3.54 × 10^−4^), yet only 48.9% changed concordantly (*p* = 0.66). Concordance reached 78.0% among jointly significant gene sets and 70.0% (35 of 50) after redundancy collapse (bootstrap 95% CI, 0.560–0.820), coupling neutrophil-granule and myeloid-effector enrichment with depletion of RNA-processing, chromatin, and ciliary programs. Under a contrast-independent maximum interquartile range probe-selection rule, the gene-level overlap was smaller and no longer nominally significant (190 genes; *p* = 0.082), whereas the prespecified 50-program architecture, rescored without re-clustering, retained the same 35 concordant programs. The architecture generalized to acute sleep deprivation (91.4%) and plaque progression (97.1%) but inverted during aneurysm enlargement (20.0%). Prespecified separability was borderline (AUC 0.558; 95% CI, 0.486–0.630; permutation *p* = 0.052). Of twelve topology-prioritized spliceosomal candidates, SF3A3 alone combined FDR-significant exact-cis Mendelian randomization (OR 0.851; 95% CI, 0.772–0.939) with regional colocalization (PP.H4 = 0.831). **Conclusions:** Sleep loss and atherosclerosis converge as coordinated transcriptional programs rather than as shared individual genes, although the magnitude and statistical enrichment of gene-level overlap were sensitive to representative probe selection; this represents cross-compartment transcriptional convergence rather than replication and does not confer individual-level separability. SF3A3 is an experimentally testable candidate, not a validated therapeutic target.

## 1. Introduction

Sleep loss, encompassing insufficient sleep, experimental sleep restriction, and sleep deprivation, is increasingly recognized as a clinically relevant determinant of cardiovascular risk. Its consequences extend beyond impaired vigilance and metabolic homeostasis and include endothelial dysfunction, autonomic imbalance, circadian disruption, metabolic dysregulation, and persistent low-grade inflammation [1,2]. Epidemiological and experimental evidence consistently links short or disturbed sleep with cardiovascular morbidity and mortality, particularly atherosclerotic vascular disease. Large-scale human genetic data further support a biological interaction between sleep and vascular risk. A recent genome-wide gene–sleep interaction study involving more than 730,000 participants identified seventeen loci that modified the association between sleep duration and atherogenic lipid traits, suggesting that the relationship is influenced by shared and potentially modifiable molecular determinants rather than behavioral factors alone [3].

The innate immune system appears to occupy a central position in this relationship. Experimental sleep restriction has been associated with increased circulating neutrophils and monocytes, enhanced production of pro-inflammatory mediators, and altered regulation of hematopoietic progenitor cell activity [4,5]. Sympathetic activation of the bone-marrow niche may contribute to these effects by promoting myelopoiesis and the mobilization of innate immune cells. Circadian regulation of hematopoiesis and leukocyte trafficking provides an additional link between disturbed sleep and vascular inflammation, as time-dependent recruitment of myeloid cells to the arterial wall may facilitate the initiation and progression of atherosclerotic lesions [6]. Within the vascular compartment, monocytes and macrophages exposed to endogenous atherogenic stimuli may undergo persistent epigenetic and metabolic reprogramming, commonly described as trained immunity [7,8]. This process provides a plausible mechanism through which repeated or transient inflammatory perturbations may exert sustained effects on innate immune function and plaque biology. Consistent with this framework, transcriptomic studies of sleep deprivation have identified immune dysregulation and chronic inflammatory signaling among the most prominent molecular alterations [9,10].

These observations raise the possibility that sleep loss and atherosclerosis share a coordinated immune–vascular transcriptional response. Demonstrating such convergence, however, requires more than the numerical overlap of differentially expressed genes. A gene that is increased in one condition but decreased in the other contributes to an overlap count without providing evidence of a directionally shared biological response. Similarly, pathway-level analyses are complicated by the hierarchical structure of annotation databases and by extensive gene sharing among related pathways. Several apparently distinct enrichment results may therefore represent redundant descriptions of the same underlying biological process. Directional concordance and pathway redundancy must consequently be considered before molecular overlap can be interpreted as evidence of coordinated biological convergence.

Systems biology studies of related conditions commonly combine differential expression analysis, weighted gene co-expression network analysis (WGCNA), protein–protein interaction (PPI) networks, immune profiling, and statistical or machine-learning approaches to identify shared genes and prioritize candidate biomarkers or therapeutic targets [11,12,13,14]. A similar strategy has been applied to insomnia and atherosclerosis, including the identification of overlapping disease-associated genes, pathways, and candidate biomarkers [15]. Such approaches have provided useful molecular hypotheses, but gene-list intersection does not establish directional concordance, and extensive overlap among pathway annotations can give disproportionate weight to repeatedly represented biological processes. Moreover, group-level molecular convergence and individual-level discrimination are distinct properties, particularly in studies involving different biological compartments or repeated observations.

We hypothesized that the relationship between experimentally imposed human sleep loss and human atherosclerosis would be more coherently resolved at the level of directionally concordant, redundancy-collapsed transcriptional programs than by numerical overlap of differentially expressed genes alone. We therefore examined convergence sequentially at the levels of individual genes, annotated gene sets, and redundancy-collapsed biological programs, while treating individual-level separability as a distinct inferential question. The resulting program architecture was carried forward without redefinition to an independent acute total-sleep-deprivation cohort and to two additional vascular contexts—atherosclerotic plaque progression and abdominal aortic aneurysm enlargement—to determine whether its directional behavior was preserved across related but biologically distinct settings.

The shared architecture was subsequently placed in biological and pharmacological context through pathway activity analysis, sleep-associated co-expression structure, immune and stromal transcriptional profiling, transcription-factor target enrichment, protein–protein interaction topology, single-cell localization in human atherosclerotic plaque, human genetic analysis, disease–context enrichment, and pharmacological interrogation. The objective was not to infer causality from transcriptomic overlap, nor to derive a diagnostic or therapeutic score, but to determine whether a directionally coherent transcriptional architecture links human sleep loss with atherosclerotic vascular disease and, within that architecture, to identify experimentally testable candidate targets supported by complementary evidence.

## 2. Materials and Methods

### 2.1. Study Design and Transcriptomic Datasets

This study was designed as a multi-cohort analysis of transcriptional programs shared between experimentally induced human sleep loss and human atherosclerotic vascular remodeling. Publicly available human transcriptomic datasets were obtained from the Gene Expression Omnibus (GEO) repository [16,17] under accession numbers GSE39445 [18] (accessed on 1 July 2026), GSE43292 [19] (accessed on 3 July 2026), GSE28829 [20] (accessed on 5 July 2026), GSE57691 [21] (accessed on 7 July 2026), GSE56931 [22] (accessed on 9 July 2026), and GSE253903 [23] (accessed on 26 July 2026).

Because the study involved secondary analysis of publicly available, de-identified transcriptomic data, no participants were recruited directly and no additional institutional ethical approval was required.

Each dataset contributed according to its biological design and sampling structure. GSE39445 served as the sleep loss discovery cohort and comprised repeated whole-blood sampling from healthy participants exposed to controlled sleep restriction and sleep extension protocols across multiple time points [18]. GSE43292 served as the atherosclerosis discovery cohort and comprised paired carotid plaque and macroscopically intact arterial tissue obtained from the same patients [19]. GSE28829 was used to characterize transcriptional changes associated with plaque progression by comparing early and advanced atherosclerotic lesions [20]. GSE57691 was examined as a distinct vascular remodeling cohort comparing large and small abdominal aortic aneurysms (AAAs) [21]. GSE56931 was used to assess directional generalization of the discovery-defined programs under an acute total-sleep-deprivation paradigm [22]. GSE253903 served as the cellular localization cohort and comprised single-cell RNA sequencing (scRNA-seq) profiles from 12 human carotid atherosclerotic plaques; it was used exclusively to examine the cellular distribution of candidate gene and transcriptional program sets defined before inspection of the single-cell results [23].

GSE253903 did not contain either of the principal discovery contrasts: sleep restriction versus sleep extension or carotid plaque versus matched intact arterial tissue. It was therefore not included in differential expression analysis, GSEA, GSVA, WGCNA, cross-cohort directional concordance testing, or discovery-signature construction. These analyses require condition-level contrasts and independent biological replicates, whereas the cells in GSE253903 were nested within 12 plaque samples.

The two discovery cohorts represented different biological compartments: GSE39445 profiled circulating whole blood, whereas GSE43292 profiled carotid arterial tissue. Their comparison was therefore interpreted as cross-compartment transcriptional convergence rather than replication. Expression matrices from different studies were not pooled, and no cross-study batch correction was applied. Cross-cohort comparisons were instead based on shared gene identities, effect directions, ranked test statistics, and pathway-level summaries, thereby avoiding direct integration of expression values generated in different experimental and tissue contexts.

For GSE39445, GSE43292, GSE28829, GSE57691, and GSE56931, analyses began with the processed expression matrices deposited in GEO with the corresponding source studies [18,19,20,21,22]. These datasets were not renormalized from raw intensity files, preserving the preprocessing associated with each study and avoiding the imposition of a second investigator-defined normalization workflow. GSE253903 was handled separately from the deposited sample-level sparse count matrices using the dedicated scRNA-seq workflow described in Section 2.14. Before analysis, normalization status or count-matrix integrity, sample identifiers, phenotype variables, feature identifiers, and expression–metadata alignment were verified programmatically as applicable to each data type. Group assignments were derived from structured GEO metadata and the documented study designs rather than inferred from sample order.

### 2.2. Expression Preprocessing and Probe-to-Gene Annotation

All analyses were conducted in R version 4.6.0. GEO objects and platform annotations were accessed using GEOquery version 2.80.0, and differential expression modeling was performed using limma version 3.68.4 [24,25].

Probe identifiers were mapped to the platform-provided gene symbol fields. Probes lacking a non-empty gene symbol annotation were excluded from gene-level summarization. Gene symbols were retained as deposited, and no feature was removed solely because it represented an uncharacterized locus or a legacy nomenclature entry. For platforms in which a single probe was assigned to multiple gene symbols through a composite annotation, the annotation was expanded before gene-level summarization. In GSE43292, 33,297 probe-level measurements were available, of which 22,195 had a non-empty gene symbol annotation. Expansion of 2143 composite annotations generated 28,819 probe-to-symbol assignments, which were summarized to 21,147 unique gene symbol features. In GSE28829, 54,675 probe-level measurements were available, of which 45,782 had a non-empty gene symbol annotation. Expansion of 2796 composite annotations generated 50,086 probe-to-symbol assignments and 24,442 unique gene symbol features.

GSE39445 contained 43,758 probe-level measurements, including 30,505 probes with a non-empty gene symbol annotation. Differential expression was first estimated at the probe level. For downstream gene-level analyses, one representative probe was retained for each symbol by selecting the probe with the smallest adjusted *p* value and, in the event of an exact tie, the largest absolute log_2_ fold change. This procedure yielded an expression matrix containing 19,541 unique gene symbols. The complete symbol-to-probe map was retained, and the reconstructed gene-level matrix reproduced the corresponding probe-level average-expression values to numerical precision. Because this representative probe rule was informed by full-cohort differential expression results, downstream supervised analyses based on the resulting matrix were interpreted as within-cohort separability analyses rather than as independent prediction assessments. To assess the influence of representative probe selection on downstream inference, a sensitivity analysis was performed in GSE39445 using a contrast-independent probe-selection rule. One representative probe per gene symbol was reselected from the same annotated probe set according to the maximum IQR, and the differential expression model was refitted on the resulting gene-level matrix using the same samples, design matrix, covariates, statistical thresholds, and independently reproduced full-probe consensus within-participant correlation. The GSE43292 arm, gene set search space, and prespecified 50-program membership were held fixed, and no program reconstruction or re-clustering was performed.

GSE57691 contained 39,426 probe-level measurements, of which 36,156 had a non-empty platform-provided gene symbol. One representative probe per symbol was selected according to the maximum interquartile range (IQR), yielding 25,235 unique symbols. Among the retained entries, 5827 represented uncharacterized or legacy locus symbols. The platform annotation contained non-empty Entrez gene and RefSeq fields for all retained rows; this information was recorded as annotation metadata and was not interpreted as evidence that every deposited symbol corresponded to a currently curated gene.

For GSE56931, representative probes were selected using the IQR calculated across the 209 samples included in the primary analysis, with recovery-phase samples excluded from the probe-selection step. The resulting matrix contained 18,725 gene-level features.

Where a composite probe contributed to more than one gene symbol, the resulting gene symbol features were not treated as statistically independent probe measurements. Gene symbol counts and counts of distinct selected-probe measurements were therefore reported separately in the Supplementary Material for the affected platforms.

### 2.3. Differential Expression Analysis

Differential expression was assessed using cohort-specific linear models implemented in the limma framework [24,25,26]. Moderated t-statistics were obtained by empirical Bayes shrinkage of the gene-wise variance estimates [26]. Raw *p* values were adjusted using the Benjamini–Hochberg (BH) false discovery rate (FDR) procedure [27]. Unless otherwise specified, statistical significance required an FDR-adjusted *p* value below 0.05 together with an absolute log_2_ fold change greater than log_2_(1.2). Positive log_2_ fold-change values indicate higher expression in the sleep-restricted or more advanced vascular-disease condition relative to the corresponding reference condition.

For GSE39445, the primary contrast compared sleep restriction with sleep extension. Hours awake and circadian phase were modeled using natural cubic splines with three degrees of freedom. Participant identity was incorporated as a blocking factor through duplicateCorrelation, yielding a consensus within-participant correlation of 0.490. The final analysis included 438 whole-blood samples from 26 participants: 221 samples obtained during sleep extension and 217 during sleep restriction. Twenty-two participants contributed observations under both protocols, whereas four contributed observations under only one protocol.

For GSE43292, the primary contrast compared carotid plaque with paired macroscopically intact arterial tissue. The analysis comprised 64 samples from 32 patients. Tissue identity and patient pairing were verified programmatically from the deposited metadata and the documented study design before model fitting; sample order was not used to assign phenotype. Patient identity was incorporated as a blocking factor using duplicateCorrelation, with a consensus within-patient correlation of 0.272.

For GSE28829, an unpaired two-group model compared advanced with early atherosclerotic plaques. For GSE57691, organ donor and aortic occlusive disease samples were excluded before analysis, and an unpaired model compared large with small AAAs.

To guard against contrast misorientation, a prespecified panel of plaque-enriched genes and markers of the contractile vascular smooth-muscle phenotype was examined after model fitting. This procedure was used solely to verify the biological direction assigned to each contrast and did not alter sample inclusion, model specification, statistical thresholds, or feature selection. Marker definitions and observed coefficients are reported in the Supplementary Material and were not treated as inferential endpoints.

### 2.4. Shared-Gene and Pathway-Level Convergence

The cross-compartment gene universe was defined as the set of gene symbols measured in both discovery cohorts. Enrichment of the overlap between the two differential expression signatures was assessed using the hypergeometric distribution, with the shared measured-gene universe used as the reference population.

Directional agreement among shared differentially expressed genes was evaluated separately from numerical overlap. Exact binomial tests were performed against two prespecified null expectations. The conventional null assumed equal probabilities of concordance and discordance. A second, marginal null incorporated the observed proportions of upregulated and downregulated genes in each cohort. Under this specification, the expected concordant proportion was calculated as the sum of the probabilities of joint upregulation and joint downregulation.

Pathway-level convergence was assessed by gene set enrichment analysis (GSEA) [28] implemented with clusterProfiler [29]. Each discovery cohort was analyzed separately using the complete ranking of moderated t-statistics. Gene sets were obtained from the Molecular Signatures Database (MSigDB) [30] through msigdbr. The tested collection comprised the Hallmark gene sets, curated canonical pathways, and the Gene Ontology (GO), Biological Process (BP), Cellular Component (CC), and Molecular Function (MF) collections. Human Phenotype Ontology gene sets were not included. Gene sets containing fewer than 15 or more than 500 measured genes were excluded before analysis.

Within each cohort, pathway significance was defined by a BH FDR-adjusted *p* value below 0.05. Among gene sets significant in both discovery cohorts, normalized enrichment scores (NESs) were compared to determine directional concordance. Pathway-level concordance was evaluated against both the conventional and marginal null expectations described above. Because gene set collections contain substantial gene overlap and hierarchical redundancy, these gene set-level concordance tests were interpreted as secondary, non-independent summaries rather than as the principal inferential analysis.

### 2.5. Redundancy-Aware Program Reconstruction

Gene sets that were statistically significant in both discovery cohorts were consolidated into redundancy-aware biological programs before the primary concordance assessment. Pairwise similarity was calculated from measured gene membership using both the Jaccard index and the overlap coefficient. The overlap coefficient was included because it is sensitive to nested relationships in which the membership of a smaller gene set is largely contained within that of a larger set.

Hierarchical clustering was evaluated across a prespecified 24-cell grid comprising alternative similarity metrics, linkage methods, and similarity thresholds. The configuration based on the overlap coefficient, average linkage, and a similarity threshold of 0.50 was designated as the reference specification. Results from the remaining configurations were retained as sensitivity analyses and were not used for post hoc selection or optimization.

Within each program, the direction of enrichment in each cohort was defined by the median NES of its constituent gene sets. Each program therefore contributed a single concordance observation, irrespective of the number of related annotations assigned to it. Program-level concordance was prespecified as the principal cross-compartment convergence analysis.

Uncertainty in the observed concordant proportion was quantified using 2000 bootstrap resamples of programs. Because redundancy reduction does not guarantee complete statistical independence among the resulting programs, program-level binomial *p* values were interpreted as nominal summaries. Primary emphasis was placed on the observed concordant proportion, its bootstrap confidence interval (CI), and the stability of the result across the full clustering grid.

### 2.6. Directional Generalization Under Acute Total Sleep Deprivation

The fixed concordant program architecture derived from the discovery analysis was transferred without modification to GSE56931 [22]. This cohort examined acute total sleep deprivation during 38 h of continuous wakefulness and therefore represented a biologically related but methodologically non-equivalent exposure to the controlled sleep-restriction protocol used in GSE39445. Accordingly, GSE56931 was treated as an exploratory assessment of directional generalization rather than as an external validation or replication cohort. The deposited study-day and clock-hour variables were used to reconstruct the experimental phase in accordance with the documented protocol. The primary analysis included 209 samples from 14 participants, comprising 79 baseline samples and 130 samples obtained during total sleep deprivation. Forty recovery-phase samples were excluded from the primary comparison.

The 71 concordant gene sets identified in the discovery analysis were scored using Gene Set Variation Analysis (GSVA) version 2.6.2 [31]. Sample-level gene set scores were then collapsed according to the fixed 35-program structure by calculating, for each sample, the median score across all constituent gene sets assigned to a given program. No gene set selection, reclustering, adjustment of the similarity threshold, or redefinition of program membership was undertaken in GSE56931.

Program scores were analyzed using limma, with clock hour, responder group, and sex included as covariates. Repeated observations were accounted for by incorporating participant identity as a blocking factor. Directional generalization was evaluated by comparing the sign of each estimated program coefficient with the direction defined in the discovery analysis. FDR-adjusted statistical significance was reported separately from directional agreement and was not required for a program to contribute to the directional concordance assessment. A matched-clock sensitivity analysis restricted the sleep-deprivation phase to the first 24 h, thereby aligning the clock-hour range represented under sleep deprivation more closely with that represented at baseline. This analysis was used to determine whether directional agreement was materially influenced by unequal circadian-time coverage between the two study phases.

### 2.7. Within-Cohort Participant-Grouped Separability Analysis

A supervised analysis was conducted in GSE39445 to determine whether the discovery-defined convergent features contained sufficient joint information to distinguish sleep-restriction from sleep-extension samples. This analysis was explicitly framed as a secondary, within-cohort, participant-grouped assessment of separability and was not interpreted as external validation, independent prediction, or diagnostic biomarker development because both the pathway set and the representative probe map had been derived using the same cohort and condition labels.

Three prespecified feature representations were evaluated: the 35 redundancy-collapsed program scores, the 71 individual concordant gene set scores, and the 830 genes in the shared leading-edge intersection. Within this secondary separability analysis, the prespecified reference feature–model combination was LASSO logistic regression applied to the 35 program scores; random forest and radial-kernel support vector machine were evaluated as secondary models.

Model performance was evaluated using five-fold participant-grouped cross-validation repeated five times, with all observations from each participant confined to the same outer fold. For LASSO, the penalty parameter was selected within each outer training set by participant-grouped inner cross-validation. Discrimination was summarized by the area under the receiver operating characteristic curve (AUC). Uncertainty was assessed by participant-cluster bootstrap resampling, and the reference-model AUC was additionally compared with an empirical null distribution generated by participant-blocked label permutations that preserved the participant-level dependence structure, with empirical *p* values calculated using the finite-permutation correction of Phipson and Smyth [32]. The reference analysis used 1000 permutations; secondary feature–model combinations used 100. Full model specifications, resampling procedures, and sensitivity analyses are provided in the Supplementary Methods.

### 2.8. Hallmark Pathway-Activity Profiling in Carotid Plaque

Sample-level pathway activity in GSE43292 was characterized using GSVA [31] applied to the normalized gene-level expression matrix. Scores were calculated for all 50 gene sets in the MSigDB Hallmark collection [30]. Hallmark pathway scores were compared between paired carotid plaque and macroscopically intact arterial tissue using an empirical Bayes-moderated linear model that incorporated patient identity to account for within-patient pairing. Raw *p* values were adjusted using the BH FDR procedure, and Hallmark pathways with an FDR-adjusted *p* value below 0.05 were considered statistically significant. Effect directions were defined relative to plaque tissue: positive coefficients indicated higher pathway activity in plaque, whereas negative coefficients indicated higher activity in the paired intact arterial tissue.

### 2.9. Reproducibility and Audit Trail

All analyses were conducted in R version 4.6.0 under Windows 11. Principal package versions included GEOquery 2.80.0, limma 3.68.4, GSVA 2.6.2, clusterProfiler 4.20.0, msigdbr 26.1.0, glmnet 5.0, randomForest 4.7-1.2, and e1071 1.7-17. Sample assignments, probe-to-symbol maps, program memberships, cross-validation fold assignments, random seeds, model parameters, out-of-fold predictions, bootstrap summaries, permutation distributions, and source-file identities were retained in time-stamped output files. Each analytical run additionally generated a session information record and an entry in the run registry. Empty outputs, zero-result analyses, and statistically nonsignificant findings were preserved and reported rather than replaced through alternative thresholds, selective rerunning, or post hoc model modification.

### 2.10. Weighted Gene Co-Expression Network Analysis

WGCNA was performed in the GSE39445 whole-blood sleep-restriction cohort using the WGCNA R package [33]. To reduce the influence of uninformative low-variance features while preserving broad transcriptomic coverage, the 14,655 genes within the upper 75% of expression variance were retained for network construction. A signed co-expression network was generated using a soft-thresholding power of 7, selected as the lowest power at which the scale-free topology model fit exceeded the prespecified criterion while maintaining adequate mean connectivity. Pairwise gene associations were estimated using biweight midcorrelation, with the maximum proportion of outliers set to 0.10.

Modules were identified in a single block, with the maximum block size set to 16,000 genes, using a signed topological overlap matrix. Dynamic tree cutting was performed with a minimum module size of 30 genes and deepSplit set to 2, followed by module merging at a cut height of 0.25. A fixed random seed was used to ensure computational reproducibility. Genes not assigned to a co-expression module were placed in the grey module and excluded from biological interpretation. Each non-grey module was summarized by its module eigengene, defined as the first principal component of the expression profiles of its constituent genes.

Module–trait associations were evaluated by relating each module eigengene to sleep-restriction status using linear mixed-effects models implemented with lme4 version 2.0-6 and lmerTest version 3.2-1. A participant-level random intercept was included to account for repeated measurements and the within-participant crossover structure. The primary model additionally adjusted for time awake and circadian phase, each represented by a natural cubic spline with three degrees of freedom. A sensitivity model retained only the sleep-restriction term and the participant-level random intercept. Sleep extension served as the reference condition; therefore, a positive regression coefficient indicated higher module eigengene expression during sleep restriction.

Models were fitted by restricted maximum likelihood, with statistical inference based on Satterthwaite approximations to the denominator degrees of freedom. BH FDR correction was applied across the sixteen non-grey modules. Intramodular hub genes were ranked by module membership (kME), defined as the signed biweight midcorrelation between each gene and its corresponding module eigengene.

Because representative probes had been selected using the same sleep-restriction contrast subsequently examined in the module–trait models, the WGCNA was interpreted as an exploratory, hypothesis-generating analysis rather than as an independent validation. Module-level and hub-gene findings were therefore considered within this limitation. One module was characterized by expression close to the detection floor together with a tissue-restricted, non-hematopoietic gene identity. This module was retained in the statistical analysis for completeness but was treated as a likely technical artifact and excluded from biological interpretation. WGCNA version 1.74 was used under R version 4.6.0.

### 2.11. Immune- and Stromal-Cell-Associated Transcriptional Score Analysis

Immune- and stromal-cell-associated transcriptional signals were evaluated in the GSE39445 whole-blood sleep-restriction cohort and the GSE43292 carotid arterial tissue cohort using two complementary marker-based approaches. Microenvironment Cell Populations-counter (MCP-counter; version 1.2.0) was applied using HUGO gene symbols to estimate scores for T cells, CD8 T cells, cytotoxic lymphocytes, natural killer cells, B lineage, monocytic lineage, myeloid dendritic cells, neutrophils, endothelial cells, and fibroblasts [34]. As a complementary approach, prespecified marker scores were calculated for the same ten populations by first standardizing the expression of each marker gene across samples and then averaging the standardized values of the measured genes assigned to the corresponding population. Marker membership was fixed before statistical modeling and was not modified according to the observed results; complete marker gene membership and cohort-specific measurement coverage are reported in the Supplementary Tables. Scores obtained using both approaches were subsequently standardized within each population and are reported in standard deviation units. The GSE39445 analysis used the locked unfiltered expression matrix comprising 19,541 gene symbol features across 438 whole-blood samples from 26 participants. Because the representative probes in this matrix had been selected using the sleep-restriction contrast, the immune score analysis was interpreted as an exploratory within-cohort assessment rather than as a contrast-independent quantification. For each population and scoring approach, the primary analysis used a linear mixed-effects model fitted with lme4 and lmerTest [35,36]. Sleep condition was entered as the exposure of interest, participant identity was included as a random intercept, and hours awake and circadian phase were represented using natural cubic splines with three degrees of freedom. Sleep extension served as the reference condition; consequently, a positive coefficient indicated a higher score during sleep restriction. A reduced sensitivity model retained sleep condition and the participant-level random intercept but omitted the hours-awake and circadian-phase terms. Models were fitted by restricted maximum likelihood, with fixed-effect inference based on Satterthwaite approximations to the denominator degrees of freedom.

The GSE43292 analysis used the locked expression matrix comprising 21,147 gene symbol features across 64 carotid arterial samples from 32 patients, each contributing one atherosclerotic plaque sample and one patient-matched macroscopically intact arterial sample. Standardized population scores were analyzed using patient-paired linear models containing patient identity and tissue group. Intact arterial tissue served as the reference category; therefore, a positive coefficient indicated a higher score in plaque. A corresponding tissue-group-only model was fitted as an unpaired sensitivity analysis. Expression and phenotype records were aligned by sample identifier before modeling and tissue identity was obtained from the documented phenotype information rather than inferred from sample order.

Within each cohort, scoring approach, and model specification, nominal *p* values were adjusted across the complete set of ten populations using the BH FDR procedure. Statistical significance was defined as an adjusted *p* value below 0.05. Within-method robustness was assessed by comparing coefficient directions between the primary and sensitivity models. Cross-method concordance was defined as agreement in coefficient sign between MCP-counter and the prespecified marker score approach. Sample-level agreement between the two approaches in GSE43292 was additionally assessed using Spearman rank correlations, with BH correction across the ten populations.

Cross-cohort integration was performed without pooling the expression matrices or applying cross-study batch correction. For each population and scoring approach, cross-cohort directional concordance required the GSE39445 sleep-restriction coefficient and the GSE43292 plaque coefficient to have the same sign. Four-layer directional concordance required agreement between the two methods within each cohort and directional agreement between the cohorts within each method, such that all four cohort-by-method estimates shared the same sign. This criterion evaluated directional convergence and did not require statistical significance in every constituent analysis. Because MCP-counter and the prespecified marker score approach use partially overlapping marker genes, agreement between the approaches was interpreted as directional and rank consistency rather than as independent methodological validation. Non-hematopoietic scores obtained in whole blood, including fibroblast- and endothelial-associated scores, were treated as marker-based transcriptional signals rather than as evidence of the physical presence of these cell types in the circulation.

### 2.12. Transcription-Factor Enrichment and Regulatory Context Analysis

Transcription-factor (TF) regulatory context was evaluated by over-representation analysis of human TF target gene sets. The primary input comprised 830 genes defined as the intersection between the cohort-specific unions of core-enrichment genes across the 71 directionally concordant gene sets. Two prespecified sensitivity inputs were analyzed in parallel: a 797-gene pathway-matched sensitivity set defined by genes shared within corresponding gene sets across the two discovery cohorts, and a broader 3022-gene union of cohort-specific core enrichment genes. The analytical background was defined separately for each TF target resource and was restricted to genes measured in both discovery cohorts and represented in the corresponding resource, rather than to all annotated human genes.

Three human TF target resources were tested independently: the MSigDB C3:TFT:GTRD collection [37,38], the MSigDB C3:TFT legacy collection, and a locally frozen static human CollecTRI network [39]. Resource identities, database releases, locally retained source files, and checksums were recorded in the analytical audit trail. Target sets containing fewer than 10 or more than 500 background-mapped genes were excluded before testing. For each resource and input definition, enrichment was assessed using a one-sided hypergeometric over-representation test. Nominal *p* values were adjusted within each resource and input definition using the BH FDR procedure, and statistical significance was defined as an adjusted *p* value below 0.05.

Resource-specific mapped query and background sizes were retained and reported explicitly because gene coverage differed across TF target resources. CollecTRI findings were described as regulon enrichment, whereas GTRD and legacy findings were described as regulatory target-signature enrichment. Stability across the three prespecified input definitions and recurrence across independent TF target resources were summarized descriptively and were not used for post hoc selection or modification of the primary result set.

The analysis was interpreted as evidence of regulatory context only. It was not considered evidence of direct TF binding, TF activation or inhibition, causal regulation, mediation, disease specificity, or therapeutic efficacy. In particular, enrichment of a named GTRD target signature was not assumed to establish that the corresponding named entity functioned as an active DNA-binding transcription factor in the underlying biological systems.

### 2.13. PPI Network Construction and Topology-Based Candidate Assessment

The primary network input comprised 830 unique human gene symbols defined a priori as the shared leading-edge intersection across the directionally concordant gene sets. Protein–protein associations were examined using the Search Tool for the Retrieval of Interacting Genes/Proteins (STRING) version 12.0 (https://string-db.org/; accessed on 24 July 2026), restricted to *Homo sapiens* [40]. The network was generated at a minimum required interaction score of 0.700, corresponding to the high-confidence setting, without first- or second-shell interactors. STRING mapped 828 submitted identifiers to distinct protein nodes; *GGT3P* and *HSP90AB3P* were not represented in the downloaded protein-annotation output. Both identifiers were retained in the locked input list, and no substitute identifiers or post hoc replacements were introduced.

Functional enrichment was performed within STRING using GO-BP, CC, and MF annotations, together with Kyoto Encyclopedia of Genes and Genomes (KEGG) and reactome pathways. The whole-genome background was retained, and the complete multiple-testing-adjusted output was exported. For category-level visualization, the ten terms with the highest STRING signal were displayed after term grouping at a similarity threshold of 0.8, whereas the complete enrichment output was retained without graphical selection. STRING associations and enrichment terms were interpreted as evidence of nonrandom functional organization and annotation context rather than as proof of direct physical binding, pathway activation, causal regulation, disease specificity, or therapeutic relevance.

The STRING network was imported into Cytoscape version 3.10.4 running under Java version 17.0.5 [41] and analyzed as an undirected graph. Degree and clustering coefficient were calculated for the complete 828-node mapped network using Analyze Network. The graph comprised 196 connected components, including 189 isolated nodes; the largest connected component contained 626 of the 828 mapped proteins. Betweenness and closeness centrality were calculated within this 626-node component, ensuring that shortest-path-based rankings were derived within a common connected topology. Maximal clique centrality (MCC) was calculated with the cytoHubba application [42] for the complete mapped network to identify proteins embedded within densely interconnected clique structures.

For graphical presentation, a subnetwork containing the 12 highest MCC-scoring proteins was generated. Because the corresponding MCC scores differed only minimally, their internal ordering was not interpreted as a meaningful biological hierarchy. To provide a complementary summary of proteins prominent by both connectivity and network-bridging position, the 20 nodes retained in the Cytoscape degree ranking were intersected with the 20 highest-ranked nodes by betweenness centrality within the 626-node largest connected component. A four-way tie at degree 32 spanned positions 19–22; the order preserved in the Cytoscape export retained HDAC2 within the displayed degree top 20. This tie-handling rule was applied consistently to the comparative topology summary and was not interpreted as an independent validation criterion. Complete degree, clustering-coefficient, and MCC results were retained for all 828 mapped proteins, whereas betweenness and closeness centrality values were reported for proteins within the largest connected component.

Comparable STRING-derived interaction network and Cytoscape-based topology workflows have been applied in related integrative studies [43,44,45,46,47,48,49]. The input definition, identifier mapping rules, interaction threshold, enrichment settings, topological measures, and reporting criteria used in the present analysis were specified for the current dataset and analytical objective.

Network position was not interpreted as evidence of functional essentiality, causal involvement, direct regulatory activity, disease specificity, or therapeutic efficacy. Accordingly, the resulting proteins were described as topology-supported candidate genes rather than as validated hub genes.

### 2.14. Single-Cell Transcriptomic Localization and Cell-Type-Specific Assessment

GSE253903 was used to characterize the cellular distribution of molecular signals identified through the preceding bulk-transcriptomic and network analyses within human carotid atherosclerotic plaque [23]. The analysis included the shared leading-edge gene set, the prespecified MCC-supported candidate set, and the directionally concordant transcriptional programs established before examination of the single-cell data. Cellular localization was evaluated across vascular, stromal, myeloid, and lymphoid compartments at both marker-primary cell-type and Leiden cluster resolution. Sample-level sparse count matrices from the 12 carotid plaque specimens were processed in R version 4.6.0 using Seurat version 5.5.0 and SeuratObject version 5.4.0 [50]. Cell quality was assessed separately within each sample according to library size, detected-feature count, and mitochondrial-transcript percentage. Following quality control, 52,728 cells and 27,709 genes remained. Doublets were identified independently within each GEO Sample (GSM)-defined capture using scDblFinder version 1.26.7 [51], with automatic 10x doublet-rate estimation, no cluster-based prior, and random seed 253,903. Removal of 3540 predicted doublets, followed by exclusion of genes detected in fewer than three retained singlets, yielded the final object containing 49,188 cells and 27,457 genes. All 12 plaque specimens remained represented. The RNA assay was separated into sample-specific layers and log-normalized within each layer using a scale factor of 10,000. Three thousand variable features were selected using the variance-stabilizing transformation method, followed by centering, scaling, and calculation of 50 principal components. Integration was performed in reduced-dimensional space by reciprocal principal component analysis (RPCA) through IntegrateLayers, using dimensions 1–30, 30 integrated dimensions, k.anchor = 5, and k.filter = 200. The integrated representation was used for neighborhood construction, clustering, and uniform manifold approximation and projection (UMAP) visualization, whereas gene- and program-level expression summaries were calculated from the nonintegrated log-normalized RNA data.

A shared nearest neighbor (SNN) graph was constructed from integrated dimensions 1–30 using 30 nearest neighbors, Annoy search with 50 trees, Euclidean distance for graph construction, and an SNN-pruning threshold of 1/15. UMAP was generated with the R-native uwot version 0.2.4 implementation [52] using 30 neighbors, cosine distance, min.dist = 0.30, spread = 1, and random seed 253,903. Leiden clustering [53] was evaluated across resolutions 0.2–1.0 in increments of 0.1 using algorithm 4, the modularity objective function, and 10 iterations. Resolution 0.5 was selected as the lowest solution meeting the prespecified criteria of an adjusted Rand index of at least 0.90 relative to both adjacent resolutions, a minimum cluster size of 100 cells, and representation of more than one GSM accession within every cluster. This procedure yielded 19 Leiden clusters. Positive cluster markers were identified from the log-normalized RNA data using the Wilcoxon rank-sum test and a minimum log_2_ fold-change threshold of 0.25. Cellular identities were assigned through a marker-primary framework based on canonical vascular, stromal, myeloid, and lymphoid lineage markers. Marker expression was averaged within each cluster and standardized by gene across clusters, after which marker group scores were calculated from the corresponding standardized profiles. The highest-scoring marker group defined the primary assignment, while the second-ranked group and score margin supported interpretation of closely related cellular states. The resulting annotation framework comprised endothelial cells, smooth muscle cells, fibroblasts, foam cells, macrophages, dendritic cells, neutrophils, mast cells, T cells, NK cells, B cells, and plasma cells.

Cluster-level SingleR version 2.14.0 [54] classifications based on the Human Primary Cell Atlas and Blueprint/ENCODE reference collections obtained through celldex version 1.22.0 provided an orthogonal assessment of the marker-primary annotations. Final cell identities remained grounded in canonical lineage marker expression and cluster-level transcriptional context, and concordance with each reference collection was recorded separately. For each gene represented in the final expression matrix, average log-normalized expression and detection frequency were calculated across the marker-primary cell types. Detectable expression was defined as a normalized RNA value greater than zero. The cell type with the highest average expression was recorded as the predominant cellular localization, and average expression values were standardized separately for each gene across cell types to generate gene-wise z-scores. These measures described complementary aspects of cellular distribution: relative enrichment, expression magnitude, and the proportion of cells in which a transcript was detected. The prespecified MCC-supported candidates were examined using the same framework and in the order established by the network analysis. In the dot-plot representation, point color encoded average log-normalized expression and point area encoded detection frequency. Point-size scaling was capped at 80% detection to preserve visual discrimination among highly prevalent transcripts; the underlying detection values were unchanged. Candidate expression was also summarized across the 19 Leiden clusters, with the dominant marker-primary identity of each cluster retained as an annotation layer. This cluster-resolved analysis was designed to distinguish compartment-wide expression from localization within particular transcriptional subpopulations.

Cellular localization of the directionally concordant transcriptional programs was derived from their established gene set memberships. Constituent gene sets were mapped to unique human genes, and only genes represented in the final expression matrix contributed to scoring. For each program, average log-normalized expression of every measured member gene was calculated across marker-primary cell types and standardized across cell types. The mean of these gene-level standardized values defined the cellular localization score, which was subsequently standardized within each program for comparative visualization. This framework placed independently defined candidate genes and transcriptional programs within the cellular architecture of human carotid plaque. The resulting patterns were interpreted as cellular contextualization rather than as condition-level differential expression evidence or direct pharmacological validation.

### 2.15. Human Genetic Support, Exact-Cis Mendelian Randomization (MR), Regional Colocalization, and Disease Enrichment

Human genetic support was evaluated for the 12 candidate genes defined a priori by the preceding MCC analysis: *SNRPF*, *SNRPD1*, *SNRPB2*, *CWC22*, *SF3B2*, *CWC27*, *BUD13*, *SF3B1*, *SF3A3*, *SNIP1*, *PRPF38A*, and *RBMX2*. Gene-specific expression quantitative trait locus (eQTL) datasets from the eQTLGen phase I whole-blood eQTL resource [55] were accessed through their corresponding OpenGWAS exposure records (https://gwas.mrcieu.ac.uk/; accessed on 25 July 2026) [56]. The primary outcome was the European ancestry coronary artery disease (CAD) summary statistics dataset deposited under GWAS catalog accession GCST003116 and indexed in OpenGWAS as ebi-a-GCST003116 [57]. This dataset was mapped to the GRCh37/hg19 reference build and comprised 141,217 participants, including 42,096 cases. It represents the European ancestry dataset deposited under GCST003116 and linked to the CARDIoGRAMplusC4D meta-analysis reported by Nikpay et al., rather than the complete publication-wide meta-analysis sample. Because the control count field deposited in OpenGWAS was internally inconsistent with the reported total sample size and case count, the number of controls was derived as 141,217 minus 42,096, yielding 99,121 controls. This derived value, rather than the deposited control count field, was used in all case–control metadata-dependent calculations, including liability-scale Steiger directionality analyses and effective outcome sample-size sensitivity analyses.

Candidate gene coordinates were mapped to GRCh37/hg19 using TxDb.Hsapiens.UCSC.hg19.knownGene version 3.22.1. Variants were classified as exact cis instruments when they were located on the same chromosome as the corresponding candidate gene and within 1 Mb of the annotated gene boundaries. Exposure associations were required to achieve genome-wide significance at *p* < 5 × 10^−8^. Linkage-disequilibrium clumping was performed after exact-cis restriction using a European reference panel, an *r*^2^ threshold < 0.001, and a 10,000 kb window. Instrument strength was quantified as F = (β/SE)^2^, where SE denotes the standard error, and variants with F ≤ 10 were excluded. CAD associations were retrieved only for the exact exposure variants, without linkage disequilibrium proxy substitution. Exposure and outcome alleles were harmonized using TwoSampleMR version 0.7.9 [58] with action = 2, thereby applying conservative allele-frequency handling to palindromic variants; variants excluded during harmonization were documented and were not replaced.

MR analyses were implemented in R version 4.6.0 using the TwoSampleMR package version 0.7.9 [58], with OpenGWAS access and associated data operations performed using ieugwasr version 1.1.0.9000, a development build [56]. For candidate genes represented by a single harmonized instrument, the Wald ratio estimator was applied. Inverse-variance weighting [59] was used as the primary estimator when at least two independent instruments were available. Weighted median [60] and MR-Egger [61] analyses were additionally performed when at least three independent instruments remained. Primary *p* values were adjusted across the complete set of 12 locked candidate genes using the BH procedure; candidates lacking an estimable instrument were retained within the prespecified multiplicity family and explicitly identified as non-estimable. Cochran’s Q heterogeneity tests, MR-Egger intercept tests, single-SNP estimates, and leave-one-out analyses were performed whenever permitted by the number of available instruments. The primary analysis was repeated after excluding all palindromic variants.

Steiger directionality [62] was evaluated under assumed CAD population prevalences of 0.05, 0.10, and 0.15. For each prevalence assumption, the analysis was repeated using both the total outcome sample size and the effective case–control sample size calculated from 42,096 cases and 99,121 derived controls. Regional colocalization was performed for *SF3A3*, *SNIP1*, and *SNRPF*, the three candidates showing nominal evidence in the primary MR screen. For each candidate, dense exposure and outcome summary statistics were extracted within 500 kb on either side of the lead exposure variant, and only SNPs represented in both datasets were retained. Colocalization was performed using coloc version 5.2.3 and the coloc.abf function [63] under the single-causal-variant assumption. The exposure was specified as a quantitative trait using SNP-level effect estimates, effect-estimate variances, minor-allele frequencies, and exposure sample sizes. CAD was specified as a case–control outcome using *N* = 141,217 and a case fraction of 42,096/141,217. Prior probabilities for association with trait 1 and trait 2 were each set to 1 × 10^−4^. The primary prior probability of a shared association was set to *p*_12_ = 1 × 10^−5^, with sensitivity analyses conducted at 1 × 10^−6^ and 5 × 10^−5^. Posterior probabilities (PPs) for hypotheses H0–H4, together with the conditional colocalization metric PP.H4/(PP.H3 + PP.H4), were calculated under each prior setting. MR and colocalization findings were interpreted as genetically informed support and not as proof of causality, target validation, or therapeutic efficacy.

To place the transcriptomic findings within a broader disease context, disease enrichment analysis was performed using the 830 locked shared leading-edge genes. One-sided hypergeometric over-representation analysis was conducted locally in R using custom scripts. The enrichment universe comprised 16,674 unique HUGO Gene Nomenclature Committee (HGNC)-recognized genes measured in the study, with duplicate gene symbols counted once. Disease-oriented gene set definitions were obtained through Enrichr [64] from DisGeNET [65], Jensen_DISEASES_Experimental_2025 [66], GWAS_Catalog_2025 [67], ClinVar_2025 [68], and OMIM_Disease [69]. Terms represented by fewer than five genes within the custom background were excluded. BH correction was applied separately within each library across all eligible terms, and an adjusted *p* value < 0.05 was considered statistically significant.

The prespecified disease domains were atherosclerosis, CAD, myocardial infarction, peripheral arterial disease, carotid atherosclerosis, insomnia, sleep deprivation or sleep loss, sleep duration or short sleep, and circadian rhythm or sleep disorders. Candidate terms were retained only when their clinical interpretation corresponded to the relevant prespecified domain. Substring-only false-positive matches and exact cross-domain duplicates were excluded, and no disease term was manually introduced. Because the 830-gene set was analyzed without regard to expression direction, disease-enrichment findings were interpreted as disease-associated gene set overlap and biological context rather than as evidence of directional regulation, causal inference, target validation, or therapeutic efficacy.

### 2.16. Cross-Dataset and Disease Context Sensitivity Analyses

The disease context robustness of the locked sleep loss–atherosclerosis transcriptional architecture was examined in two additional vascular comparisons: advanced versus early carotid plaque in GSE28829 and large versus small AAA in GSE57691. The purpose of these analyses was to determine whether the direction of the prespecified program architecture was retained across related but biologically distinct vascular trajectories. Accordingly, the analyses were interpreted solely as assessments of cross-dataset directional and pathway concordance across distinct vascular disease contexts.

GSE28829 included 16 advanced and 13 early plaque specimens. The locked gene-level differential expression results were carried forward without recomputation. GSE57691 included 29 large and 20 small AAA specimens; samples from aortic occlusive disease and organ-donor aorta were excluded from this contrast. For GSE57691, one representative probe was selected per gene symbol according to the prespecified maximum-IQR rule across the 49 AAA samples, with probe identifier used to resolve ties. The retired platform symbol *SDCCAG10* was harmonized to the current symbol *CWC27*. Differential expression was estimated with limma using an unpaired large-versus-small contrast. Reconstructed estimates were compared with the locked GSE57691 results to confirm agreement in log_2_ fold change, moderated *t* statistic, and rank ordering of the reported genes.

The primary analysis was performed at the level of the 35 fixed transcriptional programs derived from the discovery stage redundancy reduction. Program membership, constituent gene sets, and expected directions were retained without modification. Collectively, the 35 programs comprised the 71 locked concordant gene sets. GSEA was conducted separately for each disease context contrast using the complete ranking of moderated *t* statistics and the frozen gene set collection. Program direction was defined by the sign of the median NES across its constituent gene sets. A program was classified as directionally concordant when its context-specific direction agreed with its prespecified GSE43292 reference direction. This classification did not require statistical significance of the constituent gene sets, because the analysis addressed preservation of direction rather than independent redetection of enrichment. The 95% CI for the proportion of concordant programs was estimated from 5000 nonparametric bootstrap resamples of the 35 programs.

A secondary analysis evaluated the 830 locked shared leading-edge genes used in the network analysis. Within each disease context, gene-level concordance was summarized as the proportion of evaluable genes whose log_2_ fold-change direction agreed with the corresponding GSE43292 reference effect. The relationship between context-specific and reference effects was further quantified by Spearman rank correlation. Because these genes were derived from overlapping gene sets and correlated biological modules, they were not treated as independent inferential units; no binomial or direct cross-resolution significance test was therefore applied.

The 12 topology-supported candidates—*SNRPF*, *SNRPD1*, *SNRPB2*, *CWC22*, *SF3B2*, *CWC27*, *BUD13*, *SF3B1*, *SF3A3*, *SNIP1*, *PRPF38A*, and *RBMX2*—were examined as a prespecified descriptive layer. For each candidate, the context-specific log_2_ fold change, moderated *t* statistic, nominal and adjusted *p* values, reference and observed directions, directional agreement, and percentile ranks of the absolute log_2_ fold change and moderated *t* statistic were recorded. No candidate-level hypothesis test was performed because the candidates arose predominantly from the same densely interconnected spliceosomal network and were not considered statistically independent observations.

Analyses were performed in R version 4.6.0. Differential expression modeling used limma version 3.68.4, GSEA used clusterProfiler version 4.20.0, and GEO series matrices and platform annotations were accessed using GEOquery version 2.80.0. Gene set definitions were taken from the frozen collection generated with msigdbr version 26.1.0 and were retained unchanged throughout the analysis.

### 2.17. Multilayer Characterization of Prespecified Candidate Targets

The 12 MCC-supported candidates emerging from the preceding network topology analysis—*SNRPF*, *SNRPD1*, *SNRPB2*, *CWC22*, *SF3B2*, *CWC27*, *BUD13*, *SF3B1*, *SF3A3*, *SNIP1*, *PRPF38A*, and *RBMX2*—were retained as a prespecified candidate set and were not subjected to further selection, re-ranking, or composite scoring. Candidate-level characterization was undertaken across complementary analytical domains encompassing network topology, sleep-associated co-expression structure, regulatory context overlap, plaque cell expression, exact-cis MR, regional colocalization, and transcriptional behavior during plaque progression.

Sleep-associated co-expression context was defined by membership in the retained WGCNA modules. Signed own-module membership (kME) was recorded for candidates represented in these modules, with kME ≥ 0.80 prespecified as indicating high intramodular membership. Lack of membership in a retained sleep-associated module was interpreted strictly as absence from that filtered module subset and not as absence from the broader WGCNA network or as evidence against support from other analytical layers. Regulatory context was assessed by overlap with the FDR-significant primary transcription factor target signatures. Plaque cell context was summarized according to the cell population showing the highest average expression for each candidate in the human atherosclerotic plaque single-cell dataset.

Primary exact-cis MR estimates were reported as odds ratios (ORs) with 95% CIs, nominal *p* values, and BH-adjusted *p* values across the prespecified 12-candidate analysis universe. Regional colocalization was restricted to the three candidates advanced a priori from the genetic analysis and was interpreted under the primary shared-signal prior, while retaining the relevant prior-sensitivity qualifications. Plaque progression behavior was evaluated relative to the locked reference effect and was summarized descriptively as directional concordance or divergence; such concordance was not equated with candidate-level differential expression significance.

### 2.18. Construction of the Candidate–Program–Plaque Cell Framework

The 12 prespecified MCC-supported candidates were integrated with the locked sleep loss–atherosclerosis transcriptional architecture without further selection or re-ranking. The analytical universe comprised the 71 gene sets showing concordant enrichment direction across the sleep loss and atherosclerosis analyses, organized into 35 fixed transcriptional programs under the locked reference clustering specification based on the overlap coefficient, average linkage, and a threshold of 0.50.

Candidate–gene set relationships were defined by direct membership of a candidate in the leading-edge genes of a locked constituent gene set. Candidate–program relationships were subsequently established according to the fixed program assignment of each candidate-containing gene set. Membership in the broader 830-gene intersection, in the absence of direct leading-edge membership, was not considered sufficient to define a candidate–program relationship. When multiple constituent gene sets linked the same candidate to the same program, these relationships were collapsed at the candidate–program level while retaining the number and identity of the supporting gene sets.

Leading-edge membership was evaluated independently in the sleep loss and atherosclerosis analyses. A candidate–gene set relationship was classified as supported in both cohort-specific maps when the candidate occurred in the leading edge of the same locked gene set in both analyses, and as supported in one cohort-specific map when it occurred in only one. This annotation was applied after link construction and did not affect retention of the candidate–program relationship.

Candidate-level plaque cell context was defined by the cell population showing the highest average expression for each candidate in the human atherosclerotic plaque single-cell dataset. Program-level cellular context was derived independently from the predominant plaque cell localization assigned to each fixed transcriptional program. Candidate–program pairs were classified as cellularly concordant when the candidate and program shared the same predominant plaque cell population and as cellularly distinct when their predominant cellular assignments differed.

Sleep-associated WGCNA membership, regulatory context overlap, primary exact-cis MR, regional colocalization, and plaque-progression directionality were incorporated subsequently as candidate-level contextual annotations. These layers were not used to define, weight, exclude, or rank candidate–program relationships.

### 2.19. Perturbation Signature Prioritization Using the LINCS L1000 Resource

Compound perturbation signatures were interrogated using the LINCS L1000 Phase II resource (GSE70138) [70]. The release-matched Level 5 moderated z-score matrix and accompanying metadata were restricted a priori to small-molecule perturbations (trt_cp), without post hoc selection by cell line, dose, exposure duration, signature quality metric, or reversal magnitude. Analyses were confined to this release. Cohort-specific queries comprised the locked differential expression statistics from the sleep restriction and carotid atherosclerosis discovery datasets, restricted to the prespecified 830-gene shared leading-edge set. Of these genes, 622 were represented in the best-inferred gene (BING) space, including a nested subset of 68 directly measured landmark genes. The 622-feature BING representation constituted the principal perturbational gene space, whereas the locked 68-feature landmark subset was analyzed separately as a directional sensitivity representation.

Transcriptional reversal was quantified for each eligible LINCS signature as the negative Spearman correlation between the cohort differential expression vector and the corresponding perturbation signature vector across represented query genes; positive values therefore indicated opposition to the cohort-associated transcriptional pattern. Signature-level prioritization required both a positive reversal score and placement within the upper 5% of the corresponding release-relative score distribution. This percentile criterion was deliberately descriptive and was not treated as a threshold for statistical significance. Perturbagen-level prioritization required at least one qualifying signature in each discovery cohort. Program-level reversal was evaluated separately against the fixed 35-program architecture and served only to provide mechanistic context to perturbagen prioritization. No composite compound score, cross-layer weighting scheme, therapeutic ranking, or assessment of clinical actionability was constructed.

Calibration and sensitivity analyses assessed permutation-null behavior, covariance-related effective dimensionality using the participation ratio [71], fixed-size comparator subsets, and alternative correlation block specifications. These analyses were undertaken to characterize the statistical behavior and dependency structure of the LINCS gene spaces rather than to generate an additional inferential criterion for compound selection. Accordingly, permutation-derived *q* values were retained as calibration annotations, did not determine perturbagen membership, and were not interpreted as evidence of statistical significance. Detailed calibration families, permutation procedures, effective dimensionality analyses, fixed-size comparator analyses, and sensitivity specifications are provided in the Supplementary Methods.

Two structural dependencies were considered integral to interpretation of the perturbational analysis. First, perturbagen-level reversal scores were strongly correlated between the two discovery cohorts; concordance across these queries was therefore treated as descriptive cross-cohort prioritization and not as independent pharmacological replication. Second, perturbagen membership was inherently influenced by representation within the LINCS release and was therefore reported without coverage-adjusted weighting, re-ranking, or post hoc threshold modification. Importantly, transcriptional reversal and pharmacological target annotation were maintained as distinct evidentiary layers. The LINCS analysis was not used to infer direct target engagement, inhibition, activation, or causal target modulation, and a Drug–Gene Interaction Database (DGIdb)-recorded drug–gene interaction (Section 2.20) was not taken as evidence that the corresponding compound reverses either disease-associated transcriptional pattern. The LINCS-derived perturbagens were consequently regarded as hypothesis-generating, release-relative priorities for experimental interrogation—not as validated drug candidates, evidence of target tractability, or predictions of therapeutic efficacy—consistent with recognized limitations of transcriptomics-based drug repurposing in cardiovascular disease [72].

### 2.20. DGIdb-Based Pharmacological Interaction Annotation

Pharmacological interaction context was examined independently of the LINCS perturbation signature analysis using DGIdb version 5.0.11 5.0.11 (https://www.dgidb.org/; accessed on 2 August 2026) [73]. The analysis was restricted to the locked set of 830 shared leading-edge genes, within which the 12 prespecified MCC-supported candidates were retained as a nested subset. DGIdb was queried through the live GraphQL endpoint. Returned records were consolidated at the gene–drug-pair level, with duplicate records arising from multiple contributing sources represented as a single interaction pair while preserving the associated source information, publication identifiers, interaction scores, approval annotations, and DGIdb-provided interaction type and directionality where available.

To distinguish evidence of pharmacological target engagement from associations arising primarily from pharmacogenomic or variant-response resources, contributing DGIdb sources were classified a priori as target-engagement or variant-response sources. Gene–drug pairs supported by both source classes were designated as mixed evidence. This distinction was used to characterize the provenance of the pharmacological evidence rather than to impose an additional selection criterion. Accordingly, all returned interactions were retained irrespective of interaction score, approval status, or availability of directionality information, and genes for which no interaction was returned remained explicitly represented in the analysis.

The 12 MCC-supported candidates were interrogated without alteration of their prespecified identity or order. Candidate-level annotation was descriptive and was not used to select or re-rank candidates. DGIdb evidence was interpreted as pharmacological annotation rather than as evidence of therapeutic efficacy. In particular, a database-recorded gene–drug relationship does not establish target engagement in the biological context examined here, whereas failure to retrieve an interaction does not establish that a gene is pharmacologically intractable. The DGIdb layer was therefore kept analytically separate from LINCS transcriptional reversal, network topology, genetic evidence, and plaque cell localization. No cross-layer compound target score, therapeutic ranking, or assessment of clinical actionability was derived from these annotations.

## 3. Results

### 3.1. Transcriptional Response to Experimentally Imposed Sleep Restriction

The transcriptional response to experimentally imposed sleep restriction was characterized in the GSE39445 discovery cohort using a limma model that accounted for repeated measurements within participants and adjusted explicitly for hours awake and circadian phase. Differential expression was estimated initially at the probe level. For downstream gene-level analyses, one representative probe was retained for each gene symbol according to the prespecified post-model selection rule: the probe with the smallest adjusted *p* value was selected, with the largest absolute log_2_ fold change used to resolve ties. This procedure yielded 19,541 unique gene symbol features from 43,758 probe-level measurements (Table 1 and Table S1).

**Figure 1:**
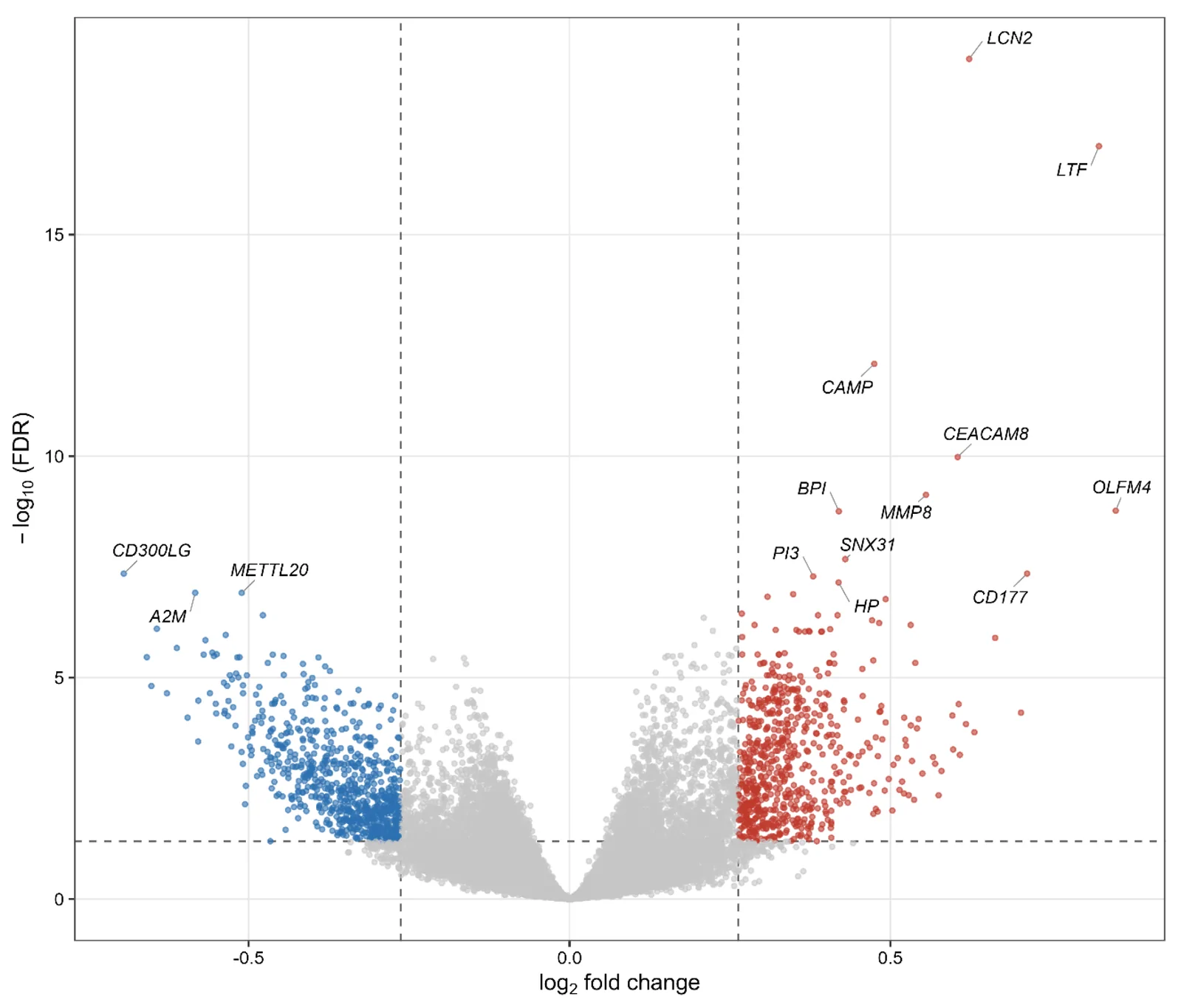
Gene-level differential expression landscape of the GSE39445 sleep-restriction discovery cohort. Volcano plot of gene-level differential expression during sleep restriction relative to sleep extension. Each point represents one gene symbol retained after deterministic probe-to-gene summarization. Genes meeting both prespecified criteria—a BH FDR-adjusted *p* value below 0.05 and |log_2_ fold change| > log_2_(1.2)—are distinguished according to effect direction. Positive log_2_ fold-change values indicate higher expression during sleep restriction, whereas negative values indicate higher expression during sleep extension. Vertical dashed lines denote the effect size threshold, and the horizontal dashed line denotes the FDR threshold. Representative highly significant genes are annotated. Red points indicate significantly upregulated genes, blue points indicate significantly downregulated genes, and grey points indicate genes that did not meet both prespecified significance criteria.

A total of 4390 genes met the BH FDR threshold of 0.05. Of these, 1352 also exceeded the prespecified effect size threshold of |log_2_ fold change| > log_2_(1.2); 645 showed higher expression and 707 showed lower expression during sleep restriction relative to sleep extension (Figure 1 and Figure S1; Table 1 and Table S2). Among the most prominent responses, *LCN2* was increased by a log_2_ fold change of 0.62 (adjusted *p* = 1.09 × 10^−19^), whereas *LTF* was increased by 0.82 (adjusted *p* = 1.01 × 10^−17^). Differentially expressed genes were distributed across the full range of average expression rather than being confined to the most highly expressed transcripts, as shown by the volcano and mean difference plots (Figure 1 and Figure S1).

Because representative probe selection was informed by the full-cohort differential expression results, the resulting gene-level matrix was used for biological characterization and within-cohort downstream analyses rather than interpreted as an independently derived predictive feature set.

### 3.2. Transcriptional Remodeling in Human Carotid Atherosclerotic Plaque

**Figure 2:**
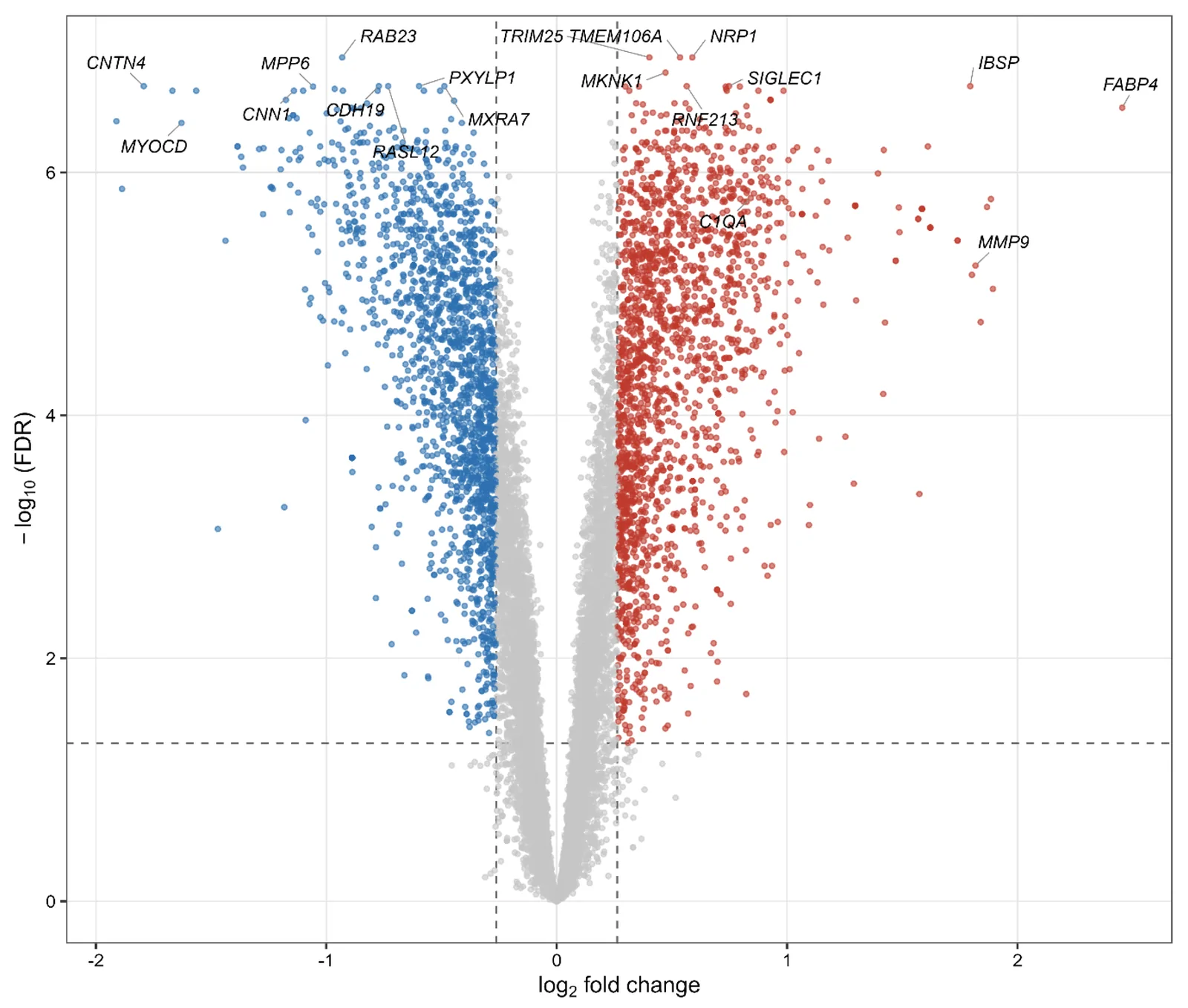
Gene-level differential expression landscape of paired carotid plaque and macroscopically intact arterial tissue in GSE43292. Volcano plot of gene-level differential expression in carotid plaque relative to paired intact arterial tissue. Each point represents one gene symbol feature retained after expansion of composite annotations and deterministic gene-level summarization. Genes meeting both prespecified criteria—a BH FDR-adjusted *p* value below 0.05 and |log_2_ fold change| > log_2_(1.2)—are distinguished according to effect direction. Positive log_2_ fold-change values indicate higher expression in plaque, whereas negative values indicate higher expression in paired intact tissue. Vertical dashed lines denote the effect size threshold and the horizontal dashed line denotes the FDR threshold. Representative highly significant genes are annotated. Red points indicate genes significantly upregulated in plaque tissue, blue points indicate genes significantly upregulated in paired intact arterial tissue, and grey points indicate genes that did not meet both prespecified significance criteria.

The atherosclerosis discovery analysis was conducted in GSE43292 using paired carotid plaque and macroscopically intact arterial tissue obtained from 32 patients. Patient pairing was incorporated explicitly in the statistical model. Following expansion of composite platform annotations and deterministic gene-level summarization, 33,297 probe-level measurements yielded 21,147 unique gene symbol features. Of these features, 8012 met the BH FDR threshold, and 3719 additionally exceeded the prespecified effect size criterion. Among the latter, 1946 genes showed higher expression and 1773 showed lower expression in plaque relative to paired intact arterial tissue (Figure 2 and Figure S2; Table 1, Tables S3 and S4). The biological orientation of the contrast was examined using a prespecified sentinel panel. Plaque-associated genes, including *FABP4* (log_2_ fold change +2.45; adjusted *p* = 2.9 × 10^−7^), *MMP9* (+1.82), and *C1QA* (+0.84), showed higher expression in plaque, whereas markers of the contractile vascular smooth-muscle phenotype were reduced, including *MYOCD* (−1.63; adjusted *p* = 3.9 × 10^−7^) and *CNN1* (−1.14). Differential expression extended across the full range of average expression, as illustrated by the volcano and mean difference plots (Figure 2 and Figure S2).

### 3.3. Transcriptional Changes Associated with Atherosclerotic Plaque Progression

**Figure 3:**
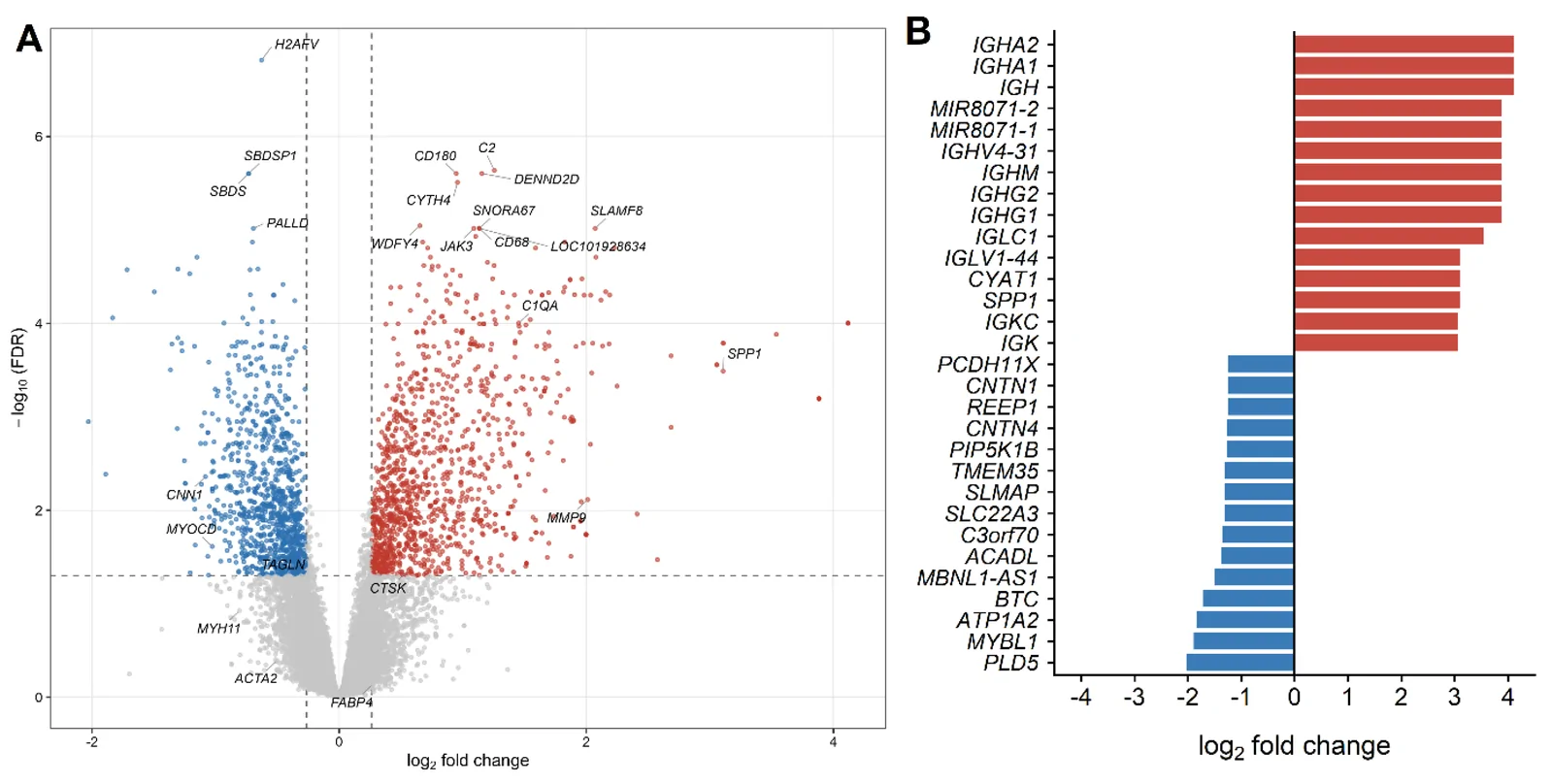
Differential expression landscape associated with atherosclerotic plaque progression in GSE28829. (**A**) Volcano plot of gene-level differential expression in advanced relative to early atherosclerotic plaques. In panel A, each point represents one gene; the vertical dashed lines indicate the prespecified effect-size thresholds, and the horizontal dashed line indicates the FDR threshold. Red and blue points denote genes meeting both criteria with higher and lower expression, respectively, in advanced plaque, whereas grey points denote genes not meeting both criteria. (**B**) Fifteen genes with the largest positive and fifteen with the largest negative log_2_ fold changes among genes meeting both criteria. Positive values indicate higher expression in advanced plaque, whereas negative values indicate lower expression in advanced plaque.

GSE28829 was analyzed to characterize transcriptional differences between advanced and early human atherosclerotic plaques. Following expansion of composite platform annotations and deterministic gene-level summarization, 54,675 probe-level measurements yielded 24,442 unique gene symbol features. Of these, 2342 met the BH FDR threshold, and 2218 additionally exceeded the prespecified effect-size criterion. Among the latter, 1202 genes showed higher expression and 1016 showed lower expression in advanced relative to early plaques (Figure 3; Table 2 and Tables S5–S8).

All eleven genes included in the prespecified contrast orientation panel changed in the biologically expected direction. *FABP4* (+0.27), *CD68* (+1.14), *MMP9* (+2.01), *C1QA* (+1.45), *SPP1* (+3.11), and *CTSK* (+0.56) were higher in advanced plaques, whereas *MYOCD* (−1.03), *CNN1* (−1.08), *ACTA2* (−0.50), *MYH11* (−0.80), and *TAGLN* (−0.63) were lower. Directional agreement did not, however, imply statistical significance for every marker: the adjusted *p* values for *FABP4*, *ACTA2*, and *MYH11* were 0.73, 0.41, and 0.12, respectively. Because some composite probes contributed to more than one gene symbol after annotation expansion, the 24,442 gene symbol features corresponded to 22,807 distinct selected-probe measurements. Likewise, the 2218 genes meeting both statistical criteria corresponded to 2091 distinct selected-probe measurements. These quantities are reported separately to avoid treating duplicated probe measurements arising from composite annotations as independent molecular observations (Table S7).

### 3.4. Context-Specific Aneurysm Severity Analysis in GSE57691

GSE57691 was examined as a distinct vascular remodeling cohort rather than as a replication dataset. The primary analysis compared 29 large with 20 small AAAs after exclusion of nine aortic occlusive disease samples and ten organ donor samples. Gene-level processing retained 25,235 unique platform-provided symbols.

No gene reached the prespecified BH FDR threshold, and no gene met the combined FDR and effect size criterion. The smallest unadjusted *p* value was 7.7 × 10^−4^, whereas the smallest adjusted *p* value was 0.99997 (Figure S3; Table 3, Tables S9 and S10).

The absence of FDR-significant genes was unchanged across four probe-to-gene summarization rules and four variance specifications (Table S11), indicating that the result was not attributable to the primary collapsing procedure alone. Nevertheless, the lack of statistically significant differential expression was not interpreted as evidence of equivalence between large and small aneurysms.

Under 1000 label permutations preserving the observed group sizes, 56 genes had nominal *p* values below 0.05 in the observed analysis, compared with a null median of 276. The corresponding lower-tail empirical *p* value was 0.069. The dispersion of the observed moderated t-statistics was also not distinguishable from its permutation distribution (lower-tail empirical *p* = 0.084). These findings indicate that the observed differential expression pattern was compatible with the broad label-exchangeable null generated from the processed matrix. They do not establish biological equivalence, exclude smaller or more heterogeneous effects, or identify the mechanism underlying the conservative distribution of test statistics. GSE57691 was therefore retained as a context-specific negative result rather than excluded from the analytical narrative.

### 3.5. Gene-Level Overlap Lacks Directional Concordance, Whereas Pathway-Level Alignment Is Substantially Stronger

The two discovery cohorts were compared at the levels of individual genes, annotated gene sets, and redundancy-collapsed biological programs. Among the 16,869 genes measured in both cohorts, 274 met the prespecified differential expression criteria in both datasets, compared with 228.35 expected under independence. The observed overlap was therefore greater than that expected by chance (hypergeometric *p* = 3.54 × 10^−4^).

The shared differentially expressed genes did not, however, show coherent directional agreement. Of the 274 overlapping genes, 134 changed in the same direction in both cohorts, corresponding to 48.9% concordance. This proportion did not differ from the conventional null expectation of 0.5 (*p* = 0.66) or from the marginal null expectation of 0.5185 derived from the observed up- and downregulation frequencies in the two cohorts (*p* = 0.85). Across the full shared measured-gene universe, effect estimates were only weakly correlated (Spearman ρ = 0.095). Thus, although the two cohorts shared more differentially expressed genes than expected under independence, the directions of those gene-level changes were not coherently aligned (Tables S12 and S13).

A markedly different pattern emerged at the pathway level. Of the 8284 gene sets evaluable in both discovery cohorts, 91 were significant in both. Seventy-one of these 91 gene sets, corresponding to 78.0%, showed concordant enrichment directions: 45 were positively enriched in both cohorts and 26 were negatively enriched in both. The corresponding exact binomial *p* values were 3.6 × 10^−8^ against the conventional null and 4.6 × 10^−8^ against the marginal null (Tables S14 and S15).

Leave-one-gene set-out analysis yielded concordance estimates ranging from 77.8% to 78.9%, indicating that the observed proportion was not driven by any single gene set. NESs among the 91 jointly significant gene sets were positively correlated between the two cohorts (Spearman ρ = 0.595; *p* = 4.9 × 10^−10^). Because these gene sets are not statistically independent owing to shared genes and hierarchical annotation structure, the binomial tests were interpreted as secondary summaries rather than as the principal inferential evidence. Within this limitation, coordinated pathway-level alignment was substantially stronger than directional concordance at the individual-gene level.

### 3.6. Redundancy-Aware Reconstruction Identifies 35 Directionally Concordant Programs

To minimize repeated counting of overlapping and hierarchically related annotations, the 91 gene sets that were significant in both discovery cohorts were consolidated into broader biological programs. Under the prespecified reference configuration—overlap coefficient, average linkage, and a similarity threshold of 0.50—the 91 gene sets resolved into 50 programs.

Thirty-five of these 50 programs were directionally concordant between sleep loss and atherosclerosis, corresponding to 70.0% agreement. Twenty-two programs were positively enriched in both cohorts, whereas 13 were negatively enriched in both. The program bootstrap 95% CI for the concordant proportion was 0.560–0.820. Under the reference specification, no program contained a mixture of concordant and discordant constituent gene sets: the 35 concordant programs comprised all 71 concordant gene sets, whereas the remaining 15 programs comprised the 20 discordant gene sets (Tables S16 and S17).

Redundancy reduction lowered the apparent concordance from 78.0% at the individual gene set level to 70.0% at the program level and correspondingly attenuated the nominal binomial evidence. This reduction was biologically and statistically plausible because closely related concordant annotations were often represented by multiple overlapping gene sets. The program-level estimate was therefore treated as the principal summary of cross-compartment convergence, whereas the gene set level estimate was retained as a secondary, non-independent analysis. The stability of this interpretation across alternative clustering specifications is summarized in Table S18.

The positively concordant programs were dominated by neutrophil degranulation; specific, tertiary, and azurophil granule compartments; myeloid leukocyte activation; respiratory burst; antimicrobial humoral responses; antioxidant processes; and lipid localization. In contrast, the negatively concordant programs were characterized by RNA splicing, spliceosomal organization, chromatin organization, epigenetic regulation, and cilium assembly. Several annotations with glial or neuroinflammatory labels clustered within the myeloid program structure because of substantial gene sharing between myeloid and microglial gene sets. These terms were therefore interpreted as annotation overlap rather than as evidence of direct central nervous system involvement. Overall, the reconstructed program architecture indicated coordinated enrichment of effector innate immune processes together with relative depletion of RNA processing, chromatin regulating, and related cellular maintenance programs across the circulating and vascular compartments. These findings remain computational and do not establish that the same cellular populations or causal regulatory mechanisms operate in both conditions.

### 3.7. Directional Generalization in a Distinct Acute Sleep-Deprivation Cohort

The fixed set of 35 concordant programs defined in the discovery analysis was transferred without modification to GSE56931, a biologically related but methodologically distinct cohort examining acute total sleep deprivation rather than controlled sleep restriction. All 35 programs could be scored in this dataset.

Thirty-two of the 35 programs changed in the direction predicted by the discovery analysis, corresponding to 91.4% directional concordance. Given the marginal sign distribution observed in GSE56931, the expected concordance under the marginal null was 0.540; the corresponding exact binomial *p* value was 2.0 × 10^−6^. The program bootstrap 95% CI for the concordant proportion was 0.800–1.000 (Table S19). Nine programs met the BH FDR threshold in GSE56931, and eight changed in the discovery consistent direction. The concordant signals included increased activity of neutrophil degranulation, granule-associated, and myeloid leukocyte activation programs, together with reduced activity of RNA splicing, spliceosomal, and chromatin regulatory programs. One program characterized by tubulin-binding annotations met the FDR threshold in the primary analysis in the direction opposite to its discovery-defined direction. The same directional reversal persisted in the matched-clock sensitivity analysis, although it was not FDR-significant under that specification; the result was therefore retained as a persistent discordant finding rather than excluded post hoc.

In the matched-clock sensitivity analysis, the sleep-deprivation phase was restricted to the first 24 h to align its clock-hour coverage more closely with that of the baseline observations. Under this specification, 28 of 35 programs remained directionally concordant, corresponding to 80.0% agreement (exact binomial *p* = 4.1 × 10^−4^). Program-level effect estimates were strongly correlated between the primary and matched-clock analyses (Spearman ρ = 0.97; Table S20).

These findings support directional generalization of the discovery-defined program architecture across two distinct experimental paradigms of sleep loss. They should not be interpreted as replication of effect magnitude or statistical significance, because the exposure design, sampling structure, covariate adjustment, and participant population differed between the two cohorts.

### 3.8. Program-Level Convergence Shows Limited Individual-Sample Separability

The preceding analyses addressed directional concordance of transcriptional programs at the group level. A separate, prespecified secondary analysis was therefore undertaken to determine whether these program-level signals contained sufficient information to distinguish individual sleep restriction samples from sleep extension samples while preserving the repeated measures structure of the cohort.

In the prespecified reference separability analysis, the 35 redundancy-collapsed program scores were entered into a LASSO logistic regression model evaluated by participant-grouped cross-validation. The reference-repeat area AUC was 0.558, and the mean AUC across five repeated grouped cross-validation runs was 0.537. The participant cluster bootstrap 95% CI for the reference-repeat AUC was 0.486–0.630 and included 0.5. Thus, the observed discrimination was not statistically distinguishable from chance under the bootstrap uncertainty analysis. This result was not interpreted as evidence of equivalence or as proof that the two experimental conditions were intrinsically inseparable. The same prespecified modeling pipeline was compared with an empirical null distribution generated by 1000 participant-blocked label permutations, yielding a one-sided empirical *p* value of 0.052. Although the observed AUC lay near the upper tail of the permutation distribution, it did not meet the prespecified α = 0.05 threshold. The bootstrap and permutation procedures addressed complementary inferential questions, but neither provided conventional statistical support for robust individual-sample separability (Table 4, Tables S21 and S22).

Across the remaining prespecified feature–model combinations, discrimination was similarly limited. Reference-repeat AUCs ranged from 0.474 to 0.575, and the majority of participant cluster bootstrap confidence intervals included 0.5. The shared leading-edge gene representation did not improve discrimination, and the fold-wise GSVA sensitivity analysis did not materially alter the overall interpretation. Complete results for all nine feature–model combinations and sensitivity analyses are provided in Tables S21 and S22. Taken together, these findings indicate that the transcriptomic programs showed reproducible directional convergence at the group level but did not provide consistent evidence of a robust classifier at the level of individual samples.

### 3.9. Hallmark Pathway Activity in Paired Carotid Plaque and Intact Arterial Tissue

**Figure 4:**
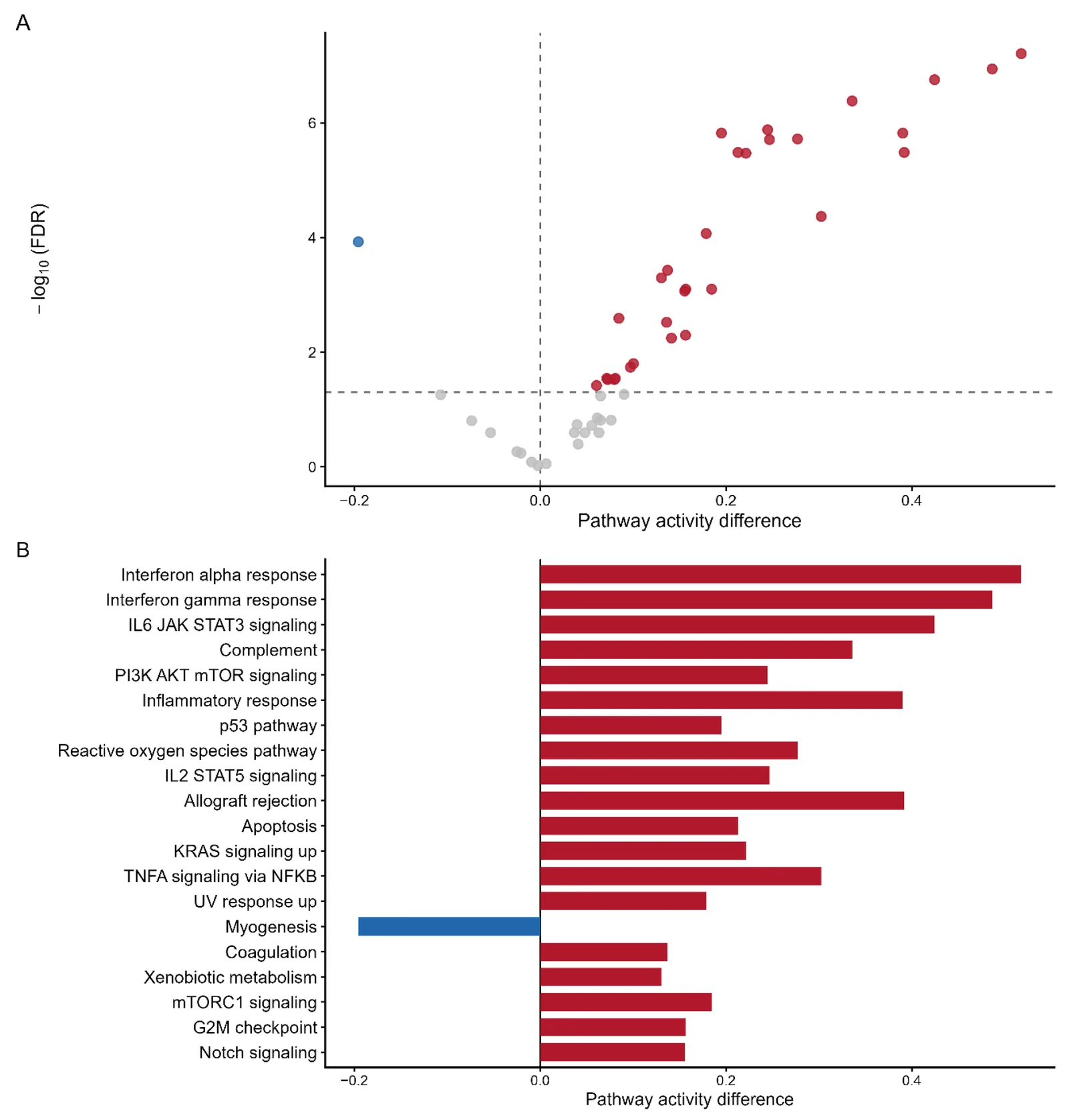
Hallmark pathway activity in paired carotid plaque and macroscopically intact arterial tissue from the GSE43292 cohort. (**A**) Each point represents one of the 50 MSigDB Hallmark gene sets. The *x*-axis shows the estimated paired difference in pathway activity, with positive values indicating higher activity in plaque, and the *y*-axis shows the corresponding −log_10_ BH-adjusted *p* value. The vertical dashed line marks zero pathway activity difference, and the horizontal dashed line marks the BH-adjusted *p* = 0.05 threshold. Red points denote pathways with significantly higher activity in plaque; the blue point denotes myogenesis, the only pathway with significantly higher activity in paired intact tissue; and grey points denote pathways that did not meet the FDR threshold. (**B**) The 20 Hallmark pathways with the smallest adjusted *p* values, ranked by statistical significance. Bar length represents the estimated paired difference in activity and bar color indicates the direction of the association, with red denoting higher activity in plaque and blue denoting higher activity in paired intact tissue. These results describe coordinated pathway-level differences accompanying plaque tissue and should not be interpreted as evidence of causality, diagnostic performance, prognosis, or therapeutic efficacy.

Hallmark pathway activity was examined in the GSE43292 atherosclerosis discovery cohort by applying GSVA to the 50 MSigDB Hallmark gene sets. Sample-level scores were compared between carotid plaque and paired macroscopically intact arterial tissue using a patient-paired empirical Bayes-moderated linear model. Thirty-one Hallmark pathways differed significantly after BH correction. Of these, 30 showed higher inferred activity in plaque, whereas only one pathway, myogenesis, showed higher activity in paired intact arterial tissue (Figure 4; Table S23).

The plaque-associated pattern was dominated by inflammatory, immune, stress-response, metabolic, and proliferative programs, consistent with the extensive transcriptional remodeling observed in the ranked transcriptome analysis.

### 3.10. Weighted Gene Co-Expression Network Architecture in the Sleep Loss Discovery Cohort

**Figure 5:**
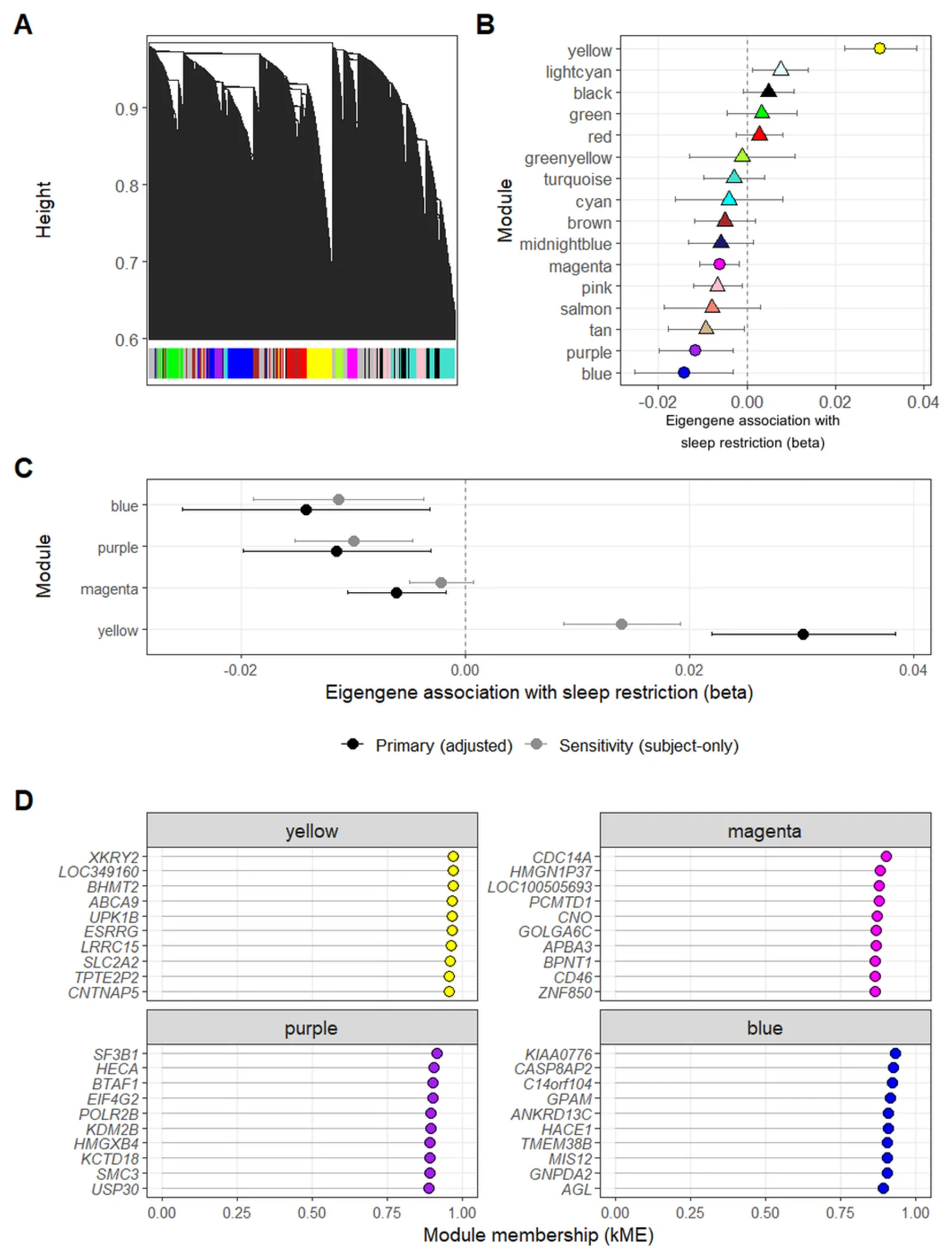
WGCNA of the GSE39445 whole-blood discovery cohort. (**A**) Gene-clustering dendrogram for the 14,655 genes retained after variance filtering and analyzed in a single signed network block. The color strip beneath the dendrogram indicates the final module assignments; grey denotes genes not assigned to a non-grey module. The remaining colors are WGCNA module labels and do not themselves imply biological meaning; the same module colors are used consistently across panels. (**B**) Primary mixed-effects model estimates for the association between each of the sixteen non-grey module eigengenes and sleep restriction relative to sleep extension. Points indicate regression coefficients (β), and horizontal lines indicate 95% CIs. Positive coefficients indicate higher eigengene expression during sleep restriction, whereas negative coefficients indicate higher eigengene expression during sleep extension. Circles denote modules meeting the BH FDR threshold, and triangles denote modules that did not meet the threshold. (**C**) Comparison of the primary covariate-adjusted estimates with the subject-only sensitivity estimates for the four sleep-associated modules. Black symbols represent the primary model and grey symbols represent the sensitivity model. (**D**) Ten genes with the highest signed module-membership values (kME) in each sleep-associated module. Genes are ranked within modules by descending own-module kME. The yellow module is shown for completeness but was excluded from biological interpretation because its low-expression, tissue-restricted gene composition was more consistent with technical signal than with a coherent whole-blood program.

WGCNA of the 14,655 genes in the upper 75% of expression variance in the GSE39445 whole-blood discovery cohort identified sixteen non-grey co-expression modules at a soft-thresholding power of 7. A total of 2732 genes (18.6%) were not assigned to a non-grey module and were retained in the grey module (Figure 5A; Table S24). Non-grey module sizes ranged from 76 genes in the lightcyan module to 1922 genes in the turquoise module. The largest non-grey modules were turquoise (1922 genes), blue (1757 genes), brown (1297 genes), and yellow (1277 genes) (Figure S4; Table S24). The signed network was constructed as a single block, and the gene dendrogram showed module assignments distributed across distinct hierarchical branches (Figure 5A).

### 3.11. Sleep-Associated Modules and Intramodular Candidate-Gene Prioritization

Relating module eigengenes to the sleep restriction contrast identified four non-grey modules that met the BH FDR threshold (Figure 5B; Tables S25 and S26). Three modules showed lower eigengene expression during sleep restriction and were therefore relatively higher during sleep extension: blue (β = −0.01419, FDR = 0.0485), purple (β = −0.01147, FDR = 0.0388), and magenta (β = −0.00614, FDR = 0.0388). The yellow module showed the strongest association and was higher during sleep restriction (β = +0.03014, FDR = 3.57 × 10^−11^). All four modules retained the same effect direction in the subject-only sensitivity analysis. The yellow, purple, and blue associations remained statistically significant, whereas the magenta association was attenuated and no longer met the FDR threshold, indicating that its statistical support depended more strongly on adjustment for hours awake and circadian phase (Figure 5C and Figure S5; Table S26).

Despite its strong statistical association, the yellow module was considered more consistent with technical than biological signal and was therefore excluded from biological interpretation. Its constituent genes had the lowest median expression among the detected modules, below that of the unassigned grey genes, and its highest-membership genes were dominated by tissue-restricted, non-hematopoietic transcripts, including *XKRY2*, *BHMT2*, *UPK1B*, and *SLC2A2*, which are not expected to be meaningfully expressed in whole blood. Together, the low expression level and atypical tissue identity of these genes were consistent with a detection-floor probe cluster rather than a coherent blood-expressed program. The yellow module was nevertheless retained in the complete module-level and intramodular results for transparency (Figure 5D; Tables S26–S29).

Among the remaining sleep-extension-associated modules, the purple and blue modules showed the clearest biologically coherent intramodular structure. The purple module contained high-membership genes involved in transcriptional and RNA-processing functions, including *SF3B1*, *POLR2B*, *EIF4G2*, and *SMC3*. The blue module contained high-membership genes related to cellular metabolism and broadly expressed housekeeping processes, including *GPAM*, *GNPDA2*, and *AGL*. The more weakly and covariate-dependently associated magenta module contained high-membership genes such as *CDC14A* and *CD46* (Figure 5D and Figure S6; Tables S28 and S29). These findings nominate transcriptional, RNA-processing, and metabolic co-expression programs as exploratory candidates contributing to the whole-blood response to sleep loss.

### 3.12. Immune- and Stromal-Cell-Associated Transcriptional Signals

In the GSE39445 whole-blood cohort, MCP-counter identified four of the ten evaluated populations as significantly associated with sleep condition after BH correction (Figure S7; Table S30). Fibroblast-associated scores were higher during sleep restriction than during sleep extension (β = 0.407 SD, FDR = 0.0091), whereas endothelial-cell-associated (β = −0.337 SD, FDR = 0.0032), B-lineage-associated (β = −0.197 SD, FDR = 0.0120), and cytotoxic-lymphocyte-associated scores (β = −0.230 SD, FDR = 0.0339) were lower during sleep restriction. The prespecified marker score analysis identified three FDR-significant populations in the same cohort (Figure S7; Table S31). Fibroblast-associated (β = 0.373 SD, FDR = 0.0082) and neutrophil-associated scores (β = 0.267 SD, FDR = 0.0260) were higher during sleep restriction, whereas the T-cell-associated score was lower (β = −0.205 SD, FDR = 0.0348).

All four MCP-counter findings meeting the primary-model FDR threshold retained the same coefficient direction in the reduced sensitivity analysis. Across all ten populations, seven showed concordant coefficient directions between MCP-counter and the prespecified marker score approach; fibroblast-associated scores met the FDR threshold under both methods, and six populations met the threshold under at least one method (Table S32). In the patient-paired GSE43292 analysis, all ten populations showed positive plaque-associated coefficients under both scoring approaches, and the coefficient direction was concordant between methods for every population (Figure S7; Tables S33 and S34). Nine populations met the FDR threshold under MCP-counter, with CD8 T cells as the only exception (β = 0.424 SD, FDR = 0.106). Nine populations also met the threshold under the prespecified marker score approach, with fibroblasts as the only exception (β = 0.181 SD, FDR = 0.367); eight populations were therefore significant under both methods. The direction of every plaque-associated effect was preserved in the corresponding unpaired sensitivity analysis under both scoring approaches. Population-level comparison confirmed concordant coefficient directions between MCP-counter and the prespecified marker score approach for all ten populations, with eight populations meeting the FDR threshold under both methods (Table S35).

Cross-cohort integration revealed a substantially more selective pattern than the broad plaque-associated changes observed within GSE43292 (Figure S8A; Table 5 and Table S36).

MCP-counter coefficients were directionally concordant between GSE39445 and GSE43292 for fibroblasts, neutrophils, and CD8 T cells, whereas marker score coefficients were concordant for fibroblasts, neutrophils, endothelial cells, and monocytic lineage. Only fibroblast- and neutrophil-associated scores satisfied the prespecified four-layer directional concordance criterion, requiring all four cohort-by-method estimates to share the same sign. At the sample level, MCP-counter and prespecified marker scores were positively rank-correlated for all ten populations in GSE43292 (Figure S8B). Spearman correlation coefficients ranged from 0.712 for CD8 T cells to 0.964 for monocytic lineage, and all remained significant after BH correction.

Neutrophil-associated scores were higher during sleep restriction under the marker score approach (β = 0.267 SD, FDR = 0.0260) and showed the same positive direction under MCP-counter, although the latter estimate did not independently meet the FDR threshold (β = 0.151 SD, FDR = 0.1417). In carotid plaque, neutrophil-associated scores were strongly increased under both MCP-counter (β = 0.776 SD, FDR = 4.71 × 10^−5^) and the marker score approach (β = 1.046 SD, FDR = 7.64 × 10^−6^). Fibroblast-associated scores were also higher during sleep restriction under both MCP-counter (β = 0.407 SD, FDR = 0.0091) and the marker score approach (β = 0.373 SD, FDR = 0.0082). The corresponding plaque-associated estimates were positive under both methods, although statistical support differed: the MCP-counter estimate met the FDR threshold (β = 0.598 SD, FDR = 0.0017), whereas the marker score estimate did not (β = 0.181 SD, FDR = 0.367). The fibroblast-associated result therefore showed consistent directional convergence without statistical significance in every constituent analysis. In whole blood, this finding was interpreted as a fibroblast- and extracellular-matrix-marker-associated transcriptional signature rather than as evidence of circulating fibroblast abundance.

Taken together, neutrophil-associated transcriptional activity constituted the stronger shared signal across the systemic sleep restriction and plaque compartments, whereas the fibroblast-associated finding represented a secondary stromal and extracellular-matrix-related signature. The remaining populations were retained in the complete cohort-specific and integrated results but did not satisfy the full four-layer directional concordance criterion. These findings support a restricted pattern of cross-compartment convergence rather than a general correspondence between sleep loss-associated whole-blood signals and the immune–stromal transcriptional architecture of atherosclerotic plaque.

### 3.13. TF Target Regulatory Signatures Associated with the Shared Leading-Edge Program

Over-representation analysis of the primary 830-gene shared leading-edge input identified seven regulatory context signals that remained significant after resource-specific BH correction. Within the curated CollecTRI network, two regulons were enriched: *CEBPE* (fold enrichment, 4.68; FDR = 0.00569) and *SPI1* (fold enrichment, 1.82; FDR = 0.0369). Five additional findings were detected in the MSigDB C3:TFT:GTRD collection and were therefore interpreted as GTRD-derived regulatory target signatures rather than as direct evidence of transcription factor activity: *ZNF581* (FDR = 0.000828), *SRPK1* (FDR = 0.00175), *PSMB5* (FDR = 0.0308), *ZSCAN21* (FDR = 0.0308), and *TAF9B* (FDR = 0.0345). No target signature from the MSigDB C3:TFT legacy collection met the prespecified FDR threshold in the primary analysis. Complete resource-specific results are provided in Table S37, whereas the seven FDR-significant primary findings are summarized in Table S38 and visualized in Figure S9.

Of the 830 primary genes, 417 mapped to the frozen human CollecTRI network and 797 mapped to the GTRD collection. Accordingly, overlap counts and enrichment estimates were interpreted within their respective resource-specific backgrounds and were not compared directly across databases. Figure S9 therefore presents the two CollecTRI regulons and the five GTRD-derived signatures in separate panels and includes only findings meeting the prespecified FDR threshold of 0.05.

Sensitivity analysis across the two additional prespecified input definitions showed that *CEBPE*, *ZNF581*, and the *SRPK1*-associated target signature remained significant in all three analyses. SPI1, *PSMB5*, *ZSCAN21*, and *TAF9B* retained significance in two of the three input definitions, indicating partial sensitivity to the definition of the leading-edge gene set. These stability results are detailed in Table S39. None of the seven primary findings achieved FDR-significant support in more than one independent TF target resource.

Taken together, the results support a source-specific TF target regulatory context within the shared leading-edge program. They do not establish direct TF binding, TF activation or inhibition, causal regulation, or therapeutic relevance. In particular, the GTRD findings should be interpreted as enriched regulatory target signatures rather than as evidence that the corresponding named factors were functionally active.

### 3.14. STRING Network Architecture and Functional Enrichment

**Figure 6:**
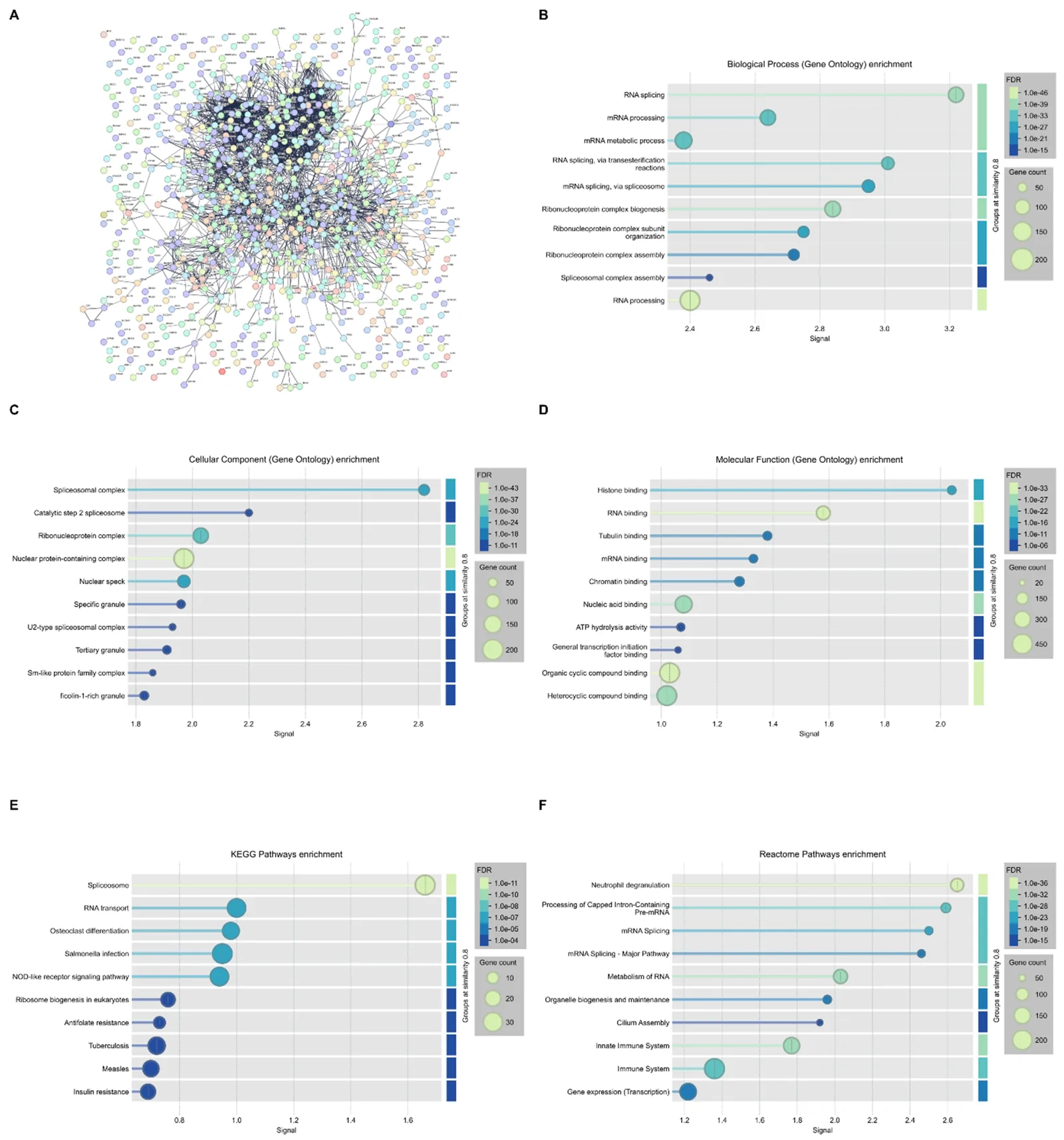
STRING PPI network and category-specific functional enrichment of the prespecified shared leading-edge gene set. (**A**) PPI network generated using STRING version 12.0 from 830 submitted human gene symbols, of which 828 were mapped to distinct protein nodes. Nodes represent mapped proteins, whereas edges represent known or predicted functional associations. Node colors are used solely to facilitate visual discrimination and do not encode expression direction, functional class, network centrality, disease relevance, or statistical significance. (**B**) GO-BP enrichment. (**C**) GO-CC enrichment. (**D**) GO-MF enrichment. (**E**) KEGG pathway enrichment. (**F**) Reactome pathway enrichment. In panels (**B**–**F**), the ten terms with the highest STRING signal within each annotation category are displayed after STRING term grouping at a similarity threshold of 0.8. The *x*-axis represents STRING signal, point size represents the number of mapped proteins associated with each term, and point color represents the BH FDR. Because the input gene set was not partitioned according to enrichment direction, neither node nor term appearance denotes upregulation or downregulation. The enrichment panels describe the functional annotation landscape of the mapped network and should not be interpreted as evidence of direct pathway activation, independent confirmation of cross-condition convergence, causal involvement, disease specificity, or therapeutic actionability.

Of the 830 gene symbols in the locked shared leading-edge set (Table S40), 828 were mapped to distinct STRING protein nodes. *GGT3P* and *HSP90AB3P* were not represented as protein nodes. At the prespecified high-confidence interaction threshold, the network contained 2755 observed edges, compared with 1772 expected edges for a random protein set of comparable size. The STRING web interface reported an average node degree of 6.65, an average local clustering coefficient of 0.433, and a PPI enrichment *p* value of <1.0 × 10^−16^, corresponding to the lower reporting bound of the STRING interface. These findings indicated that the mapped proteins formed a more cohesive functional network than would be expected by chance. They did not, however, establish direct physical interaction for every edge, causal importance, disease specificity, or therapeutic relevance (Figure 6A; Table 6). The PPI enrichment statistic was therefore interpreted as a measure of network cohesion rather than as independent evidence of cross-condition convergence.

Functional enrichment identified a prominent innate immune and myeloid effector component. Neutrophil degranulation was among the most strongly enriched reactome pathways, with 105 of 476 annotated genes represented in the network (FDR = 2.98 × 10^−36^). Related annotations included the innate immune system, secretory and specific granule compartments, regulation of inflammatory response, regulation of phagocytosis, respiratory burst, and the NOD-like receptor signaling pathway. Regulation of inflammatory response included 64 of 371 annotated genes (FDR = 4.62 × 10^−17^), whereas the NOD-like receptor signaling pathway included 27 of 173 annotated genes (FDR = 3.23 × 10^−6^). Collectively, these findings were consistent with the myeloid, neutrophil granule, and inflammatory programs identified in the preceding directionally concordant gene set analysis. Category-specific STRING enrichment landscapes are shown in Figure 6B–F, and representative terms are summarized in Table 6.

A second major component involved RNA-processing and chromatin-regulatory machinery. RNA splicing had the highest STRING signal among GO-BP terms, with 101 of 370 annotated genes represented in the network (signal = 3.22; FDR = 7.48 × 10^−42^). Ribonucleoprotein complex biogenesis was also strongly enriched, with 106 of 449 annotated genes represented (FDR = 3.77 × 10^−39^). Related annotations included ribosome biogenesis, spliceosomal and ribonucleoprotein complex organization, nuclear speck, the KEGG spliceosome pathway, and reactome mRNA-splicing pathways. Chromatin-associated enrichment was represented by chromatin organization (91/612; FDR = 9.78 × 10^−21^) and histone binding (55/252; FDR = 4.95 × 10^−18^), together with related histone-modifying and nucleosome-associated annotations.

Lipid-handling processes were also represented, although their enrichment was more modest than that of the immune and RNA-processing components. These included lipid localization (37/365; FDR = 1.3 × 10^−4^), sterol transport (13/82; FDR = 3.0 × 10^−3^), and regulation of cholesterol efflux (8/37; FDR = 8.5 × 10^−3^). Additional annotations involved the ciliary and microtubule apparatus, including cilium assembly (45/200; FDR = 2.24 × 10^−15^) and tubulin binding (57/380; FDR = 2.60 × 10^−12^), together with the reactome circadian clock term (14/68; FDR = 2.7 × 10^−4^) and the KEGG insulin-resistance pathway (17/106; FDR = 3.4 × 10^−4^). These findings were regarded as contextual components of the shared leading-edge network rather than as evidence of newly established disease mechanisms.

Overall, the STRING enrichment results described the functional composition of the shared leading-edge set rather than providing independent validation of cross-condition convergence. No individual annotation was considered evidence of direct pathway activation or causal involvement.

### 3.15. Cytoscape Network Topology and Topology-Supported Candidate Genes

**Figure 7:**
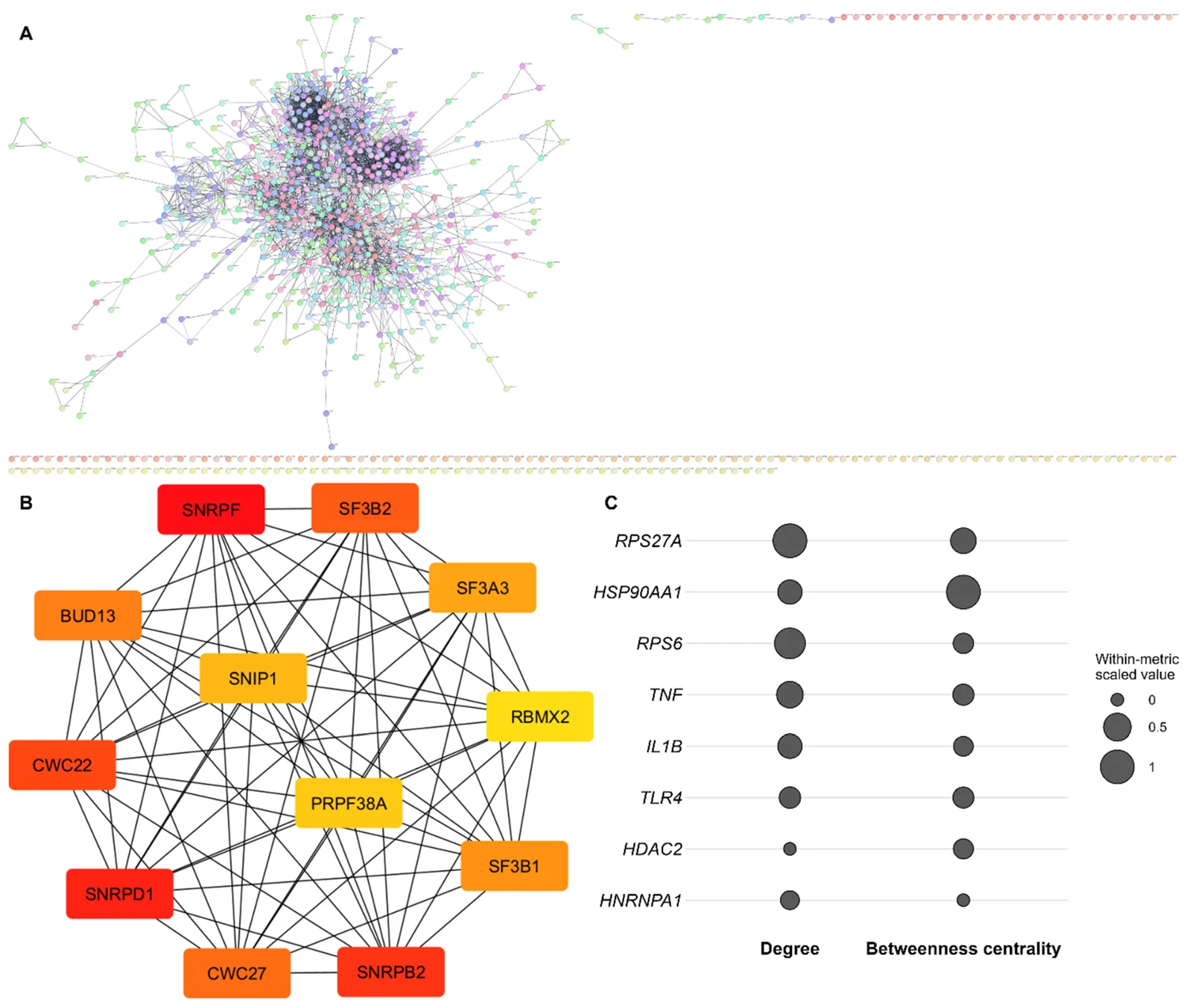
Cytoscape topology of the shared sleep loss–atherosclerosis transcriptomic program. (**A**) Full STRING-derived protein–protein interaction network after import into Cytoscape. The network was treated as undirected. Node colors were retained from the imported visualization solely to improve visual discrimination and do not encode expression direction, functional class, centrality, disease relevance, or statistical significance. Isolated nodes and disconnected components were retained. (**B**) Subnetwork formed by the 12 proteins with the highest MCC scores identified using cytoHubba. These proteins constituted a densely interconnected RNA-processing and spliceosomal group. The red-to-yellow node gradient represents relative MCC ranking within the displayed subnetwork. Because MCC scores differed only minimally across these proteins, neither the color gradient nor the internal ordering was interpreted as evidence of a meaningful biological hierarchy. (**C**) Proteins shared between the 20 nodes retained in the Cytoscape export after ranking by descending degree in the complete 828-node mapped network and the 20 highest-ranked nodes by betweenness centrality within the 626-node largest connected component. Under this tie-handling rule, the intersection comprised HSP90AA1, RPS27A, TNF, TLR4, RPS6, HDAC2, IL1B, and HNRNPA1. Degree and betweenness centrality were scaled independently from 0 to 1 across these eight proteins for visualization. Dot size represents the within-metric scaled value, whereas the uniform gray fill carries no additional information. Unscaled values and metric-specific rankings are provided in Table S42 and the accompanying figure source data file. The tie-handling rule was used only to define the descriptive display and does not imply a biologically meaningful distinction among proteins with equal degree. Network position does not establish causal importance, disease specificity, direct regulation, or therapeutic efficacy.

Import of the STRING-derived network into Cytoscape provided a complementary node-level assessment of the 828 mapped proteins. The network comprised 196 connected components, including 189 isolated nodes, and the largest connected component contained 626 proteins. Degree was evaluated across the complete mapped network, whereas betweenness and closeness centrality were assessed within the 626-node largest connected component. NetworkAnalyzer produced distinct rankings across these topological measures, while cytoHubba MCC preferentially identified proteins embedded within densely interconnected clique structures (Figure 7; Table 7). Complete node-level results are provided in Table S42.

The 12 highest MCC-scoring proteins were SNRPF, SNRPD1, SNRPB2, CWC22, SF3B2, CWC27, BUD13, SF3B1, SF3A3, SNIP1, PRPF38A, and RBMX2. These proteins formed a compact RNA-processing and spliceosomal subnetwork, consistent with the strong enrichment of RNA splicing and ribonucleoprotein-complex biogenesis observed in the STRING analysis. MCC scores differed only minimally across this highest-scoring group; therefore, their internal ordering was not interpreted as a meaningful biological hierarchy. To identify proteins supported jointly by connectivity and network-bridging position, the 20 nodes retained in the Cytoscape export after ranking by descending full-network degree were intersected with the 20 highest-ranked nodes by betweenness centrality within the largest connected component. A four-way tie at degree 32 spanned positions 19–22, and the Cytoscape export order retained HDAC2 within the degree top 20. Using this export-order tie-handling rule, eight proteins were shared between the two rankings: HSP90AA1, RPS27A, TNF, TLR4, RPS6, HDAC2, IL1B, and HNRNPA1 (Figure 7C).

This dual-metric group linked the RNA-processing architecture emphasized by MCC with inflammatory, stress-responsive, epigenetic, translational, and RNA-regulatory features of the shared transcriptomic program. *TNF*, *TLR4*, and *IL1B* provided the clearest topological connection to the inflammatory component relevant to sleep loss and human atherosclerosis, whereas *HSP90AA1* and *HDAC2* extended this pattern toward cellular stress responses and chromatin-associated regulation. *RPS27A* and *RPS6* represented translational machinery, while *HNRNPA1* represented RNA processing.

### 3.16. Single-Cell Transcriptomic Localization and Cell-Type-Specific Distribution of Candidate Genes and Transcriptional Programs

**Figure 8:**
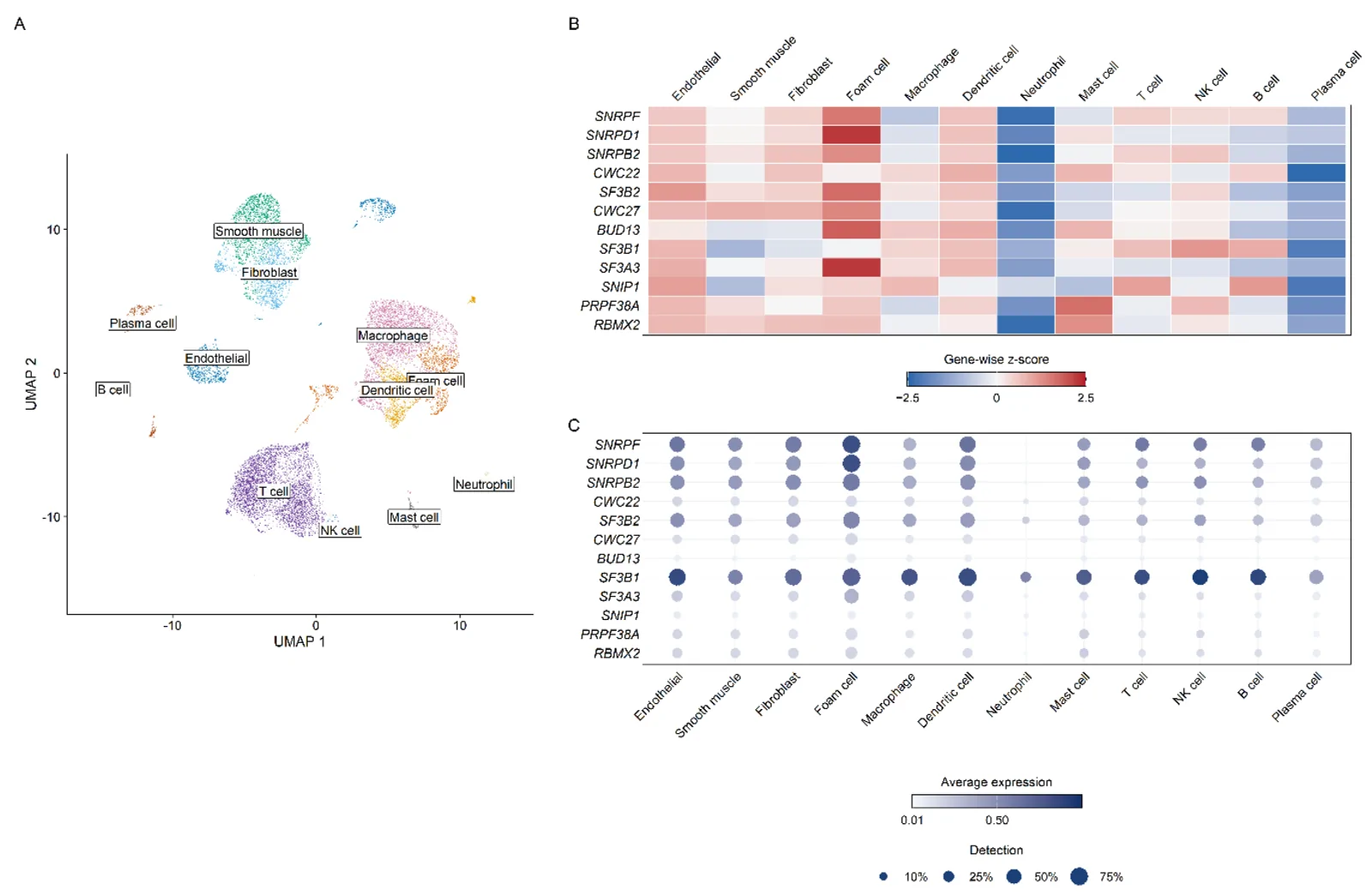
Single-cell localization of the prespecified network-prioritized candidates shared across sleep-related and atherosclerotic transcriptomic analyses. (**A**) Marker-primary annotation of 49,188 cells from the human atherosclerotic scRNA-seq dataset GSE253903, visualized in the RPCA-integrated UMAP space. Cell identities were assigned using the prespecified marker-based framework, with independent SingleR classifications serving as an orthogonal concordance layer. (**B**) Cell-type localization of the 12 prespecified MCC-supported candidates across the same marker-primary populations. Values represent gene-wise standardized average log-normalized expression; positive and negative z-scores indicate relative enrichment and relative depletion, respectively, across cell types for each candidate. Candidate identity and display order were established by the preceding network analysis. (**C**) Average log-normalized expression and detection frequency of the same candidates across the corresponding cell types. Point color represents average expression, whereas point area represents the proportion of cells with detectable transcript expression. For visualization only, point-size scaling was capped at 80% detection; the original detection percentages were retained unchanged in the source data. Joint consideration of relative cell-type enrichment, unstandardized average expression, and detection frequency distinguishes compartment-selective expression maxima from broadly distributed transcript detection and provides a higher-resolution cellular framework for interpreting the network-prioritized sleep–atherosclerosis candidates. The resulting patterns represent cellular contextualization rather than independent pharmacological validation, evidence of causality, or confirmation of therapeutic efficacy.

The final GSE253903 object comprised 49,188 cells from 12 human carotid plaque specimens. Marker-primary annotation resolved 12 vascular, stromal, myeloid, and lymphoid populations in the RPCA-integrated UMAP representation (Figure 8A), whereas the underlying unsupervised structure comprised 19 Leiden clusters (Figure S10). Cluster-level marker-primary assignments, second-ranked marker groups, marker score margins, reference-derived classifications, broad-label mappings, concordance indicators, independent support counts, and annotation status are reported in Table S43. SingleR classifications were concordant with the marker-primary assignment for both reference collections in seven clusters and for one reference collection in three additional clusters. Neither reference yielded a concordant broad label for the remaining nine clusters, whose final identities remained grounded in the marker-primary framework (Figure S11). T cells constituted the largest cellular compartment (13,502 cells; 27.5%), followed by macrophages (10,384 cells; 21.1%) and smooth muscle cells (6469 cells; 13.2%). Together, these three populations accounted for 61.7% of the retained cells. The complete cluster-level marker-primary cell-type assignments and corresponding cell abundances are reported in Table S44.

Of the 830 shared leading-edge genes carried forward from the cross-cohort analysis, 822 (99.0%) were represented in the final single-cell expression matrix. *CST11*, *DEFB114*, *GGT3P*, *HSP90AB3P*, *RNASE8*, *SLC2A2*, *SPAG11B*, and *UTP14C* were not measured and consequently had no cellular localization estimates. Endothelial cells represented the predominant average-expression localization for 203 measured genes, whereas foam cells represented the predominant localization for 176 genes. Together, these two compartments accounted for 379 of the 822 measured genes (46.1%). All 12 marker-primary cell types were represented among the predominant gene localizations, indicating that the shared leading-edge architecture extended across multiple vascular, stromal, myeloid, and lymphoid compartments (Table S45).

All 12 MCC-supported candidates were represented in GSE253903. Seven candidates—*SNRPF*, *SNRPD1*, *SNRPB2*, *SF3B2*, *CWC27*, *BUD13*, and *SF3A3*—showed their highest average expression in foam cells. The remaining maxima occurred in dendritic cells for *CWC22*, NK cells for *SF3B1*, B cells for *SNIP1*, and mast cells for *PRPF38A* and *RBMX2* (Figure 8B; Table S46). The expression–detection representation further demonstrated that the cell type with the highest average expression did not invariably show the greatest detection frequency. In foam cells, *SNRPF*, *SNRPD1*, *SNRPB2*, and *SF3B2* combined their highest average expression with detection in 75.6%, 74.4%, 68.1%, and 66.2% of cells, respectively. In contrast, *SF3B1* reached its highest average expression in NK cells, where it was detected in 56.8% of cells, but showed broader detection in dendritic cells (76.3%) and foam cells (75.7%). Similarly, *PRPF38A* and *RBMX2* reached their highest average expression in mast cells, whereas their detection frequencies were greater in foam cells than in mast cells (*PRPF38A*, 29.0% versus 19.0%; *RBMX2*, 30.9% versus 17.4%). Joint consideration of relative enrichment, expression magnitude, and detection breadth therefore distinguished compartment-selective expression maxima from broadly distributed transcript detection (Figure 8C).

Cluster-resolved analysis provided additional resolution below the marker-primary cell-type categories. Candidate-expression profiles varied across the 19 Leiden clusters, including among clusters assigned to the same broader cellular identity. This subpopulation-level variation refined the localization patterns observed in the cell-type-level analysis without altering the candidate set or its network-derived order (Figure S12).

The 35 directionally concordant transcriptional programs comprised 22 concordant-up and 13 concordant-down programs. The proportion of unique program genes represented in GSE253903 ranged from 61.3% to 100.0%, with a median coverage of 90.8%. Among the concordant-up programs, the highest localization score occurred in foam cells for nine programs, dendritic cells for nine, endothelial cells for two, and fibroblasts for two. These programs encompassed neutrophil granule and degranulation signatures, myeloid activation, respiratory burst, inflammatory and antimicrobial responses, reactive oxygen species metabolism, and related transport processes. In contrast, all 13 concordant-down programs localized predominantly to either endothelial cells (seven programs) or foam cells (six programs). They encompassed ciliary organization, chromatin and epigenetic regulation, RNA splicing, ribosome biogenesis, nuclear ribonucleoprotein organization, tubulin binding, and protein–RNA complex organization. Complete program-level gene coverage and localization metrics are reported in Table S47, and the corresponding cell-type distributions are visualized in Figure S13.

The shared leading-edge genes extended across vascular, stromal, myeloid, and lymphoid compartments, whereas the network-prioritized candidates showed a prominent but nonexclusive foam cell localization. At the program level, concordant-up immune and myeloid effector signals localized principally to foam and dendritic cells, while concordant-down RNA-processing, chromatin-associated, and ciliary programs were concentrated in foam and endothelial compartments. The divergence between expression magnitude and detection breadth, together with the additional cluster-level resolution, provided a more discriminating cellular framework for interpreting the preceding bulk-transcriptomic and network findings. These results represent cellular contextualization rather than condition-level differential expression evidence or direct pharmacological validation.

### 3.17. Exact-Cis Genetic Support and Regional Colocalization of Network-Prioritized Candidates

**Figure 9:**
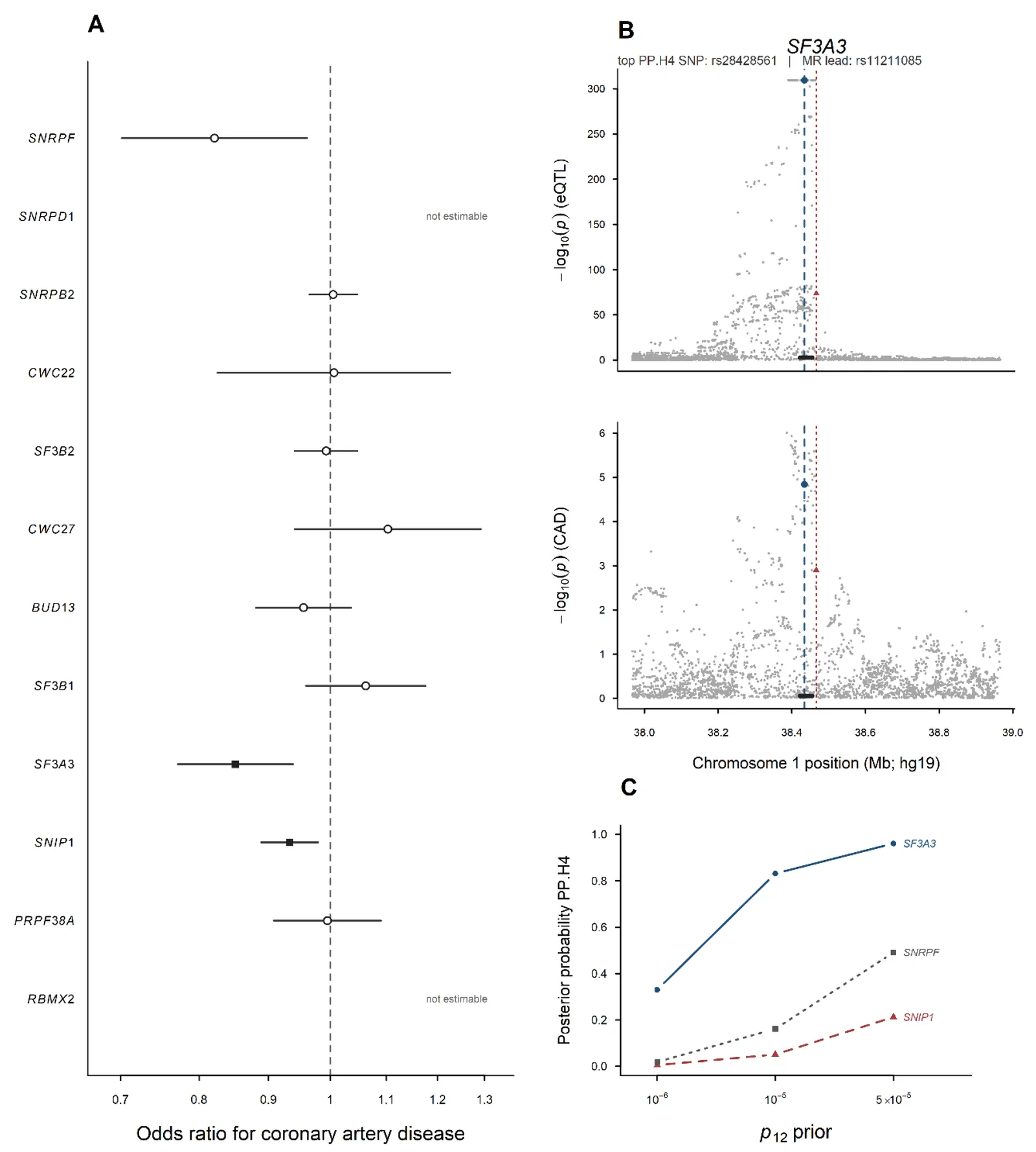
Human genetic support for network-prioritized candidate genes. (**A**) Primary exact-cis expression-instrument Mendelian randomization estimates for CAD across the 12 prespecified candidates. Wald ratios were used for single-instrument analyses, and inverse-variance weighting was used as the primary estimator for multi-instrument analyses. Filled squares denote estimates that remained significant after BH correction across all 12 candidates; open circles denote estimable results that did not meet the adjusted significance threshold. Candidates without an estimable exposure instrument are retained without an effect estimate. (**B**) Regional association profiles for *SF3A3* expression and CAD within the prespecified hg19 region. The blue dashed line marks rs28428561, the variant with the highest SNP-level PP.H4, and the red dotted line marks rs11211085, the lead Mendelian randomization instrument used to define the regional query. The eQTL and CAD tracks use independent *y*-axis scales. (**C**) Sensitivity of PP.H4 to the *p*_12_ prior for *SF3A3*, *SNIP1*, and *SNRPF*. The primary colocalization analysis used *p*_12_ = 1 × 10^−5^. These findings provide genetically informed support and do not establish causal validation, target validation, or therapeutic efficacy.

Across the 12 prespecified exposure datasets, 5219 genome-wide-significant associations satisfied the exact-cis criterion. Linkage-disequilibrium clumping and instrument-strength filtering retained 19 independent strong instruments, of which 18 remained after allele harmonization. The *SF3A3* variant rs28525112 was excluded because its C/G allele pair was palindromic and its exposure effect-allele frequency of 0.427 was close to the ambiguity threshold. Primary Mendelian randomization estimates were therefore available for 10 of the 12 candidates. No genome-wide-significant instrument was available in the prespecified exposure datasets for *SNRPD1* or *RBMX2*; both candidates were retained as non-estimable in the multiplicity framework (Figure 9A; Table 8 and Tables S48–S50). Two candidates remained significant after BH correction across all 12 prespecified candidates. For *SF3A3*, two harmonized exact-cis instruments yielded an inverse-variance-weighted OR of 0.851 per genetically predicted higher expression (95% CI, 0.772–0.939; *p* = 0.00125; BH-adjusted *p* = 0.0150). For SNIP1, four harmonized exact-cis instruments yielded an inverse-variance-weighted OR of 0.933 (95% CI, 0.889–0.979; *p* = 0.00510; BH-adjusted *p* = 0.0306). *SNRPF* showed a nominal single-instrument association (OR, 0.821; 95% CI, 0.701–0.961; *p* = 0.0143), but this association did not remain significant after multiplicity correction (BH-adjusted *p* = 0.0572). None of the remaining estimable candidates showed FDR-significant exact-cis genetic support. Regional colocalization further distinguished the genetically supported candidates. For *SF3A3*, analysis of 2726 complete shared regional SNPs yielded PP.H4 = 0.831 and PP.H3 = 0.154 under the primary shared-association prior of *p*_12_ = 1 × 10^−5^, with PP.H4/(PP.H3 + PP.H4) = 0.844. The highest SNP-level posterior probability (PP) was assigned to rs28428561 (SNP-level PP.H4 = 0.987).

Support for a shared signal was prior-sensitive: PP.H4 was 0.330 at *p*_12_ = 1 × 10^−6^ and 0.961 at *p*_12_ = 5 × 10^−5^. *SF3A3* therefore provided the strongest combined regional evidence, although the colocalization inference remained conditional on the single-causal-variant assumption and the prior specification (Figure 9B,C and Figure S14A; Tables S51 and S52).

For SNIP1, analysis of 2976 shared regional SNPs favored distinct eQTL and CAD signals under the primary prior (PP.H3 = 0.864; PP.H4 = 0.051; PP.H4/[PP.H3 + PP.H4] = 0.056). PP.H4 remained low at *p*_12_ = 1 × 10^−6^ (0.005) and increased to 0.212 at *p*_12_ = 5 × 10^−5^, without providing strong posterior support for a shared signal. For SNRPF, analysis of 3840 shared SNPs yielded PP.H4 = 0.162 under the primary prior. Although PP.H4/(PP.H3 + PP.H4) was 0.566, the low absolute PP.H4 supported an inconclusive interpretation (Figure S14B,C; Tables S51 and S52).

The two retained SF3A3 instruments produced directionally concordant Wald estimates. Statistical support was concentrated in rs11211085 (OR, 0.836; 95% CI, 0.749–0.932; *p* = 0.00125), whereas the estimate for rs12129777 was less precise (OR, 0.918; 95% CI, 0.734–1.149; *p* = 0.457). Both retained *SF3A3* instruments substantially exceeded the prespecified instrument strength threshold (rs11211085, F = 332.4; rs12129777, F = 43.7; Table S49, panel B). The inverse-variance-weighted estimate showed no evidence of heterogeneity (Cochran’s Q = 0.551; *p* = 0.458); however, with only two instruments, this test has limited capacity to detect heterogeneity. Weighted-median and MR-Egger estimates were not estimable under the prespecified minimum of three independent instruments. Neither retained SF3A3 instrument was palindromic, and exclusion of palindromic variants therefore left the primary estimate unchanged. Steiger analyses supported the exposure-to-outcome direction under all six combinations of assumed CAD prevalence and outcome sample-size definition (Figure S15A; Table S50). For SNIP1, the weighted-median estimate was directionally consistent with the primary result (OR, 0.925; 95% CI, 0.878–0.974; *p* = 0.00325). There was no evidence of heterogeneity in the primary inverse-variance-weighted analysis (Cochran’s Q = 2.073; *p* = 0.557) or of directional horizontal pleiotropy according to the MR-Egger intercept (intercept = 0.00829; *p* = 0.633). Leave-one-out analysis nevertheless showed that exclusion of rs114745536 attenuated the estimate toward the null (β = −0.0152; *p* = 0.755), whereas exclusion of each of the other three variants preserved the negative direction and nominal significance. The SNIP1 association was therefore classified as sensitive to a single variant. Exclusion of palindromic variants did not alter the primary estimate, and all six Steiger specifications supported the exposure-to-outcome direction (Figure S15B; Table S50).

Collectively, these findings identified SF3A3 as the principal candidate with convergent transcriptomic, network topological, exact-cis genetic, and regional evidence. SNIP1 retained FDR-significant Mendelian randomization support but was sensitive to a single variant and did not colocalize under the primary model, whereas SNRPF remained an exploratory nominal association. These findings provide genetically informed support and do not establish causal validation, target validation, or therapeutic efficacy.

### 3.18. Disease Enrichment of the Shared Leading-Edge Gene Set

Disease enrichment analysis of the 830 locked shared leading-edge genes identified 947 FDR-significant terms among 12,968 eligible terms tested across five disease-oriented libraries. Within the prespecified cardiovascular domains, significant enrichment was observed for atherosclerosis (92 of 830 query genes overlapping 1013 of 16,674 background genes; enrichment ratio, 1.8245; BH-adjusted *p* = 2.01 × 10^−6^), carotid atherosclerosis (17/830 versus 102/16,674; enrichment ratio, 3.3482; BH-adjusted *p* = 5.13 × 10^−4^), and atherosclerosis of the aorta (6/830 versus 29/16,674; enrichment ratio, 4.1564; BH-adjusted *p* = 0.0331).

The shared gene set was also enriched for CAD (81/830 versus 880/16,674; BH-adjusted *p* = 7.39 × 10^−6^), coronary heart disease (75/830 versus 816/16,674; BH-adjusted *p* = 1.89 × 10^−5^), myocardial infarction (BH-adjusted *p* = 2.14 × 10^−5^), and acute myocardial infarction (BH-adjusted *p* = 7.79 × 10^−4^). These seven unique FDR-significant cardiovascular terms constituted the disease-enrichment profile displayed in Figure S16. No disease term was manually introduced, and clinically inappropriate substring matches and exact cross-domain duplicates were excluded before presentation (Tables S53 and S54). None of the prespecified sleep-related disease terms reached the within-library FDR threshold. The tested terms included sleep disorders, circadian rhythm disorders, insomnia-related phenotypes, short sleep phenotypes, and sleep duration traits, whereas no eligible tested term matched the prespecified sleep deprivation or sleep loss domain. Candidate-level annotation showed that BUD13 occurred among the contributing genes of FDR-significant CAD terms, whereas the observed SF3A3 and SF3B2 disease-domain overlaps were confined to non-significant terms (Table S55).

Because the 830-gene layer was analyzed without regard to expression direction, these findings indicate disease-associated gene set convergence and biological context rather than directional regulation, causal mediation, target validation, or therapeutic efficacy.

### 3.19. Transcriptional Architecture Is Preserved During Plaque Progression but Diverges During Aneurysm Enlargement

**Figure 10:**
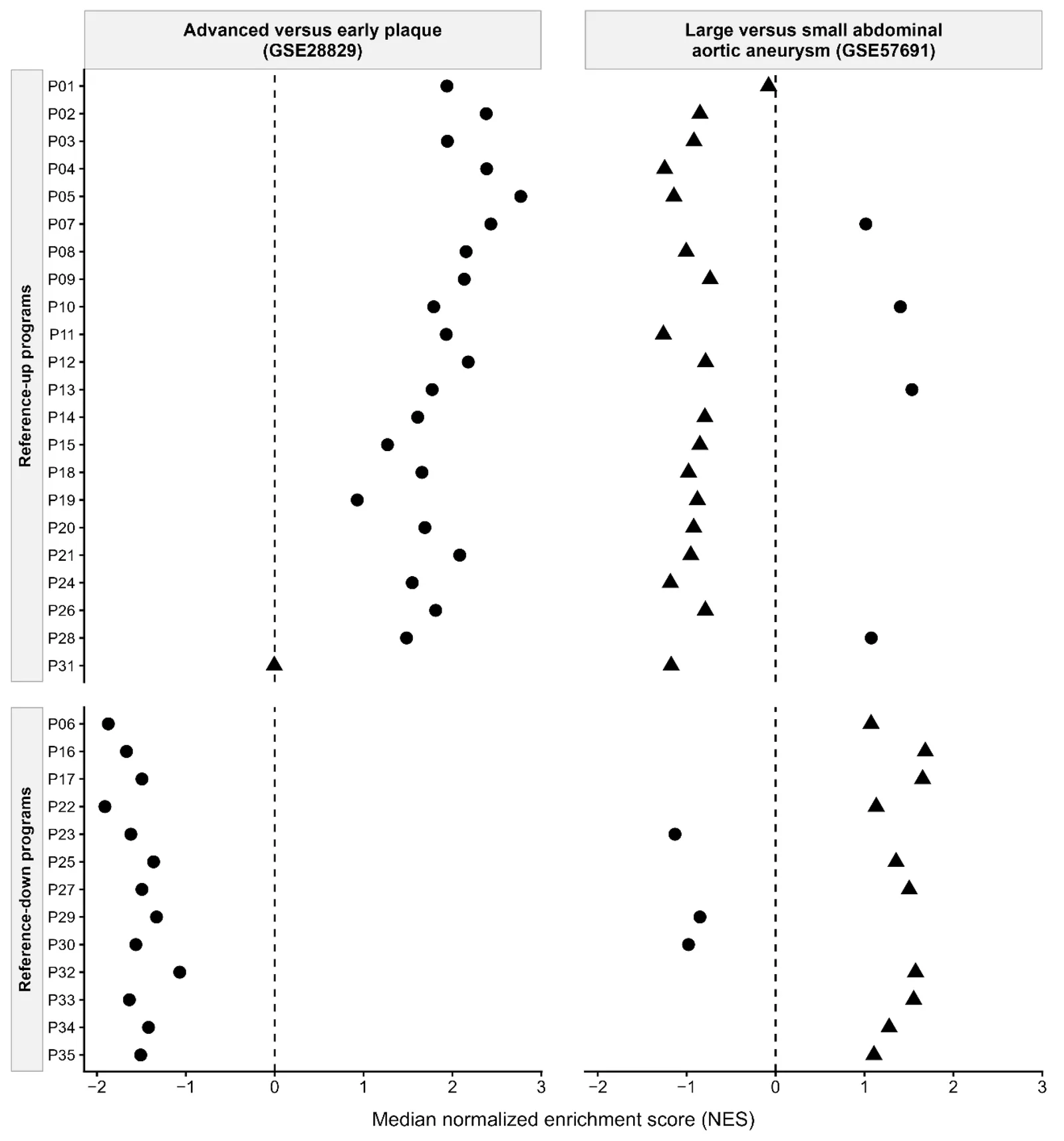
Cross-dataset directional concordance of the locked sleep-loss-associated transcriptional program architecture across vascular disease contexts. The 35 fixed transcriptional programs are shown for advanced versus early atherosclerotic plaque (GSE28829) and large versus small AAA (GSE57691), with program identities defined in Table S47. Each symbol represents one fixed program and is positioned according to the median NES of its constituent gene sets in the corresponding disease contrast. Programs are displayed in separate reference-up and reference-down blocks according to their locked GSE43292 reference direction; this grouping is descriptive and does not alter program membership or ranking. Positive NES values indicate enrichment toward advanced plaque in GSE28829 and toward large AAA in GSE57691, whereas negative values indicate enrichment in the opposite direction. Program direction was defined by the sign of the program-level median NES. Circles denote agreement with the locked GSE43292 reference direction, whereas triangles denote disagreement; no minimum effect-size threshold was imposed. The vertical dashed line marks zero enrichment. Thirty-four of 35 programs were directionally concordant in GSE28829, whereas 7 of 35 were directionally concordant in GSE57691.

To determine whether the locked sleep-loss-associated vascular architecture was preserved beyond the original plaque comparison, the 35 prespecified transcriptional programs were examined across two distinct disease trajectories. In GSE28829, which contrasts advanced with early atherosclerotic plaque, 34 of 35 programs retained the direction defined by the GSE43292 reference architecture, yielding a concordance of 97.1% (95% bootstrap CI, 91.4–100.0%; Figure 10 and Table 9). Thirty-two programs contained at least one constituent gene set with FDR < 0.05. This program-level continuity was paralleled at the gene level: 681 of 827 evaluable genes from the locked 830-gene set were directionally concordant (82.3%), and their log_2_ fold changes were strongly correlated with the corresponding GSE43292 effects (Spearman ρ = 0.745, *p* = 4.49 × 10^−147^; Table 9). The corresponding analysis in GSE57691 revealed a substantially different pattern. In large versus small AAA, only 7 of 35 programs retained the reference direction (20.0%; 95% bootstrap CI, 8.6–34.3%), whereas 28 programs showed opposite-direction behavior (Figure 10 and Table 9). Seven programs contained at least one constituent gene set with FDR < 0.05. At the gene level, 355 of 780 evaluable locked genes were directionally concordant (45.5%), and the relationship with the GSE43292 reference effects was weakly inverse (Spearman ρ = −0.144, *p* = 5.55 × 10^−5^; Table 9).

The 12 prespecified MCC-supported candidates showed a similar context-dependent pattern, with greater directional preservation during plaque progression than during aneurysm enlargement. Because these candidates belong to a densely interconnected spliceosomal network, their individual effects were considered descriptive rather than independent inferential observations and are presented in Figure S17. Complete program-, constituent gene set-, locked gene-, and MCC-candidate results are provided in Table S56.

Taken together, these findings indicate that the transcriptional architecture associated with sleep loss is largely maintained along the trajectory of atherosclerotic plaque progression, but is not uniformly conserved across a biologically distinct vascular disorder. Accordingly, this analysis is interpreted as cross-dataset directional and pathway concordance, not as external validation or replication.

### 3.20. Multilayer Support Profiles of the Prespecified MCC-Supported Candidates

The 12 prespecified candidates exhibited distinct and non-uniform multilayer support profiles, indicating that no single analytical layer accounted for their retention within the sleep loss–atherosclerosis framework (Table S57). Six candidates were represented in the retained sleep-associated WGCNA modules. *SF3B1* and *PRPF38A* showed high intramodular membership, with kME values of 0.916 and 0.840, respectively. *CWC27*, *SNRPB2*, *CWC22*, and *SNIP1* were also assigned to retained modules, although their kME values remained below the prespecified 0.80 threshold. The remaining candidates had no retained sleep-associated module membership; this classification did not exclude their presence within the broader WGCNA network or their support from non-co-expression layers.

Regulatory context overlap was restricted to *SF3A3*, which intersected the FDR-significant *ZSCAN21* and *TAF9B* target signatures. All 12 candidates were measurable in human atherosclerotic plaque cells, with predominant expression distributed across foam cells, dendritic cells, natural killer cells, B cells, and mast cells, thereby providing cell-type context without implying cell-specific functional validation. Primary exact-cis MR was estimable for 10 candidates. Higher genetically predicted *SF3A3* expression was associated with lower odds of CAD after correction for multiple testing (OR = 0.851, 95% CI 0.772–0.939; BH-adjusted *p* = 0.0150). Regional colocalization yielded PP.H4 = 0.831 under the primary prior, supporting a shared regional signal within the single-causal-variant framework, although this inference remained sensitive to prior specification. *SNIP1* also retained an FDR-significant MR association (OR = 0.933, 95% CI 0.889–0.979; BH-adjusted *p* = 0.0306); however, its regional posterior favored distinct signals (PP.H4 = 0.051), precluding interpretation as convergent MR colocalization support. *SNRPF* showed a nominal single-instrument association (OR = 0.821, 95% CI 0.701–0.961; *p* = 0.0143), but this did not remain significant after multiple-testing correction and the regional posterior was inconclusive (PP.H4 = 0.162). *SNRPD1* and *RBMX2* were not estimable under the prespecified exact-cis instrument criteria.

During progression from early to advanced plaque, 10 of the 12 candidates retained the direction of the locked reference effect. This continuity was interpreted as transcriptional consistency across plaque stages rather than as independent validation or evidence of causal involvement. Taken together, the multilayer synthesis identified SF3A3 as having the most integrated genetically informed profile, while *SNIP1* showed MR support without regional shared-signal support and *SF3B1* and *PRPF38A* showed the strongest sleep-associated co-expression context. The remaining candidates were characterized by varying combinations of network topology, plaque cell localization, transcriptional continuity, and candidate-specific regulatory or genetic annotations, supporting a profile-based rather than rank-based interpretation.

### 3.21. Candidate Convergence on RNA-Processing Programs Across Plaque Cell Contexts

**Figure 11:**
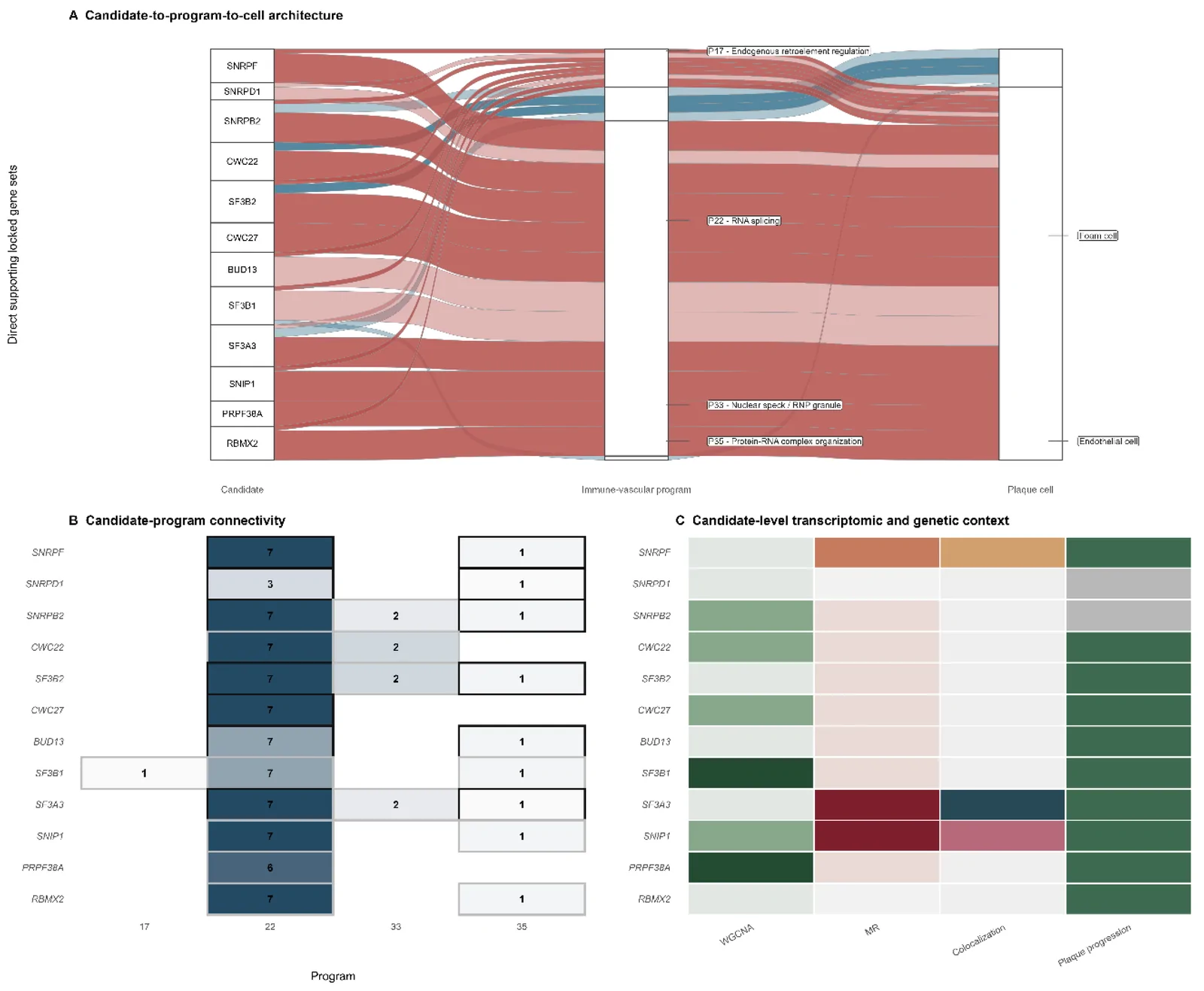
Integrative mapping of prespecified candidate targets to immune–vascular transcriptional programs, plaque cell contexts, and genetically informed annotations. (**A**) Alluvial representation of the 12 prespecified MCC-supported candidates, their directly associated locked immune–vascular programs, and the predominant plaque cell population assigned to each program. Candidate–program links required candidate membership in the leading-edge genes of at least one locked constituent gene set. Flow width represents the number of directly supporting gene sets. Red and blue flows denote programs predominantly localized to foam cells and endothelial cells, respectively; darker flows indicate that at least one supporting gene set contained the candidate in both cohort-specific leading-edge maps, whereas lighter flows indicate support from one map. (**B**) Candidate–program connectivity matrix. Cell values indicate the number of directly supporting locked gene sets; darker shading represents a larger number of supporting gene sets. Black borders indicate concordance between candidate- and program-level predominant plaque cell contexts, whereas grey borders indicate distinct cellular contexts. (**C**) Candidate-level transcriptomic and genetic context across sleep-associated WGCNA membership, primary exact-cis MR, regional colocalization, and directional behavior during plaque progression. These annotations were added after candidate–program mapping and did not determine link formation. In panel (**C**), dark green indicates high WGCNA module membership, light green indicates retained module membership below the hub threshold, and pale green indicates no retained module membership; dark red indicates an FDR-significant MR association, muted red indicates nominal significance only, pale red indicates a non-FDR-significant association, and light grey indicates that MR was not estimable. Dark blue denotes regional shared-signal support, mauve denotes evidence favoring distinct signals, orange denotes an inconclusive regional result, and light grey denotes that colocalization was not assessed. For plaque progression, green indicates directional concordance and grey indicates the opposite direction.

Direct leading-edge mapping placed all 12 prespecified MCC-supported candidates within at least one component of the locked immune–vascular transcriptional architecture. Of the 71 directionally concordant constituent gene sets, 11 contained at least one candidate in a cohort-specific leading edge, yielding 97 candidate–gene set relationships and 26 distinct candidate–program links (Figure 11 and Figure S18; Table S58). Candidate–program assignment was based exclusively on direct leading-edge membership and was not inferred from inclusion in the broader 830-gene intersection.

The resulting architecture was dominated by Program 22, an RNA-splicing program that was connected to all 12 candidates. Program 35, representing protein–RNA complex organization, was linked to nine candidates; Program 33, comprising nuclear speck and ribonucleoprotein granule biology, was linked to four; and Program 17, related to endogenous retroelement regulation, showed a single direct connection with SF3B1. Thus, the prespecified candidates converged principally on RNA processing biology while retaining more selective associations with nuclear organization and retroelement regulatory programs.

Plaque cell localization further resolved this program structure. Programs 22 and 35 were predominantly localized to foam cells, whereas Programs 17 and 33 were predominantly localized to endothelial cells. Thirteen of the 26 candidate–program links showed concordance between candidate- and program-level predominant plaque cell contexts; the remaining 13 connected distinct cellular compartments. Concordant links were driven mainly by foam-cell-localized candidates mapping to the foam-cell-associated RNA-splicing and protein–RNA complex programs. Conversely, candidates predominantly localized to dendritic cells, natural killer cells, B cells, or mast cells remained connected to programs localized principally to foam cells or endothelial cells, indicating that the shared transcriptional architecture extended across plaque cell compartments rather than being restricted to a single cellular lineage.

Eighteen candidate–program links included at least one supporting gene set in which the candidate occurred in both cohort-specific leading edges, whereas eight were supported only in one cohort-specific map. This annotation described the cross-cohort distribution of leading-edge membership but did not alter link definition. Sleep-associated WGCNA membership, regulatory context, exact-cis MR, regional colocalization, and plaque-progression directionality were subsequently overlaid as independent candidate-level annotations and were not used to create, weight, or rank the candidate–program relationships.

Collectively, this integrative analysis positioned the 12 candidates within a coherent RNA-processing-centered vascular framework spanning foam cell and endothelial cell programs, while preserving candidate-specific differences in cellular localization, co-expression context, and genetically informed support.

### 3.22. Descriptive Perturbation-Signature Prioritization and Interaction Annotation

#### 3.22.1. DGIdb Interaction Annotation Remained Distinct from Transcriptional Reversal

DGIdb annotation of the 830-gene analytical universe yielded 5674 distinct gene–drug pairs. Of the 830 genes, 152 were represented exclusively by target-engagement evidence, 104 by both target-engagement and variant-response evidence, and 42 exclusively by variant-response evidence; no DGIdb interaction was returned for the remaining 532 genes. These annotations were analyzed independently of LINCS transcriptional reversal and were not incorporated into a joint compound–target score or therapeutic ranking. Among the 12 prespecified MCC-supported candidates, recorded DGIdb gene–drug pairs were returned only for SF3B1 and SF3B2. The three SF3B1 pairs originated from variant-response sources, whereas the single SF3B2 target-engagement record did not provide a named compound. No DGIdb gene–drug interaction was returned for SF3A3, despite its convergent transcriptomic, network, Mendelian randomization, and colocalization support (Figure S19; Table S59). This negative annotation should not be interpreted as evidence that SF3A3 is pharmacologically intractable; rather, the present database interrogation identified no named compound with recorded direct interaction evidence for this candidate. Accordingly, the LINCS and DGIdb layers provide complementary hypothesis-generating pharmacological context but do not establish target engagement, therapeutic tractability, or efficacy.

#### 3.22.2. Perturbagen Prioritization and Program Context

**Figure 12:**
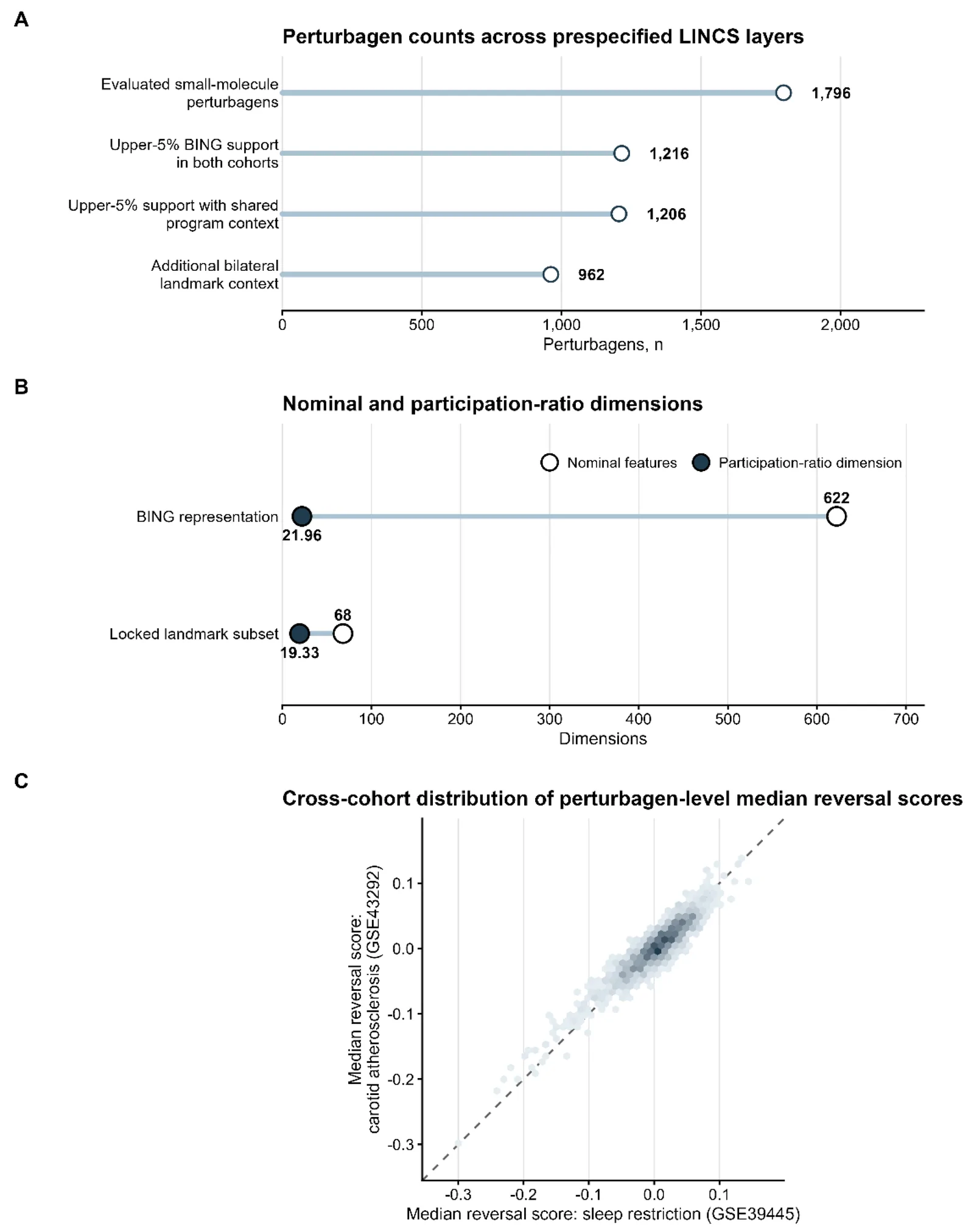
LINCS perturbagen flow, query space dimensionality, and cross-cohort reversal-score dependence for the locked shared sleep loss–atherosclerosis gene set. (**A**) The GSE70138 analysis included 107,404 small-molecule perturbation signatures representing 1796 unique perturbagens. Cross-cohort upper-5% membership in the BING representation was observed for 1216 perturbagens; 1206 of these had context for at least one shared locked program, and 962 additionally showed bilateral directional context in the locked landmark subset. (**B**) Nominal feature counts and participation-ratio effective dimensions for the 622-feature BING representation and its nested 68-feature locked landmark subset. Open circles denote nominal feature counts (622 and 68), and filled circles denote participation-ratio effective dimensions (21.96 and 19.33, respectively). Thus, the 554 additional inferred features increased effective dimensionality by 2.64. (**C**) Hexagonal binning of perturbagen-level median reversal scores in the sleep restriction and carotid atherosclerosis cohorts. Darker hexagons indicate a greater number of perturbagens per bin (range, 1–65), and the dashed line denotes identity. The cross-cohort Spearman correlation was ρ = 0.898 and is reported as a dependence diagnostic, not as evidence of independent replication. No compound ranking, composite score, claim of statistical significance, claim of therapeutic efficacy, or assessment of clinical actionability was derived from this layer.

The GSE70138 analytical universe comprised 107,404 small-molecule perturbation signatures representing 1796 unique perturbagens. In each primary cohort, 5371 signatures satisfied the prespecified positive score and upper 5% release-relative criterion. At the perturbagen level, 1216 compounds had at least one qualifying signature in both the sleep restriction and carotid atherosclerosis cohorts; 1206 of these also showed context for at least one shared locked program, and 962 additionally showed bilateral directional context in the locked landmark subset (Figure 12A; Table S60, panel S60A). Program context was therefore used to localize perturbagen-level signals within the locked transcriptional architecture, whereas landmark behavior served as a directional sensitivity analysis rather than as a primary selection criterion. Calibration analyses showed that the former pooled permutation-derived q-value requirement did not define an additional membership layer: in both cohorts, it selected exactly the same 5371 signatures as the positive upper 5% criterion (Figure S20; Table S60, panel S60B). More extensive permutation-null, effective-dimensionality, fixed-size comparator, block-granularity, and random-query analyses further demonstrated substantial dependence on gene-space covariance structure and were therefore treated as calibration diagnostics rather than as additional evidence of statistical significance or compound selection (Figures S21–S24; Tables S60 and S61). The participation ratio was 21.96 for the 622-feature BING representation and 19.33 for the nested 68-feature landmark subset; thus, the 554 additional inferred features increased estimated effective dimensionality by only 2.64 dimensions (Figure 12B; Table S60, panel S60D).

#### 3.22.3. Coverage Dependence and Cross-Cohort Dependence Constrain Interpretation

Program context was widespread rather than strongly selective: 32 of the 35 locked programs were estimable in both primary BING cohorts, and 1780 of 1796 perturbagens (99.1%) showed context for at least one shared program. Among the 1216 perturbagens satisfying the cross-cohort upper-5% criterion, 1206 had shared program context. Perturbagen membership was also strongly dependent on representation within the LINCS release, increasing from 36.4% among perturbagens represented by 1–10 signatures to 100% among those represented by more than 300 signatures (Figure S23; Table S60, panels S60E–S60G). This coverage dependence was retained as an explicit limitation rather than corrected by post hoc weighting or re-ranking. Perturbagen-level median BING reversal scores were strongly correlated between the two discovery cohorts (Spearman ρ = 0.898; Figure 12C; Table S60, panel S60H). Cross-cohort agreement was therefore interpreted as concordant descriptive prioritization rather than as independent replication. Complete perturbagen-level results, including per-cohort signature counts, reversal-score summaries, shared program identifiers, and landmark context, are provided in Table S60, panels S60I–S60J.

### 3.23. Sensitivity of the Discovery Architecture to Representative Probe Selection

The robustness of the GSE39445 discovery architecture to representative probe selection was examined using the contrast-independent maximum IQR specification described in Section 2.2. Among the 7669 gene symbols represented by more than one annotated probe, the selected representative probe changed for 4428 (57.7%), corresponding to 22.7% of all 19,541 measured gene symbols. At the gene level, 1046 genes met both differential expression criteria under maximum IQR selection, compared with 1352 under the submitted rule. Of these, 1021 were identified under both specifications, and 1015/1021 (99.4%) retained the same direction. The GSE43292 arm and the 16,869-gene cross-cohort universe were held fixed. Within this universe, the number of genes meeting both criteria in both cohorts decreased from 274 to 190. Accordingly, the overlap changed from 1.200-fold that expected under independence under the submitted specification (274 observed versus 228.35 expected; one-sided hypergeometric *p* = 3.54 × 10^−4^) to 1.096-fold under maximum-IQR selection (190 observed versus 173.41 expected; one-sided hypergeometric *p* = 0.082). Directional concordance among the shared genes remained limited (103/190, 54.2%).

The effect of probe selection differed between statistical significance and directional organization at the higher levels of the analysis. Under maximum IQR selection, 42 gene sets were significant in both cohorts, of which 37/42 (88.1%) were directionally concordant. Of the 91 gene sets defining the submitted architecture, 38 remained significant in both cohorts, whereas all 91 retained their submitted directional classification (71 concordant and 20 discordant). At the program level, the prespecified 50-program membership was retained without re-clustering rather than independently reconstructed. Under this fixed architecture, all 50 programs preserved their sleep cohort enrichment direction, and the same 35/50 programs remained directionally concordant across cohorts, comprising the same 22 positively concordant and 13 negatively concordant programs. Thus, maximum IQR probe selection yielded a smaller and no longer nominally significant gene-level cross-cohort overlap, while the directional classification of the submitted gene sets and the composition and direction of the locked program-level convergence were preserved (Table S62).

## 4. Discussion

Whether human sleep loss and human atherosclerotic tissue share a transcriptional response depends on the resolution at which convergence is measured. Under the submitted representative probe specification, the discovery cohorts shared more differentially expressed genes than expected under independence (274 of 16,869; hypergeometric *p* = 3.54 × 10^−4^), yet only 48.9% changed in the same direction, indistinguishable from the conventional (*p* = 0.66) or marginal (*p* = 0.85) null. The gene-level overlap was sensitive to representative probe selection: under the contrast-independent maximum IQR specification, 190 genes were shared, corresponding to 1.096-fold the overlap expected under independence, and the enrichment was no longer nominally significant (*p* = 0.082). In contrast, directional organization was preserved at the higher levels of the submitted architecture: all 91 gene sets retained their directional classification, and the same 35 of 50 fixed programs remained directionally concordant. The principal evidence for cross-compartment convergence therefore resides in the directional organization of transcriptional programs rather than in enrichment of overlapping individual genes. The prevailing design instead intersects two gene lists and treats the overlap as the shared substrate: Yang and colleagues [15] applied it to insomnia and atherosclerosis, nominating *IL10RA*, *CCR1* and *SPI1*; Wang and colleagues [9] combined six machine-learning algorithms with SHapley Additive exPlanations (SHAP) interpretation to prioritize *S100A3* and *VEGFB* as blood biomarkers. Both optimized a diagnostic signature; the present study did not. Across compartments such an intersection is directionally uninformative: 140 of our 274 shared genes moved in opposite directions.

The convergent architecture pairs enrichment of neutrophil degranulation, granule, and myeloid effector programs with depletion of RNA processing, chromatin, and ciliary programs. Much of the mechanistic evidence linking sleep and circadian disruption to vascular pathology has been generated in animal models [74]; both sides of our comparison are human, one experimentally controlled. Emergency myelopoiesis associated with plaque-infiltrating neutrophils [75] and sleep-deprivation-induced neutrophil hyperactivation via a conserved lactate–H3K18 lactylation–RORA axis [76] provide mechanistic context for our positively concordant immune block; neutrophils were the only immune population meeting the four-layer directional criterion. Our data are consistent with that axis and do not demonstrate it. The modest sleep-arm amplitude is expected: circulating inflammatory proteins rise only after several nights of restriction [77]. Proteome-wide MR has independently nominated inflammatory targets for coronary atherosclerosis [78], approaching the same axis at the protein rather than the transcript level.

The negatively concordant half is the less anticipated result. All twelve highest-scoring proteins by MCC formed a compact spliceosomal module, and splicing is now recognized as an active contributor to vascular disease, with more than 5000 alternative-splicing events distinguishing early from advanced fibroatheroma [79]. Human genetics discriminated within it: *SF3A3* alone combined FDR-significant exact-cis MR (OR 0.851; 95% CI, 0.772–0.939) with regional colocalization (PP.H4 = 0.831), its direction coherent with the observed depletion, though coherence is not mediation. *SNIP1* was single-variant-sensitive and did not colocalize; *SNRPF* remained nominal.

Mechanistically, the coordinated depletion of RNA processing and spliceosomal programs is compatible with perturbed RNA processing homeostasis, but bulk transcriptomic data do not establish a specific splicing defect. Consistent with the identity of this module, the U2-type spliceosomal complex annotation was recovered among the 828 mapped protein nodes with 28 observed genes against a STRING background of 93 (GO:0005684; FDR = 2.15 × 10^−12^; Table S41), and all twelve topology-prioritized candidates were annotated to this term. In human aortic endothelial cells, inflammatory stimulation with IL-1β induces extensive transcript-isoform remodeling, including 1224 differentially spliced transcripts, and genetically regulated splicing events can colocalize with cardiovascular risk loci [80]. Experimental perturbation of the regulatory splice factor PTBP1 further demonstrates that splicing regulation can influence endothelial NF-κB activation, adhesion molecule expression, myeloid recruitment, and atherosclerotic lesion formation [81]. These precedents establish functional links between RNA processing and vascular inflammatory state, but they should not be interpreted as directional equivalents of the coordinated depletion observed here; regulatory RNA-binding proteins that influence splice-site selection are not interchangeable with the spliceosomal/U2-associated machinery prioritized in our analysis. The shared depletion may therefore reflect at least two non-exclusive processes: altered cellular composition across the sampled compartments or cell-intrinsic redistribution of RNA processing activity under inflammatory and metabolic stress. The observation that seven of the twelve prioritized candidates showed their highest average expression in plaque foam cells places this signal within a disease-relevant cellular compartment, but does not demonstrate that reduced transcript abundance causes altered splicing in those cells. Notably, the genetically informed association for *SF3A3* was directionally compatible with lower CAD odds at higher genetically predicted expression, providing an orthogonal rationale for experimental follow-up rather than evidence that the observed transcript depletion mediates disease. Distinguishing these possibilities will require splicing-resolved transcriptomics in defined vascular and immune cell populations, ideally coupled to perturbation of the prioritized candidates.

A comparable design has carried druggable-genome genes through two-sample and single-cell MR in coronary atherosclerosis [82]; here the candidate set was fixed by network topology before any genetic analysis, so this layer tested a prespecified set rather than defining one. Cell-type resolution has since been reached directly by combining cis-MR with colocalization across immune cell types in atherosclerotic cardiovascular disease [83], a level our bulk-derived exposure data cannot attain and the natural extension of this layer.

Transferred unchanged, the architecture generalized to acute total sleep deprivation (32 of 35; 91.4%), was almost fully preserved during plaque progression (34 of 35; 97.1%), but showed predominantly opposite-direction behavior during aneurysm enlargement (7 of 35 concordant; 20.0%). This addresses a key concern about program-level aggregation: in plaque the program estimate exceeded the gene estimate (97.1% versus 82.3%), whereas in aneurysm it fell below it (20.0% versus 45.5%), indicating that aggregation did not systematically favor concordance across these two disease contexts.

The GSE57691 finding is important because it places a clear limit on how far the locked architecture can be generalized. The same program set that was strongly preserved during atherosclerotic plaque progression was not preserved during aneurysm enlargement, and this divergence was accompanied by weak inverse correspondence at the gene level. That pattern argues against viewing the sleep loss–atherosclerosis architecture as a generic signature of vascular injury or remodeling. It also strengthens the methodological interpretation of the program analysis, because redundancy collapse did not consistently increase concordance across disease contexts. At the same time, the aneurysm result should not be taken as evidence of a biologically reversed atherosclerotic program. No individual gene reached the prespecified FDR threshold in GSE57691, and the conservative distribution of test statistics does not allow disease-specific biology to be separated confidently from cohort- or dataset-level effects. The more defensible interpretation is therefore one of context dependence rather than biological reversal. The architecture is substantially preserved along an atherosclerotic progression axis, but not uniformly across vascular remodeling states. In this sense, GSE57691 serves not as a failed replication, but as an informative limit on the generalizability of the principal finding.

A prespecified separability analysis returned a negative-to-borderline answer: the primary program–LASSO model reached an AUC of 0.558 with a participant cluster bootstrap interval including 0.5 and a permutation *p* value of 0.052; the 830-gene representation gave 0.474. The contrast with the 0.931 and 0.745 reported for a comparable nomogram [15] should be interpreted cautiously because validation design can materially affect apparent performance; repeated-subject leakage and non-nested feature selection are recognized sources of substantial performance inflation [84]. Reproducible convergence and individual-level classifiability are distinct questions.

The novelty here is architectural rather than topical. Yang and colleagues [15] also used the same public plaque single-cell dataset, so we claim no priority for its use, for single-cell localization, machine learning or immune profiling here; our cluster and cell-type counts differ from theirs, a consequence of divergent quality control, integration, and annotation choices that we report rather than reconcile. A contemporaneous study applied essentially the same pipeline to type 2 diabetes and atherosclerosis, combining overlapping genes, enrichment, protein–protein interaction analysis, hub prioritization, machine learning, deconvolution and single-cell mapping to nominate shared biomarkers [85]. To our knowledge, what is new is the separation, within one prespecified analysis linking controlled human sleep loss to human atherosclerotic vascular tissue, of numerical gene overlap, directional gene-level concordance, redundancy-collapsed program convergence, cross-context preservation, and participant-grouped separability.

The extended LINCS calibration further resolved how the locked landmark representation should be interpreted within the perturbation signature analysis. At a fixed 68-feature size, landmark score dispersion fell within the empirical distributions of both random BING subsets and constrained matched inferred subsets in both cohorts, indicating that the dispersion behavior of the locked landmark gene set was not exceptional relative to these 68-feature comparators. This result does not, however, isolate gene composition from covariance structure: the constrained matched subsets were highly overlapping and had substantially lower effective dimensionality, while blockwise and unrestricted *q* < 0.05 counts were uniformly zero across all three 68-feature arms and were therefore non-discriminating rather than evidence of equivalence. The extended analysis consequently strengthens the calibration framework for interpreting the LINCS results without converting permutation-derived *q* values into evidence of statistical validation or altering the release-relative perturbagen prioritization.

Limitations bound the interpretation, in order of consequence. The comparison is cross-compartment by construction: whole blood and carotid artery differ in composition, sampling and timescale, so a shared program indicates a common transcriptional state rather than shared cellular involvement; bulk data cannot separate state from composition. The 830-gene set and 35 programs were defined from the complete discovery data, so the network, enrichment, and candidate–program layers measure internal cohesion, not independent confirmation. Gene sets remain hierarchically related after collapse, so program-level binomial *p* values are nominal summaries against both conventional and marginal nulls, the bootstrap interval carrying the inferential weight. The submitted representative probe rule was informed by full-cohort differential expression; the contrast-independent maximum IQR sensitivity analysis showed that this choice materially affected gene-level significance and cross-cohort overlap, while preserving the directional classification of the submitted gene sets and the fixed program-level architecture. Feature selection for the within-cohort separability analysis was nevertheless not nested, so the resulting discrimination remains an upper-bound estimate. Study day and exposure are confounded in the acute deprivation protocol; the aneurysm reversal arose in a cohort with no FDR-significant gene, so its origin—disease axis or dataset-level shift—is unresolved and is described rather than interpreted as reversed biology. Genetic inference is additionally constrained by instrument scarcity rather than by evidence of weak instrument strength. Both retained *SF3A3* instruments exceeded the prespecified strength threshold by a wide margin and yielded directionally concordant Wald estimates; nevertheless, only two independent harmonized variants were available. This precluded the prespecified weighted-median and MR-Egger analyses and left Cochran’s Q with a single degree of freedom and limited ability to detect between-instrument inconsistency. The absence of detected heterogeneity should therefore not be interpreted as exclusion of horizontal pleiotropy. Steiger analyses consistently supported the exposure-to-outcome direction, but directionality testing does not establish instrument validity. Accordingly, the *SF3A3* MR estimate is interpreted as genetically informed support rather than stand-alone causal evidence and should be considered alongside, rather than validated by, the regional colocalization result, which itself remains prior-sensitive and conditional on a single-causal-variant model. Exposure data derive from other tissues, and two candidates were non-estimable. Single-cell data derive from twelve donors and served for localization only. All evidence is computational: no candidate was experimentally validated and no compound tested.

Priorities are re-projection of the locked programs and candidates onto finer plaque references [86], extension of the genetic layer to splicing quantitative trait loci [79], and experimental interrogation of the spliceosomal module. Pharmacological expectations should remain restrained: no splice-modulating therapy is approved for a cardiovascular indication [79], and transcriptome-based repurposing prioritizes statistically prominent rather than pharmacologically targetable genes and depends on cancer-derived perturbation resources [72]. These are testable candidates, not tractable targets.

## 5. Conclusions

Experimentally imposed human sleep loss and human atherosclerotic vascular tissue share a directionally coherent transcriptional architecture that is invisible at the level of individual genes. Gene-level overlap lacked directional coherence and its statistical enrichment was sensitive to the representative probe rule, whereas the higher-order directional architecture was preserved under contrast-independent maximum IQR selection: the same 35 of 50 redundancy-collapsed programs remained concordant, and 32 of those 35 generalized without modification to acute total sleep deprivation. The architecture couples enrichment of neutrophil granule and myeloid effector programs with depletion of RNA processing, chromatin regulatory, and ciliary programs, was preserved during plaque progression but diverged substantially during aneurysm enlargement, and prioritized *SF3A3* as the single candidate with convergent transcriptomic, network and genetically informed support. The same programs did not reliably separate individual samples.

Cross-compartment convergence between sleep loss and atherosclerosis is therefore demonstrable at the level of coordinated transcriptional programs, but it should be interpreted as convergence between distinct biological compartments rather than as replication or evidence of shared cellular involvement. It should not be reinterpreted as diagnostic separability or uniform vascular generalization and yields experimentally testable candidates rather than validated therapeutic targets.

## Figures and Tables

**Table 1:** Differential expression summary across the analyzed sleep loss and vascular cohorts. Gene counts refer to unique, non-empty, platform-provided gene symbols retained after the cohort-specific deterministic probe-to-gene procedure. Statistical significance was defined by a BH FDR-adjusted *p* value below 0.05 and, where indicated, the additional prespecified effect size criterion |log_2_ fold change| > log_2_(1.2). Upregulation and downregulation are defined relative to sleep restriction in GSE39445, carotid plaque in GSE43292, advanced plaque in GSE28829, and large AAA in GSE57691.

Dataset	Comparison	Samples	Gene-Level Features	FDR < 0.05	FDR < 0.05 and Effect-Size Threshold	Upregulated	Downregulated
GSE39445	Sleep restriction versus extension	438	19,541	4390	1352	645	707
GSE43292	Plaque versus intact artery	64	21,147	8012	3719	1946	1773
GSE28829	Advanced versus early plaque	29	24,442	2342	2218	1202	1016
GSE57691	Large versus small AAA ^1^	49	25,235	0	0 ^2^	0	0

^1^ Of the 68 deposited samples, 49 belonged to the two aneurysm groups; nine aortic occlusive disease samples and ten organ-donor samples were excluded. ^2^ No gene reached the prespecified FDR threshold. This absence was not interpreted as evidence of equivalence.

**Table 2:** Differential expression associated with plaque progression in GSE28829.

Variable	Value
Dataset	GSE28829
Cohort role	Plaque progression cohort
Comparison	Advanced versus early plaque
Statistical model	Unpaired limma
Raw probes	54,675
Samples, advanced/early	29, 16/13
Unique gene symbol features	24,442
Gene-level FDR < 0.05	2342
FDR < 0.05 and effect-size threshold	2218
Upregulated/downregulated	1202/1016
Distinct selected-probe measurements, all/signature	22,807/2091

**Table 3:** Context-specific aneurysm severity analysis in GSE57691. The analysis compared large with small AAAs after exclusion of aortic occlusive disease and organ donor samples. No gene met the prespecified FDR threshold. Permutation results are reported as a complementary assessment of the observed conservative test-statistic distribution.

Category	Parameter	Result
Cohort	Comparison	Large versus small AAA
Cohort	Samples, large/small	49, 29/20
Differential expression	Gene-level features tested	25,235
Differential expression	Nominal *p* < 0.05	56
Differential expression	Minimum raw *p* value	7.7 × 10^−4^
Differential expression	Minimum adjusted *p* value	0.99997
Differential expression	FDR < 0.05	0
Differential expression	FDR and effect size criterion	0
Permutation assessment	Observed versus null nominal count	56 versus 276, median
Permutation assessment	Lower-tail empirical *p* value (nominal count)	0.069
Permutation assessment	Lower-tail empirical *p* value (moderated-t dispersion)	0.084

**Table 4:** Within-cohort participant-grouped separability analysis in GSE39445. The analysis included 438 samples from 26 participants. Three prespecified feature representations were evaluated using five-fold participant-grouped cross-validation repeated five times. All samples from each participant were retained within the same cross-validation fold. CIs were estimated using participant cluster bootstrap resampling. The prespecified reference program-based LASSO analysis was evaluated using 1000 participant-blocked label permutations, whereas each secondary feature–model combination was evaluated using 100 permutations. AUC values correspond to the fixed reference repeat.

Representation	Features	Model	AUC	95% CI	CI Includes 0.5	Permutations	Permutation *p*
Programs, reference	35	LASSO logistic regression	0.558	0.486–0.630	Yes	1000	0.052
Programs	35	Random forest	0.575	0.515–0.641	No	100	0.069
Programs	35	Radial support vector machine	0.558	0.492–0.628	Yes	100	0.109
Gene sets	71	LASSO logistic regression	0.518	0.442–0.592	Yes	100	0.257
Gene sets	71	Random forest	0.567	0.503–0.627	No	100	0.069
Gene sets	71	Radial support vector machine	0.572	0.495–0.650	Yes	100	0.089
Shared leading-edge genes	830	LASSO logistic regression	0.474	0.408–0.532	Yes	100	0.485
Shared leading-edge genes	830	Random forest	0.566	0.484–0.643	Yes	100	0.109
Shared leading-edge genes	830	Radial support vector machine	0.537	0.461–0.619	Yes	100	0.238

**Table 5:** Cross-cohort directional integration of immune- and stromal-cell-associated transcriptional scores in the GSE39445 sleep-restriction and GSE43292 carotid atherosclerosis discovery cohorts. Panel (**A**) summarizes MCP-counter and prespecified marker score estimates for sleep restriction relative to sleep extension in GSE39445. Panel (**B**) summarizes the corresponding estimates for carotid plaque relative to patient-matched macroscopically intact arterial tissue in GSE43292. Panel (**C**) presents within-method cross-cohort directional concordance and the prespecified four-layer concordance classification. Estimates are expressed in within-population standard deviation units. Four-layer concordance was assigned only when all four cohort-by-method coefficients shared the same sign.

(A) GSE39445: Sleep Restriction Versus Sleep Extension					
Estimated population	MCP β	MCP FDR	Marker β	Marker FDR	Within-cohort direction
Fibroblasts	0.407	0.0091	0.373	0.0082	Yes
Neutrophils	0.151	0.1417	0.267	0.0260	Yes
B lineage	−0.197	0.0120	−0.041	0.5900	Yes
CD8 T cells	0.092	0.3652	−0.072	0.4568	No
Cytotoxic lymphocytes	−0.230	0.0339	−0.152	0.2662	Yes
Endothelial cells	−0.337	0.0032	0.120	0.4205	No
Monocytic lineage	−0.090	0.3569	0.146	0.1941	No
Myeloid dendritic cells	−0.046	0.7164	−0.108	0.2964	Yes
NK cells	−0.174	0.0953	−0.147	0.2662	Yes
T cells	−0.146	0.1209	−0.205	0.0348	Yes
(B) GSE43292: Plaque Versus Patient-Matched Intact Tissue					
Estimated population	MCP β	MCP FDR	Marker β	Marker FDR	Within-cohort direction
Fibroblasts	0.598	0.0017	0.181	0.3668	Yes
Neutrophils	0.776	4.71 × 10^−5^	1.046	7.64 × 10^−6^	Yes
B lineage	1.119	1.57 × 10^−5^	1.065	1.77 × 10^−5^	Yes
CD8 T cells	0.424	0.1056	0.766	0.0014	Yes
Cytotoxic lymphocytes	0.960	4.71 × 10^−5^	1.083	1.77 × 10^−5^	Yes
Endothelial cells	0.910	4.35 × 10^−5^	0.870	1.90 × 10^−5^	Yes
Monocytic lineage	1.119	2.27 × 10^−6^	1.147	5.06 × 10^−6^	Yes
Myeloid dendritic cells	1.110	2.27 × 10^−6^	1.063	1.06 × 10^−5^	Yes
NK cells	1.074	2.01 × 10^−6^	1.184	5.06 × 10^−6^	Yes
T cells	0.897	1.33 × 10^−4^	0.936	2.79 × 10^−5^	Yes
(C) Cross-Cohort Directional Integration					
Estimated population	MCP cross-cohort		Marker cross-cohort		Four-layer concordance
Fibroblasts	Yes		Yes		Yes
Neutrophils	Yes		Yes		Yes
B lineage	No		No		No
CD8 T cells	Yes		No		No
Cytotoxic lymphocytes	No		No		No
Endothelial cells	No		Yes		No
Monocytic lineage	No		Yes		No
Myeloid dendritic cells	No		No		No
NK cells	No		No		No
T cells	No		No		No

β, standardized regression coefficient; FDR, BH false discovery rate; MCP-counter, Microenvironment Cell Populations-counter; NK, natural killer. In GSE39445, sleep extension was the reference condition, such that a positive β indicates a higher estimated transcriptional score during sleep restriction. In GSE43292, patient-matched macroscopically intact arterial tissue was the reference condition, such that a positive β indicates a higher estimated score in carotid plaque. Cross-method direction denotes agreement in coefficient sign between MCP-counter and the prespecified marker score approach within the same cohort. MCP-counter and marker score cross-cohort concordance denote agreement in coefficient sign between GSE39445 and GSE43292 within the respective scoring method. Four-layer concordance required the MCP-counter and marker score coefficients to be directionally concordant within both cohorts and across cohorts, resulting in a common sign for all four cohort-by-method estimates. This classification evaluates directional convergence only and does not require statistical significance in every constituent analysis. Because the two scoring approaches use partially overlapping marker genes, agreement between them should not be interpreted as independent methodological validation. The reported scores represent relative transcriptome-derived cell-associated signals and do not constitute direct measurements of cellular fractions, absolute cell numbers, histologic infiltration, mechanistic mediation, or causality. Identical FDR values may occur because of ties generated by the BH step-up procedure.

**Table 6:** STRING network characteristics and representative functional enrichment of the locked shared leading-edge gene set. Panel (**A**) summarizes the submitted and mapped identifier counts, unmapped identifiers, observed and expected edge counts, average node degree, average local clustering coefficient, and the STRING-reported lower bound for the PPI enrichment *p* value. Panel (**B**) presents a biologically structured, redundancy-aware selection of representative terms capturing the principal innate immune myeloid, RNA-processing chromatin, and lipid-handling components of the network, together with additional ciliary, circadian, and metabolic annotations. Within closely overlapping annotation families, a single representative term was retained with reference to STRING signal, false discovery rate, annotation specificity, and consistency with the broader program architecture. This representative selection was used for concise biological interpretation and is distinct from the category-specific, signal-ranked terms displayed in Figure 6B–F. The input gene set was not partitioned according to enrichment direction; therefore, no term carries an upregulated or downregulated interpretation. The complete, unfiltered STRING enrichment output is provided in Table S41.

(A) STRING Network Characteristics							
Network Measure			Value		Network Measure		Value
Submitted gene symbols			830		Mapped protein nodes		828
Unmapped identifiers			*GGT3P*; *HSP90AB3P*		Observed edges		2755
Expected edges			1772		Average node degree		6.65
Average local clustering coefficient			0.433		STRING-reported lower bound for PPI enrichment *p* value		<1.0 × 10^−16^
(B) Representative STRING Functional Enrichment Results							
Functional Component	Source	Term ID	Term	Observed/Background Genes	Strength	Signal	FDR
Innate immune and myeloid effector functions	Reactome	HSA-6798695	Neutrophil degranulation	105/476	0.72	2.65	2.98 × 10^−36^
	Reactome	HSA-168249	Innate immune system	143/1041	0.51	1.77	3.25 × 10^−30^
	GO-CC	GO:0030141	Secretory granule	123/873	0.53	1.76	2.75 × 10^−27^
	GO-CC	GO:0042581	Specific granule	39/159	0.77	1.96	8.59 × 10^−15^
	GO-BP	GO:0050727	Regulation of inflammatory response	64/371	0.61	1.73	4.62 × 10^−17^
	GO-BP	GO:0050764	Regulation of phagocytosis	22/104	0.70	1.14	4.98 × 10^−7^
	GO-BP	GO:0045730	Respiratory burst	7/23	0.86	0.57	3.9 × 10^−3^
	KEGG	hsa04621	NOD-like receptor signaling pathway	27/173	0.57	0.94	3.23 × 10^−6^
RNA processing and chromatin regulation	GO-BP	GO:0008380	RNA splicing	101/370	0.81	3.22	7.48 × 10^−42^
	GO-BP	GO:0022613	Ribonucleoprotein complex biogenesis	106/449	0.75	2.84	3.77 × 10^−39^
	GO-BP	GO:0042254	Ribosome biogenesis	55/299	0.64	1.73	1.27 × 10^−15^
	GO-BP	GO:0006325	Chromatin organization	91/612	0.55	1.69	9.78 × 10^−21^
	GO-MF	GO:0042393	Histone binding	55/252	0.72	2.04	4.95 × 10^−18^
Lipid handling	GO-BP	GO:0010876	Lipid localization	37/365	0.38	0.64	1.3 × 10^−4^
	GO-BP	GO:0015918	Sterol transport	13/82	0.58	0.54	3 × 10^−3^
	GO-BP	GO:0010874	Regulation of cholesterol efflux	8/37	0.71	0.48	8.5 × 10^−3^
Additional ciliary, circadian, and metabolic context	Reactome	HSA-5617833	Cilium assembly	45/200	0.73	1.92	2.24 × 10^−15^
	GO-MF	GO:0015631	Tubulin binding	57/380	0.55	1.38	2.6 × 10^−12^
	Reactome	HSA-400253	Circadian clock	14/68	0.69	0.75	2.7 × 10^−4^
	KEGG	hsa04931	Insulin resistance	17/106	0.58	0.69	3.4 × 10^−4^

Abbreviations: FDR, BH false discovery rate; GO-BP, Gene Ontology Biological Process; GO-CC, Gene Ontology Cellular Component; GO-MF, Gene Ontology Molecular Function; PPI, protein–protein interaction. Strength is the base-10 logarithm of the observed-to-expected gene-count ratio; signal is the redundancy-weighted enrichment score reported by STRING. Term identifiers, observed and background gene counts, strength, signal, and FDR values were reproduced directly from the downloaded STRING version 12.0 enrichment output.

**Table 7:** Ten highest-ranked protein nodes according to complementary topological measures in the STRING-derived protein–protein interaction network.

Rank	Degree	Degree Value	Betweenness	Betweenness Centrality	Closeness	Closeness Centrality	MCC
1	*SNRPF*	56	*HSP90AA1*	0.3477	*HSP90AA1*	0.3646	*SNRPF*
2	*RPS27A*	55	*IFT88*	0.1936	*NPM1*	0.3408	*SNRPD1*
3	*SNRPD1*	50	*RPS27A*	0.1761	*HSP90AB1*	0.3384	*SNRPB2*
4	*RPS6*	49	*TNF*	0.1175	*RPS27A*	0.3333	*CWC22*
5	*TNF*	42	*TLR4*	0.1161	*UBC*	0.3323	*SF3B2*
6	*SF3B1*	41	*NPM1*	0.1139	*HDAC6*	0.3300	*CWC27*
7	*SRSF1*	41	*RPS6*	0.1098	*RPS6*	0.3296	*BUD13*
8	*HSP90AA1*	39	*HDAC2*	0.1070	*ESR1*	0.3288	*SF3B1*
9	*IL1B*	39	*IL1B*	0.1034	*RPL5*	0.3260	*SF3A3*
10	*FBL*	37	*HDAC6*	0.0916	*VCP*	0.3257	*SNIP1*

Note: Rankings were generated independently for each topological measure. Equal metric values were retained as ties and were not interpreted as evidence of a meaningful hierarchy. For fixed-length graphical and tabular displays, tied nodes crossing a display boundary were retained according to their order in the Cytoscape export. The same rule governed tied degree values within the ten-row table display, including ties at degree 39 and degree 37. Five MCC values affected by floating-point representation were rounded to the nearest integer in the complete node-level output. Minimal score differences within the highest MCC group were not interpreted as a biologically meaningful within-group hierarchy. The input gene set was not partitioned according to expression direction, and no protein listed in the table carries an upregulated or downregulated interpretation. Network position does not establish causal importance, disease specificity, direct regulation, or therapeutic efficacy. Complete metric-specific node-level results are provided in Table S42.

**Table 8:** Exact-cis expression instrument MR and regional genetic evidence for the 12 network-prioritized candidates. Genetic effects are reported per genetically predicted higher gene expression. The primary CAD outcome was ebi-a-GCST003116 (*N* = 141,217; 42,096 cases; 99,121 controls derived as the total sample size minus the number of cases). BH correction was applied across all 12 prespecified candidates, including candidates without an estimable instrument. Regional colocalization interpretations refer to the primary *p*_12_ = 1 × 10^−5^ prior.

Candidate	Instruments	Method	OR (95% CI)	*p* Value	BH-Adjusted *p*	Regional Genetic Interpretation
***SNRPF***	1 exact-cis SNP	Wald ratio	0.821 (0.701–0.961)	0.0143	0.0572	Nominal single-SNP association; not significant after correction across the 12 candidates; regional posterior distribution was inconclusive.
***SNRPD1***	Not estimable	Not estimable	Not estimable	Not estimable	Not estimable	No genome-wide-significant instruments were available from the prespecified exposure dataset.
***SNRPB2***	4 exact-cis SNPs	Inverse variance weighted	1.005 (0.965–1.047)	0.8154	1.0000	No FDR-significant exact-cis MR support.
***CWC22***	1 exact-cis SNP	Wald ratio	1.006 (0.826–1.227)	0.9505	1.0000	No FDR-significant exact-cis MR support.
***SF3B2***	1 exact-cis SNP	Wald ratio	0.993 (0.941–1.047)	0.7933	1.0000	No FDR-significant exact-cis MR support.
***CWC27***	1 exact-cis SNP	Wald ratio	1.103 (0.941–1.292)	0.2251	0.5415	No FDR-significant exact-cis MR support.
***BUD13***	2 exact-cis SNPs	Inverse variance weighted	0.955 (0.881–1.036)	0.2707	0.5415	No FDR-significant exact-cis MR support.
***SF3B1***	1 exact-cis SNP	Wald ratio	1.062 (0.959–1.176)	0.2459	0.5415	No FDR-significant exact-cis MR support.
***SF3A3***	2 exact-cis SNPs	Inverse variance weighted	0.851 (0.772–0.939)	0.00125	0.0150	FDR-significant exact-cis MR; PP.H4 = 0.831 under the primary *p*_12_ = 1 × 10^−5^ prior; shared-signal support was sensitive to the prior specification.
***SNIP1***	4 exact-cis SNPs	Inverse variance weighted	0.933 (0.889–0.979)	0.00510	0.0306	FDR-significant exact-cis MR, but the estimate was single-variant-sensitive and the regional posterior favored distinct eQTL and CAD signals.
***PRPF38A***	1 exact-cis SNP	Wald ratio	0.995 (0.908–1.090)	0.9156	1.0000	No FDR-significant exact-cis MR support.
***RBMX2***	Not estimable	—	—	—	—	No genome-wide-significant instruments were available from the prespecified exposure dataset.

Abbreviations: BH, Benjamini–Hochberg; CAD, coronary artery disease; CI, confidence interval; FDR, false discovery rate; MR, Mendelian randomization; OR, odds ratio; PP.H4, posterior probability of a shared causal variant; SNP, single-nucleotide polymorphism.

**Table 9:** Cross-dataset preservation of the sleep-loss-associated vascular transcriptional architecture. The locked GSE43292-derived transcriptional architecture was examined across two distinct vascular disease trajectories: progression from early to advanced atherosclerotic plaque and enlargement from small to large AAA. The table shows marked preservation of the reference architecture during plaque progression, contrasted with limited and partly opposite-direction behavior during aneurysm enlargement.

Disease Context and Analysis Level	Result
Advanced versus early atherosclerotic plaque—GSE28829	
Concordant fixed programs	34/35 (97.1%)
95% bootstrap CI	91.4–100.0%
Programs with ≥1 constituent gene set at FDR < 0.05	32/35
Evaluable locked genes	827/830
Concordant locked genes	681/827 (82.3%)
Spearman correlation with the GSE43292 reference effects	ρ = 0.745
Correlation *p* value	4.49 × 10^−147^
Large versus small AAA—GSE57691	
Concordant fixed programs	7/35 (20.0%)
95% bootstrap CI	8.6–34.3%
Programs with ≥1 constituent gene set at FDR < 0.05	7/35
Evaluable locked genes	780/830
Concordant locked genes	355/780 (45.5%)
Spearman correlation with the GSE43292 reference effects	ρ = −0.144
Correlation *p* value	5.55 × 10^−5^

Notes: Directional concordance was evaluated against the locked GSE43292 reference architecture. Program-level concordance was defined by the direction of the median NES across the fixed constituent gene sets assigned to each program, and 95% CIs were estimated from 5000 bootstrap resamples of the 35 programs. Gene-level analyses were restricted to the locked set of 830 shared leading-edge genes that were evaluable in each disease context. Spearman coefficients quantify the correspondence between context-specific log_2_ fold changes and the corresponding GSE43292 reference effects. The analysis was designed to characterize cross-dataset directional and pathway concordance rather than to provide external validation or replication.

## Data Availability

The datasets analyzed in this study are publicly available from the NCBI Gene Expression Omnibus under the accession numbers GSE39445, GSE43292, GSE28829, GSE57691, GSE56931, and GSE253903. All analysis scripts, run logs, and sessionInfo records generated in this study are available from the corresponding author upon request.

## Supplementary Materials

The following supporting information can be downloaded at https://www.mdpi.com/article/10.3390/biomedicines14091947/s1. Figure S1: Mean difference plot of gene-level expression changes in the GSE39445 sleep restriction discovery cohort; Figure S2: Mean difference plot of gene-level expression changes in paired carotid plaque and macroscopically intact arterial tissue from the GSE43292 cohort; Figure S3: Distribution of raw and Benjamini–Hochberg-adjusted *p* values in the GSE57691 aneurysm severity analysis; Figure S4: Module-size distribution across the sixteen non-grey co-expression modules and the unassigned grey category in the GSE39445 whole-blood discovery cohort; Figure S5: Comparison of primary covariate-adjusted and subject-only module–trait estimates for all sixteen non-grey co-expression modules in the GSE39445 whole-blood discovery cohort; Figure S6: Distribution of own-module membership values for the four sleep-associated co-expression modules in the GSE39445 whole-blood discovery cohort; Figure S7: Immune- and stromal-cell-associated transcriptional signals in the sleep restriction and atherosclerotic plaque cohorts; Figure S8: Cross-method and cross-cohort concordance of estimated cell-type-associated transcriptional signals in the sleep restriction and atherosclerosis cohorts; Figure S9: TF target regulatory context enrichment within the primary shared leading-edge gene program; Figure S10: Unsupervised transcriptional architecture of the GSE253903 atherosclerotic single-cell cohort; Figure S11: Marker-primary cellular annotation and orthogonal support from independent reference-based classification; Figure S12: Cluster-resolved localization of the 12 network-prioritized candidates; Figure S13: Cellular distribution of the 35 directionally concordant transcriptional programs; Figure S14: Regional eQTL and coronary-artery-disease association profiles for *SF3A3*, *SNIP1*, and *SNRPF*; Figure S15: Variant-level influence diagnostics for *SF3A3* and *SNIP1*; Figure S16: Atherosclerotic and coronary disease enrichment among the 830 locked shared leading-edge genes; Figure S17: Descriptive cross-dataset behavior of the 12 prespecified MCC-supported candidate genes; Figure S18: Detailed candidate membership across candidate-containing locked gene sets and the cellular composition of candidate–program links; Figure S19: DGIdb-based pharmacological interaction annotation of the locked sleep loss–atherosclerosis gene set; Figure S20: Identity of the positive upper 5% release-relative rule and the former three-part rule; Figure S21: BING calibration under alternative permutation-null specifications; Figure S22: Program estimability across cohorts and LINCS gene representations; Figure S23: Dependence of perturbagen-level membership on LINCS signature coverage; Figure S24: Fixed-size calibration and sensitivity analysis of LINCS gene-space behavior; Table S1: Complete gene-level differential expression results for GSE39445 comparing sleep restriction with sleep extension (19,541 gene symbol features); Table S2: Genes meeting both prespecified differential expression criteria in GSE39445 (1352 genes; 645 upregulated and 707 downregulated during sleep restriction relative to sleep extension); Table S3: Complete gene-level differential expression results for GSE43292 comparing carotid plaque with paired macroscopically intact arterial tissue (21,147 gene symbol features); Table S4: Genes meeting both prespecified differential expression criteria in GSE43292 (3719 genes; 1946 upregulated and 1773 downregulated in plaque relative to paired intact arterial tissue); Table S5: Complete gene-level differential expression results for the GSE28829 plaque-progression analysis comparing advanced with early atherosclerotic plaque (24,442 gene symbol features); Table S6: Genes meeting both prespecified differential expression criteria in GSE28829 (2218 genes; 1202 upregulated and 1016 downregulated in advanced relative to early plaque); Table S7: Summary crosswalk between gene symbol features and distinct selected-probe measurements in GSE28829 (24,442 gene symbol features corresponding to 22,807 distinct selected-probe measurements; 2218 significant gene symbol features corresponding to 2091 distinct selected-probe measurements); Table S8: Thirty genes with the largest positive and thirty genes with the largest negative log_2_ fold changes among genes meeting both prespecified differential expression criteria in the GSE28829 plaque-progression analysis; Table S9: Summary of the GSE57691 gene-level aneurysm severity analysis comparing large with small AAAs (25,235 gene symbol features; no gene met the Benjamini–Hochberg false-discovery-rate threshold); Table S10: Thirty genes with the smallest nominal *p* values in the GSE57691 aneurysm severity analysis; none met the Benjamini–Hochberg false-discovery-rate threshold; Table S11: Computational audit of the negative false-discovery-rate result in the GSE57691 aneurysm severity analysis, including alternative probe-to-gene summarization rules, variance specifications, and permutation-based assessments of the nominal gene count and moderated-*t*-statistic dispersion; Table S12: Shared measured-gene universe and differential expression overlap between GSE39445 and GSE43292 (16,869 shared measured genes; 274 genes meeting the prespecified criteria in both cohorts), including the hypergeometric overlap assessment; Table S13: Effect directions and concordance classification for the 274 genes meeting the prespecified differential expression criteria in both discovery cohorts (134 directionally concordant genes); Table S14: Complete transcriptome-wide gene set enrichment analysis results for the two discovery cohorts, including normalized enrichment scores for the 8284 gene sets evaluable in both datasets; Table S15: Ninety-one gene sets significant in both discovery cohorts, classified by directional concordance (71 concordant and 20 discordant; 45 positively enriched in both cohorts and 26 negatively enriched in both); Table S16: Program membership under the prespecified reference clustering specification based on the overlap coefficient, average linkage, and a similarity threshold of 0.50, yielding 50 redundancy-collapsed programs; Table S17: Thirty-five directionally concordant programs, including program direction, program size, median normalized enrichment scores in the sleep loss and atherosclerosis discovery cohorts, and constituent gene sets; Table S18: Sensitivity analysis across the prespecified 24-cell clustering grid comprising alternative similarity metrics, linkage methods, and similarity thresholds; Table S19: Program-level directional generalization results in the primary GSE56931 acute total sleep deprivation analysis comprising 209 samples (32 of 35 programs directionally concordant); Table S20: Program-level results from the GSE56931 matched-clock sensitivity analysis restricted to the first 24 h of sleep deprivation (28 of 35 programs directionally concordant); Table S21: Observed areas under the receiver operating characteristic curve and participant cluster bootstrap confidence intervals for the nine prespecified feature–model combinations in the GSE39445 within-cohort participant-grouped separability analysis; Table S22: Permutation analysis summary for the participant-grouped separability models, including observed and empirical-null results, fold assignments, and random-seed specifications; Table S23: Hallmark gene set variation analysis results for GSE43292 (50 Hallmark pathways; 31 Benjamini–Hochberg false-discovery-rate-significant, of which 30 showed higher inferred activity in plaque and one showed higher inferred activity in paired intact arterial tissue); Table S24: Co-expression module sizes for the weighted gene co-expression network constructed from 14,655 genes in the GSE39445 whole-blood discovery cohort using a signed network and a soft-thresholding power of 7; Table S25: Module–eigengene associations with the sleep restriction contrast for the sixteen non-grey co-expression modules in the primary covariate-adjusted linear mixed-effects analysis; positive coefficients indicate higher eigengene expression during sleep restriction; Table S26: Comparison of primary covariate-adjusted and subject-only sensitivity estimates for the four co-expression modules meeting the Benjamini–Hochberg false-discovery-rate threshold in the primary module–trait analysis; Table S27: Complete module membership correlation matrix for all 14,655 network genes across the sixteen non-grey module eigengenes, with descriptive membership *p* values and the grey eigengene-like summary retained for completeness; the grey category represents genes not assigned to a non-grey co-expression module and was excluded from biological interpretation; Table S28: Complete intramodular gene rankings for the four sleep-associated co-expression modules, ordered by signed own-module membership (kME) within each module; Table S29: Hub-gene summary for the four sleep-associated co-expression modules, listing the five genes with the highest signed own-module membership values in each module; Table S30: Complete GSE39445 MCP-counter results. Primary covariate-adjusted mixed-effects estimates, standard errors, confidence intervals, nominal *p* values, FDR values, and reduced-model sensitivity results for all ten evaluated populations; Table S31: Complete GSE39445 prespecified marker score results. Primary covariate-adjusted mixed-effects estimates, standard errors, confidence intervals, nominal *p* values, FDR values, and reduced-model sensitivity results for all ten evaluated populations; Table S32: GSE39445 cross-method concordance. Population-level comparison of MCP-counter and prespecified marker score estimates, within-cohort coefficient-direction concordance, and sample-level Spearman correlations; Table S33: Complete GSE43292 MCP-counter results. Patient-paired primary model estimates, standard errors, confidence intervals, nominal *p* values, FDR values, and corresponding unpaired sensitivity model results for all ten evaluated populations; Table S34: Complete GSE43292 prespecified marker score results. Patient-paired primary model estimates, standard errors, confidence intervals, nominal *p* values, FDR values, and corresponding unpaired sensitivity-model results for all ten evaluated populations; Table S35: GSE43292 cross-method concordance. Population-level comparison of MCP-counter and prespecified marker score estimates, coefficient-direction concordance, joint FDR status, and sample-level Spearman correlations for all ten evaluated populations; Table S36: Cross-cohort integration and marker definitions. Complete two-cohort directional-integration table for all ten populations, MCP-counter cross-cohort concordance, marker score cross-cohort concordance, four-layer directional concordance status, prespecified marker-gene membership, and dataset-specific marker measurement coverage; Table S37: Complete primary TF target regulatory context over-representation results across the three prespecified resources. Resource-specific mapped backgrounds and query sizes are reported explicitly; Table S38: FDR-significant primary TF target regulatory context findings. CollecTRI results are reported as curated regulons, whereas GTRD results are reported as regulatory target signatures; Table S39: Sensitivity of the seven primary FDR-significant findings across three prespecified input-gene definitions. No result was interpreted as direct TF binding, TF activity, causal regulation, or independent cross-resource validation; Table S40: Complete locked 830-gene shared leading-edge input used for STRING protein–protein interaction analysis; Table S41: Complete STRING functional enrichment results for the 828 mapped protein nodes; Table S42: Complete node-level network metrics and MCC scores for the STRING-derived PPI network; Table S43: Cluster-level marker-primary annotation with independent SingleR concordance; Table S44: Cluster-level marker-primary cell-type assignment and abundance; Table S45: Cell-type localization of the 830 locked Table S40 genes in GSE253903; Table S46: Cell-type localization of the 12 locked MCC-supported candidates; Table S47: Cell-type localization of the 35 fixed directionally concordant programs; Table S48: Prespecified human genetic analysis rules, locked candidate-gene exposure datasets, hg19 gene coordinates, corrected CAD metadata, and final candidate-level instrument availability; Table S49: Complete exact-cis exposure instrument audit, linkage-disequilibrium-clumped instruments, harmonized coronary artery disease associations, and instrument disposition; Table S50: Primary and sensitivity Mendelian randomization results for candidate-gene expression and coronary artery disease; Table S51: Regional colocalization status, primary posterior results, and *p*_12_-prior sensitivity for *SF3A3*, *SNIP1*, and *SNRPF*; Table S52: Shared regional eQTL–coronary artery disease summary statistics and SNP-level posterior probabilities used in the colocalization analyses; Table S53: Complete disease and phenotype over-representation results for the 830 locked shared leading-edge genes; Table S54: Prespecified cardiovascular and sleep-related disease enrichment results for the 830 locked shared leading-edge genes; Table S55: Disease-domain annotation overlap of the 12 locked MCC-supported candidate genes; Table S56: Cross-dataset directional and pathway concordance of the locked sleep loss-associated vascular transcriptional architecture; Table S57: Multilayer characterization of the 12 prespecified MCC-supported candidates within the sleep loss–atherosclerosis framework; Table S58: Candidate–program–plaque cell integrative mapping of the 12 prespecified MCC-supported candidates within the sleep loss–atherosclerosis framework; Table S59: DGIdb-based pharmacological interaction annotation of the 830 locked shared leading-edge genes and the nested set of 12 prespecified MCC-supported candidates; Table S60: Descriptive, release-relative LINCS perturbation-signature prioritization for the locked shared sleep loss–atherosclerosis gene set; Table S61: Extended fixed-size calibration of the 68-feature LINCS gene space against random and constrained matched comparator subsets; Table S62: Sensitivity of the GSE39445 discovery analysis to the representative probe selection rule.

## Abbreviations

The following abbreviations are used in this manuscript:

AAA	Abdominal aortic aneurysm
AUC	Area under the receiver operating characteristic curve
BH	Benjamini–Hochberg
BING	Best inferred genes
CAD	Coronary artery disease
CI	Confidence interval
FDR	False discovery rate
GEO	Gene Expression Omnibus
GO	Gene Ontology
GO-BP	Gene Ontology Biological Process
GO-CC	Gene Ontology Cellular Component
GO-MF	Gene Ontology Molecular Function
GSEA	Gene set enrichment analysis
GSM	GEO sample
GSVA	Gene set variation analysis
GTRD	Gene Transcription Regulation Database
kME	Module membership
LASSO	Least absolute shrinkage and selection operator
LINCS	Library of Integrated Network-Based Cellular Signatures
MCC	Maximal clique centrality
MCP-counter	Microenvironment Cell Populations-counter
MSigDB	Molecular Signatures Database
NES	Normalized enrichment score
PPI	Protein–protein interaction
RPCA	Reciprocal principal component analysis
scRNA-seq	Single-cell RNA sequencing
SNN	Shared nearest neighbor
STRING	Search Tool for the Retrieval of Interacting Genes/Proteins
TF	Transcription factor
UMAP	Uniform manifold approximation and projection
WGCNA	Weighted gene co-expression network analysis
