# Supplementary material for: Integrative Systems Genomics and Pharmacology Define Shared Immune–Vascular Programs and Prioritize an Experimentally Testable Candidate Linking Sleep Loss to Human Atherosclerosis
Source: Biomedicines. 2026 Aug 29;14(9):1947. doi: 10.3390/biomedicines14091947 (PMC13604422; doi:10.3390/biomedicines14091947)
Supplement: Supplementary file 1 [file biomedicines-14-01947-s001.zip › Supplementary materials.pdf]

Supplementary Materials

Integrative Systems Genomics and Pharmacology Define Shared Immune–Vascular Programs and Prioritize an Experimentally Testable Candidate Linking Sleep Loss to Human Atherosclerosis

Overview

This Supplementary Materials document provides detailed methodological specifications for selected secondary analyses, together with the supplementary figures and the captions accompanying the complete supplementary table set for the study. Supplementary Methods S1 and S2 provide the extended technical specifications for the within-cohort participant-grouped separability analysis and the LINCS perturbation-signature calibration and sensitivity analyses, respectively. Figures S1–S24 document the principal diagnostic and sensitivity analyses supporting the transcriptomic, network, cellular, genetic, and pharmacological components of the study. Captions for Tables S1–S62 are provided here, whereas the complete supplementary tables and underlying numerical results are presented separately in the Supplementary Tables workbook. The LINCS supplementary analyses distinguish perturbagen prioritization from gene-space calibration. Figures S20–S23 and Table S60 document the release-relative perturbation-signature framework, calibration diagnostics, effective dimensionality, program estimability, and signature-coverage dependence; Figure S24 and Table S61 extend this calibration at a fixed nominal feature count using random and constrained matched 68-feature comparator subsets, block-granularity sensitivity, and random-query calibration. Table S62 reports a contrast-independent maximum-IQR representative-probe sensitivity analysis for GSE39445, comparing gene-level, gene-set-level, and program-level results with the submitted specification while retaining the GSE43292 arm and the prespecified 50-program architecture unchanged. Because the constrained matched subsets exhibited substantial between-set dependency and reduced effective dimensionality, this arm is retained as a constrained comparator and is not interpreted as an isolated test of gene composition. Collectively, these supplementary analyses provide methodological detail, analytical provenance, and sensitivity characterization while preserving the inferential boundaries of the main analysis; they do not redefine the locked transcriptional architecture, candidate set, perturbagen membership, compound prioritization, or therapeutic interpretation reported in the main manuscript.

Supplementary Methods

S1. Detailed methods for the within-cohort participant-grouped separability analysis

The within-cohort separability analysis in GSE39445 evaluated three prespecified feature representations: the 35 redundancy-collapsed program scores, the 71 individual concordant gene-set scores, and the 830 genes belonging to the shared leading-edge intersection of the concordant pathways identified across the two discovery cohorts. Program scores were calculated for each sample as the median of the GSVA scores of the constituent gene sets assigned to that program. Within this secondary separability analysis, the prespecified reference feature–model combination was LASSO logistic regression applied to the 35 program scores. Random forest and radial-kernel support vector machine models were evaluated as secondary approaches. For random forest, the number of trees was fixed at 500, and the number of variables considered at each split was set to the square root of the number of input features. For the support vector

machine, the parameters were fixed at cost = 1 and gamma = 1 divided by the number of input features. No outcome-dependent hyperparameter search was performed for either secondary model.

Model evaluation used five-fold participant-grouped cross-validation repeated five times. Within each repeat, all samples from a given participant were assigned to the same outer fold, ensuring that no participant contributed observations to both the training and test partitions. For LASSO, the penalty parameter was selected within each outer training set using participant-grouped inner cross-validation. Outer- and inner-fold assignments, random seeds, and model parameters were fixed and recorded before model evaluation. Discriminative performance was summarized using the area under the receiver operating characteristic curve (AUC). Two descriptive summaries were retained: the AUC obtained from a fixed reference repeat and the mean AUC across the five repeated outer cross-validations. An additional AUC was calculated from the sample-wise mean of the five out-of-fold predictions.

Two complementary inferential procedures were specified and reported separately. First, uncertainty in the observed AUC was estimated using 2,000 cluster-bootstrap resamples, with participant as the resampling unit. Confidence intervals were calculated for both the reference-repeat out-of-fold predictions and the sample-wise mean out-of-fold predictions. These intervals were used to assess whether the uncertainty range included the null AUC value of 0.5. Second, the reference-repeat AUC was compared with a matched empirical null distribution generated by refitting the complete modeling pipeline after participant-blocked label swapping. For each participant, all condition labels were either retained or reversed jointly, thereby preserving the participant-level dependence structure. The same outer folds, inner-fold assignments, feature representation, and model specification were used for the observed and permuted analyses. The reference program–LASSO analysis used 1,000 permutations, whereas each secondary feature–model combination used 100 permutations. One-sided empirical p values were calculated using the finite-permutation correction described by Phipson and Smyth [32], by adding one to both the number of permuted AUC values equal to or greater than the observed AUC and the total number of permutations.

Participant-blocked label swapping did not constrain the total number of samples assigned to each permuted class to match the observed 221:217 distribution because four participants contributed samples under only one protocol. The resulting empirical null was therefore interpreted as the null distribution induced by the observed crossover and incomplete-crossover structure rather than as a permutation distribution conditioned on fixed class totals. As a sensitivity analysis, GSVA scores were recomputed separately within the training and test partitions of each fold. Because the resulting training and test scores were referenced to different sample distributions, this procedure was not characterized as a leakage-free validation analysis. Its purpose was limited to assessing whether the overall interpretation was materially affected by the sample population used as the reference for GSVA scoring.

S2. Detailed LINCS perturbation-signature calibration and sensitivity procedures

The LINCS perturbation-signature analysis used the release-matched Level 5 moderated z-score matrix from GSE70138, comprising 118,050 signatures across 12,328 measured or inferred genes, together with the corresponding gene, signature, perturbagen, and cell-line metadata. File identity was verified against the depositor-provided checksum before analysis. The analytical universe was restricted a priori to signatures annotated as small-molecule perturbations (trt_cp). No additional filtering was applied according to cell

line, dose, exposure duration, signature-quality metric, or observed reversal score, and signatures from different LINCS releases were not pooled.

The cohort-specific query vectors comprised the locked differential-expression statistics from GSE39445 and GSE43292 restricted to the prespecified 830-gene shared leading-edge set. Gene symbols were mapped deterministically to release-matched L1000 probe identifiers. Where both directly measured landmark and inferred representations were available for the same gene, the landmark representation was prioritized; genes unavailable in the selected representation were neither substituted nor imputed. Of the 830 locked genes, 622 were represented in the best-inferred gene (BING) space, comprising 68 directly measured landmark genes and 554 inferred genes. An additional 46 genes were represented only in the extended inferred space, whereas 162 were unavailable in the platform representation used for the analysis. The 622-feature BING representation and its locked 68-feature landmark subset were analyzed separately and were not merged.

For each eligible LINCS signature, the reversal score was calculated as the negative Spearman rank correlation between the cohort differential-expression vector and the perturbation-signature vector across genes represented in both the query and the relevant LINCS gene space. Computationally, this was implemented using column-wise unit-norm standardized ranks, which is algebraically equivalent to the negative Spearman correlation coefficient. Signatures were scored only when at least 20 query genes were represented, and estimability status was retained for every signature–query combination.

Signature-level descriptive membership required a positive reversal score together with placement within the upper 5% of the corresponding scored release-relative distribution. Perturbagen-level membership required at least one qualifying signature in each primary cohort. Program-level context was evaluated separately against the locked transcriptional-program architecture. Within each cohort, a perturbagen was assigned context for a program when the median program-level reversal score was positive and at least one program-level signature was both positive and within the upper 5% of the relevant release-relative distribution. Shared program context required the same locked program to satisfy these conditions in both cohorts. Program estimability required at least five represented program genes in the BING space and at least three in the landmark subset. The landmark subset was evaluated using the same directional and percentile definitions but was not used to determine primary perturbagen membership.

An earlier formulation of the signature-level rule additionally required a pooled permutation Benjamini–Hochberg-adjusted q value < 0.05. Calibration analyses were therefore undertaken to determine whether this criterion contributed information beyond the positive-score and upper-5% requirements. Within the release and scoring framework used here, the pooled q criterion did not define an additional membership layer. Null behavior was further characterized using a fixed calibration family of 5,000 signatures, 2,000 permutations, and a disjoint 1,000-signature reference family for construction of correlation-informed gene blocks. Block granularity was harmonized across gene spaces at approximately seven genes per block. Three calibration specifications were examined: a pooled gene-label permutation null, a signature-specific unrestricted gene-label permutation null, and a signature-specific correlation-block permutation null. Because unrestricted gene-label permutation assumes exchangeability among measurements, whereas most BING features are deterministic functions of the measured landmark space, the unrestricted null does not reproduce the dependency structure of the BING representation. Permutation-derived q values were therefore retained as calibration annotations and were not used to determine perturbagen membership.

Effective dimensionality was quantified from the eigenvalue spectrum of the gene-correlation matrix using the participation ratio [71], estimated from the disjoint reference family. Participation ratio and calibration

dispersion were treated as distinct quantities: the former summarized covariance-related redundancy in the represented gene space, whereas the latter described the width of the observed query-specific reversal-score distribution relative to its corresponding permutation null. Neither quantity was interpreted as an estimate of the number of statistically independent tests.

To assess the behavior of the locked 68-feature landmark representation at a common nominal feature count, an extended calibration analysis compared it with 50 random 68-feature subsets sampled from the 622-feature BING representation and 50 constrained matched 68-feature inferred subsets. The locked cohort-specific query definitions and reversal-scoring procedure were retained throughout. For each comparator set, reversal-score dispersion was summarized by the standard deviation of the observed reversal scores, and effective dimensionality was quantified by the participation ratio derived from the common disjoint reference family. The locked landmark value was located relative to the empirical 2.5th–97.5th percentile distribution of each 50-set comparator arm; these empirical ranges were used descriptively and were not interpreted as confidence intervals.

Additional sensitivity analyses evaluated the dependence of blockwise permutation results on target block sizes of 4, 7, and 14 genes and examined random-query calibration across 20 random queries in each gene space. Set diversity, between-set overlap, and effective dimensionality were retained explicitly in interpreting the constrained matched arm, which was therefore treated as a constrained comparator rather than as a definitive isolation of gene-composition effects. These calibration and sensitivity analyses were used solely to characterize the statistical behavior and robustness of the LINCS gene spaces and did not alter perturbagen membership, program-context assignment, compound prioritization, or therapeutic interpretation.

Cross-cohort dependence and release-coverage dependence were evaluated as described in the main Methods. Perturbagen-level median BING reversal scores were examined across the two primary cohorts, and perturbagen membership was additionally evaluated in relation to the number of available signatures per perturbagen. No coverage-adjusted ranking, weighting procedure, corrective threshold, or post hoc modification of perturbagen membership was applied. Detailed calibration, coverage, dependence, and perturbagen-level outputs are provided in Tables S60 and S61 and the corresponding Supplementary Figures.

Analyses were performed in R 4.6.0. LINCS matrix files were accessed using cmapR 1.24.0 and rhdf5 2.56.0, and tabular processing was performed using data.table 1.18.4. Primary analytical inputs were identity-checked before analysis. Derived tables and final figures were generated within a versioned workflow recording the script version, run identifier, input and output paths, numerical consistency checks, and output-file provenance. Final figure production used locked derived tables; no Stage B or Stage C scientific result was reconstructed during figure generation.

Supplementary Figures

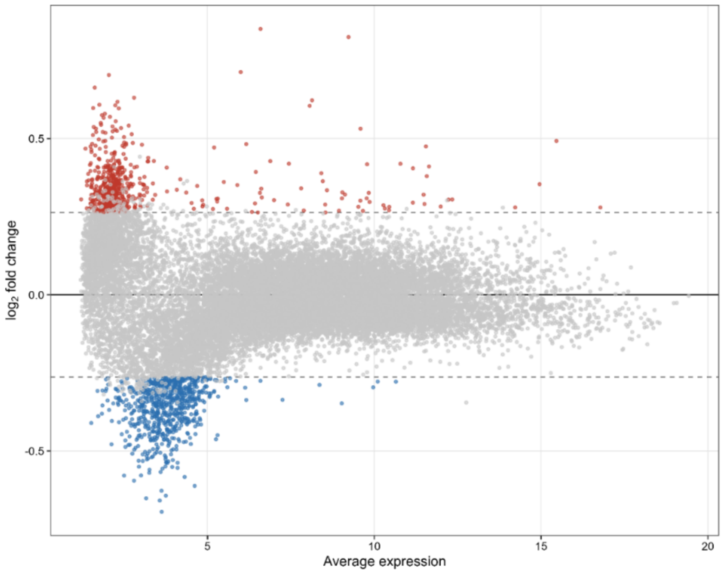

Figure S1. Mean–difference plot of gene-level expression changes in the GSE39445 sleep-restriction discovery cohort. Each point represents one gene symbol retained after deterministic probe-to-gene summarization. The x-axis shows average expression, and the y-axis shows the estimated $\log_2$ fold change for sleep restriction relative to sleep extension. Positive values indicate higher expression during sleep restriction, whereas negative values indicate higher expression during sleep extension. Genes meeting both prespecified criteria—a Benjamini–Hochberg false-discovery-rate–adjusted p value below 0.05 and an absolute $\log_2$ fold change greater than $\log_2(1.2)$—are distinguished according to effect direction. The horizontal dashed lines indicate the prespecified effect-size threshold.

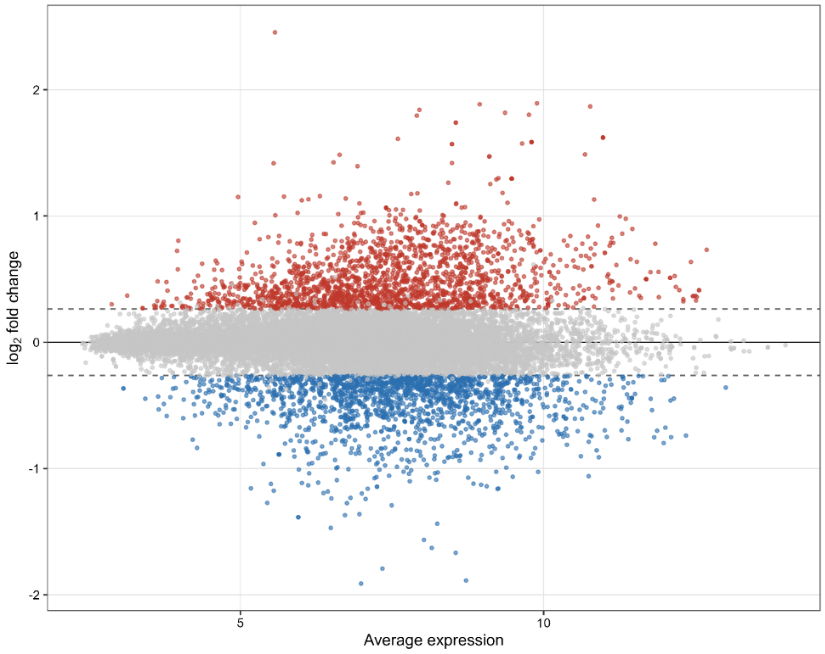

Figure S2. Mean–difference plot of gene-level expression changes in paired carotid plaque and macroscopically intact arterial tissue from the GSE43292 cohort. Each point represents one gene-symbol feature retained after expansion of composite platform annotations and deterministic gene-level summarization. The x-axis shows average expression, and the y-axis shows the estimated log_2 fold change for carotid plaque relative to paired intact arterial tissue. Positive values indicate higher expression in plaque, whereas negative values indicate higher expression in intact arterial tissue. Genes meeting both prespecified criteria—a Benjamini–Hochberg false-discovery-rate–adjusted p value below 0.05 and an absolute log_2 fold change greater than $log_2(1.2)$—are distinguished according to effect direction. The horizontal dashed lines indicate the prespecified effect-size threshold.

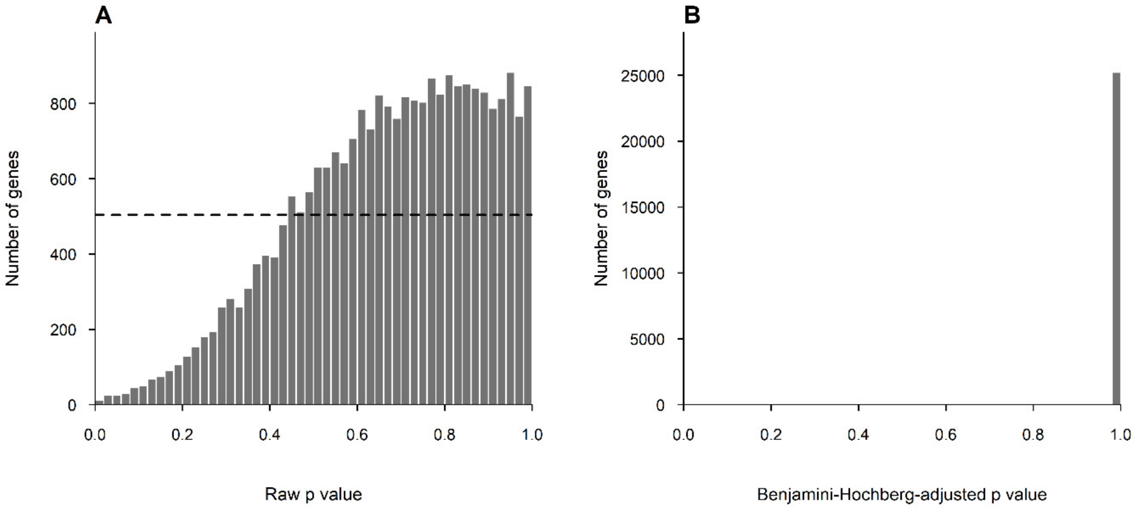

Figure S3. Distribution of raw and Benjamini–Hochberg-adjusted p values in the GSE57691 aneurysm-severity analysis. (A) Histogram of raw p values obtained from the primary comparison of large versus small abdominal aortic aneurysms. The horizontal dashed line indicates the expected number of genes per bin under a uniform raw p-value distribution. (B) Histogram of Benjamini–Hochberg-adjusted p values from the same analysis. The concentration of adjusted p values near 1 is consistent with the absence of genes meeting the prespecified false-discovery-rate threshold. These distributions are presented as descriptive diagnostics and do not establish biological equivalence between the two aneurysm groups.

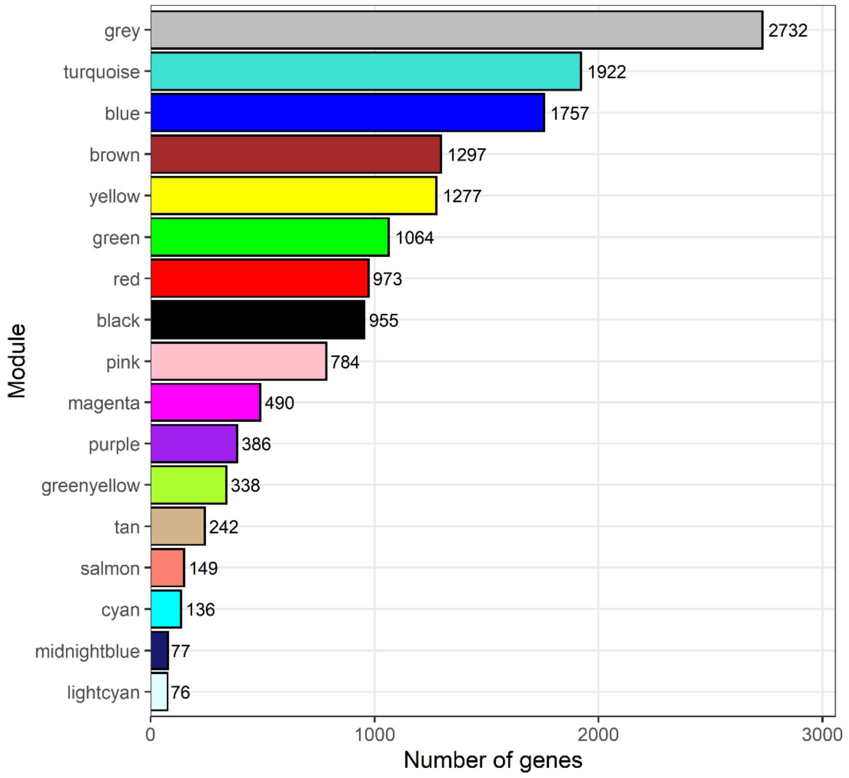

Figure S4. Module-size distribution across the weighted gene co-expression network constructed from the GSE39445 whole-blood discovery cohort. Bars show the number of genes assigned to each of the sixteen non-grey co-expression modules and to the unassigned grey category. Module colors correspond to the final WGCNA module labels. The grey category contains genes that were not assigned to a non-grey module and was retained for completeness but excluded from biological interpretation.

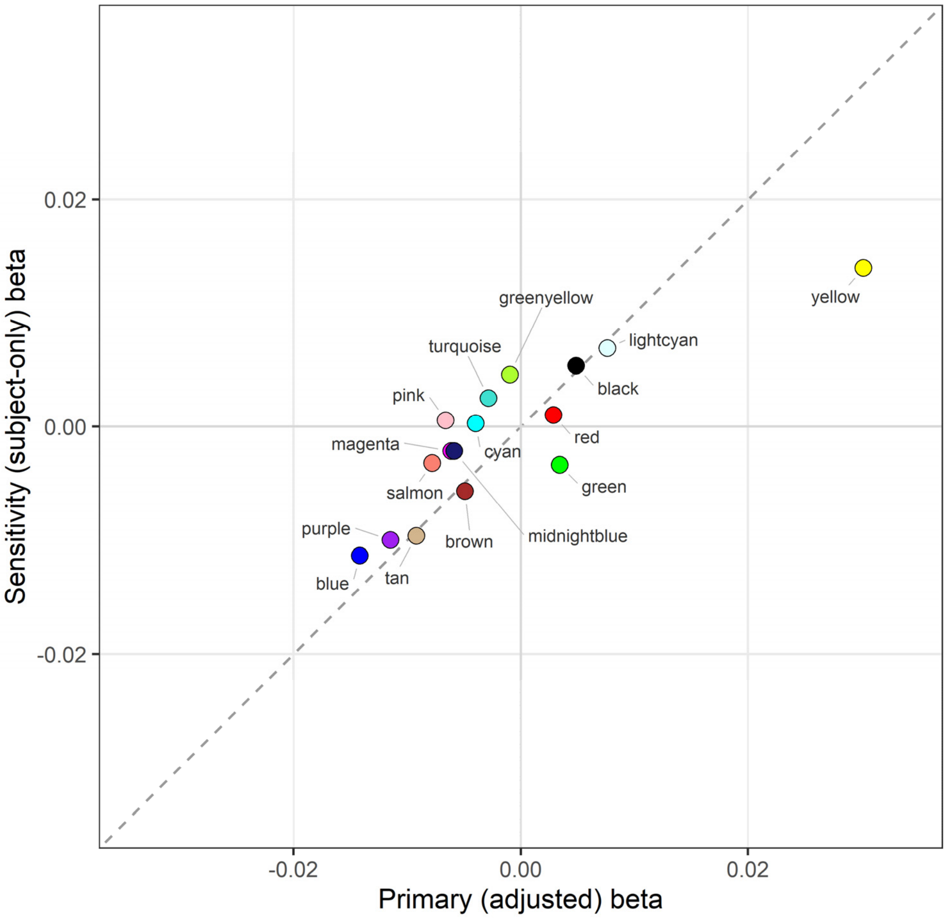

Figure S5. Comparison of primary covariate-adjusted and subject-only sensitivity estimates for module–trait associations in the GSE39445 whole-blood discovery cohort. Each point represents one of the sixteen non-grey co-expression modules. The x-axis shows the estimated sleep-restriction coefficient from the primary linear mixed-effects model adjusted for hours awake and circadian phase, whereas the y-axis shows the corresponding coefficient from the subject-only sensitivity model. Positive coefficients indicate higher module eigengene expression during sleep restriction, and negative coefficients indicate higher expression during sleep extension. The dashed diagonal line denotes equality between the two estimates. Modules lying close to this line show similar effect estimates across the two model specifications.

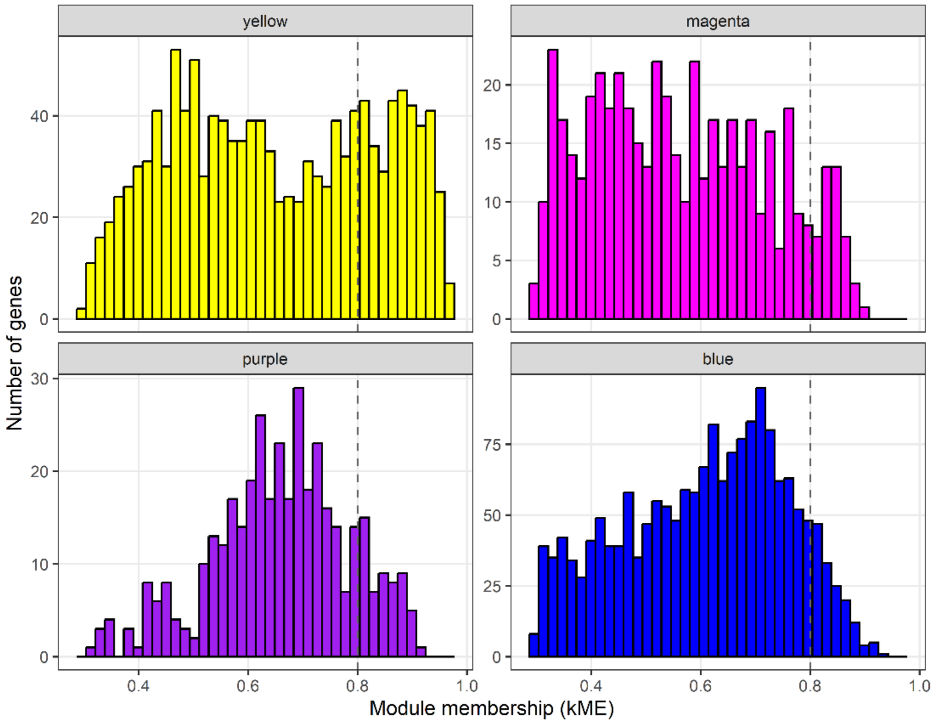

Figure S6. Distribution of own-module membership values for the sleep-associated co-expression modules identified in the GSE39445 whole-blood discovery cohort. Each panel shows the distribution of signed own-module membership values (kME) for genes assigned to one of the four modules that met the Benjamini–Hochberg false-discovery-rate threshold in the primary module–trait analysis. Higher kME values indicate stronger correlation between a gene's expression profile and the corresponding module eigengene. The vertical dashed line marks the prespecified kME = 0.80 threshold used to identify high-membership intramodular candidate genes. The yellow module is shown for completeness but was excluded from biological interpretation because its low-expression, tissue-restricted gene composition was considered more consistent with technical signal than with a coherent whole-blood program.

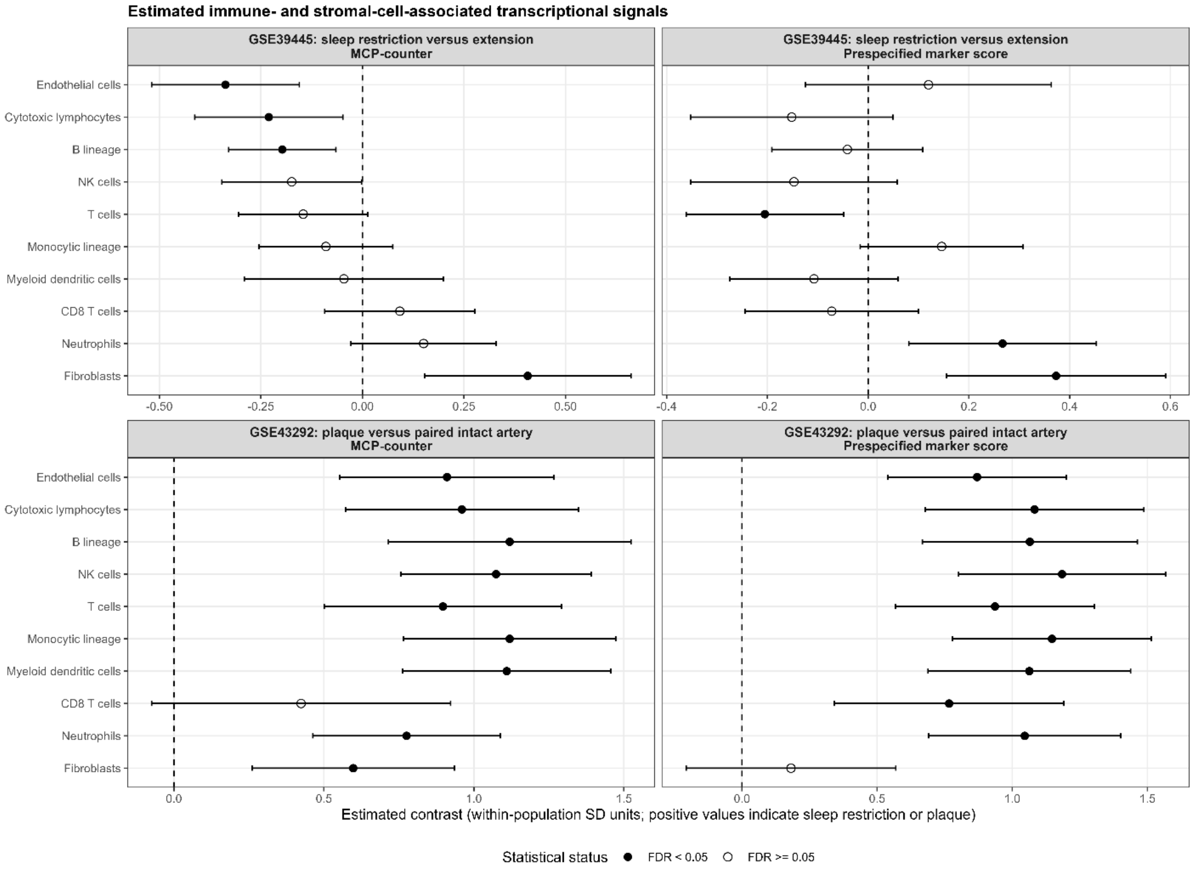

Figure S7. Immune- and stromal-cell-associated transcriptional signals in the sleep-restriction and atherosclerotic-plaque cohorts. Forest plots show primary-model estimates obtained using MCP-counter and the prespecified marker-score approach. GSE39445 coefficients represent sleep restriction relative to sleep extension, whereas GSE43292 coefficients represent carotid atherosclerotic plaque relative to patient-matched macroscopically intact arterial tissue. Points denote estimated differences in within-population standard-deviation units, and horizontal lines denote 95% confidence intervals. Filled points indicate FDR < 0.05, whereas open points indicate estimates that did not meet this threshold. The dashed vertical line denotes no estimated difference. Positive coefficients indicate higher scores during sleep restriction or in plaque, according to the corresponding panel. These scores represent transcriptome-derived cell-associated signals and should not be interpreted as direct estimates of cell fractions, absolute cell numbers, or histological infiltration.

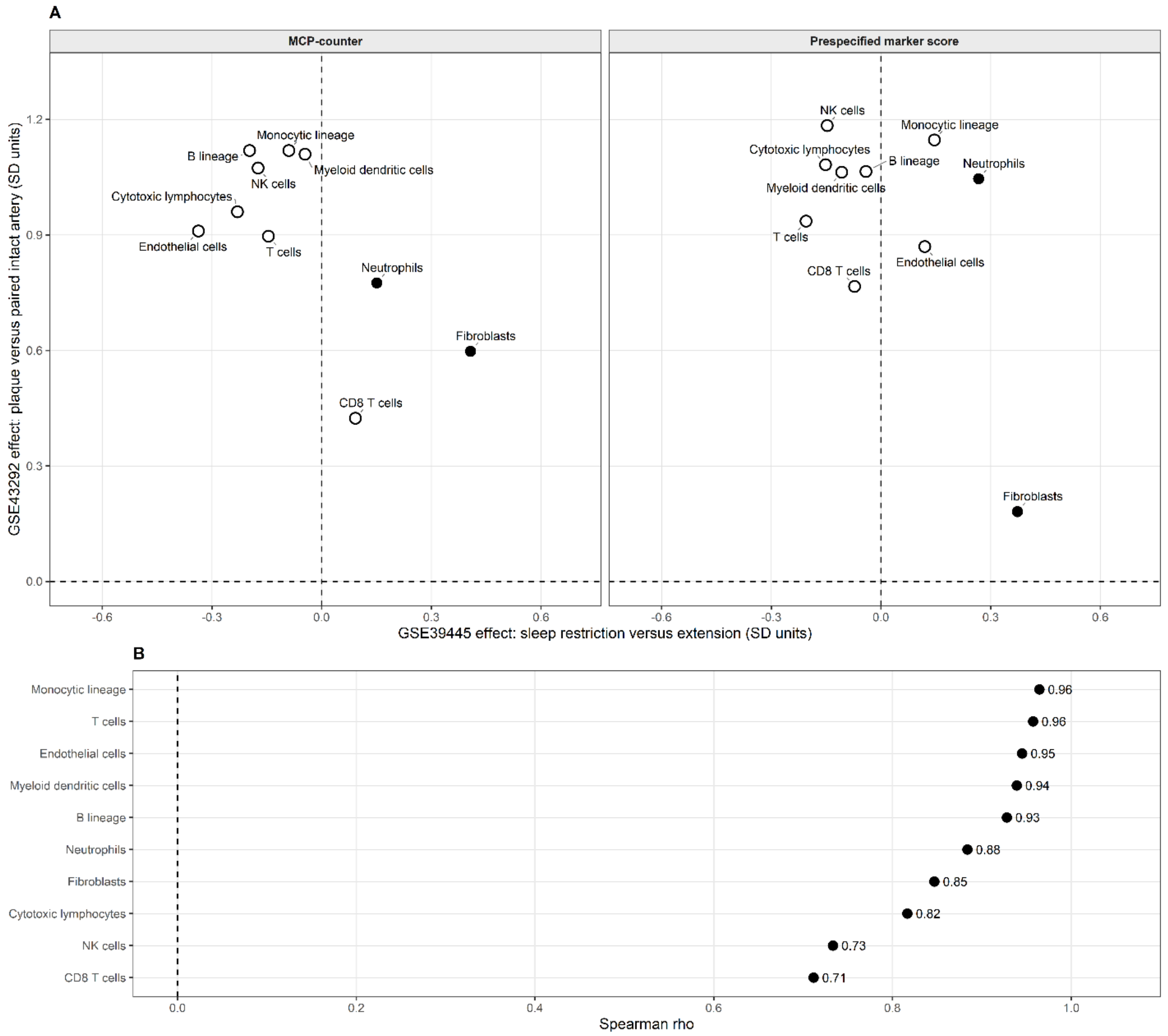

Figure S8. Cross-method and cross-cohort concordance of estimated cell-type-associated transcriptional signals in the sleep-restriction and atherosclerosis cohorts. (A) Cohort-level effect estimates are compared between GSE39445, representing sleep restriction relative to sleep extension, and GSE43292, representing carotid plaque relative to patient-matched intact arterial tissue, separately for MCP-counter and the prespecified marker-score approach. Filled points indicate neutrophils and fibroblasts, the two populations for which effect direction was concordant across all four cohort-by-method combinations; open points indicate the remaining populations. Dashed horizontal and vertical lines denote zero effect. (B) Sample-level Spearman rank correlations between MCP-counter and prespecified marker scores in GSE43292. All ten correlations met the Benjamini–Hochberg false-discovery-rate threshold. Because the two approaches draw on partially overlapping marker genes, the comparison assesses directional and rank consistency rather than equality of score magnitude or independent methodological validation.

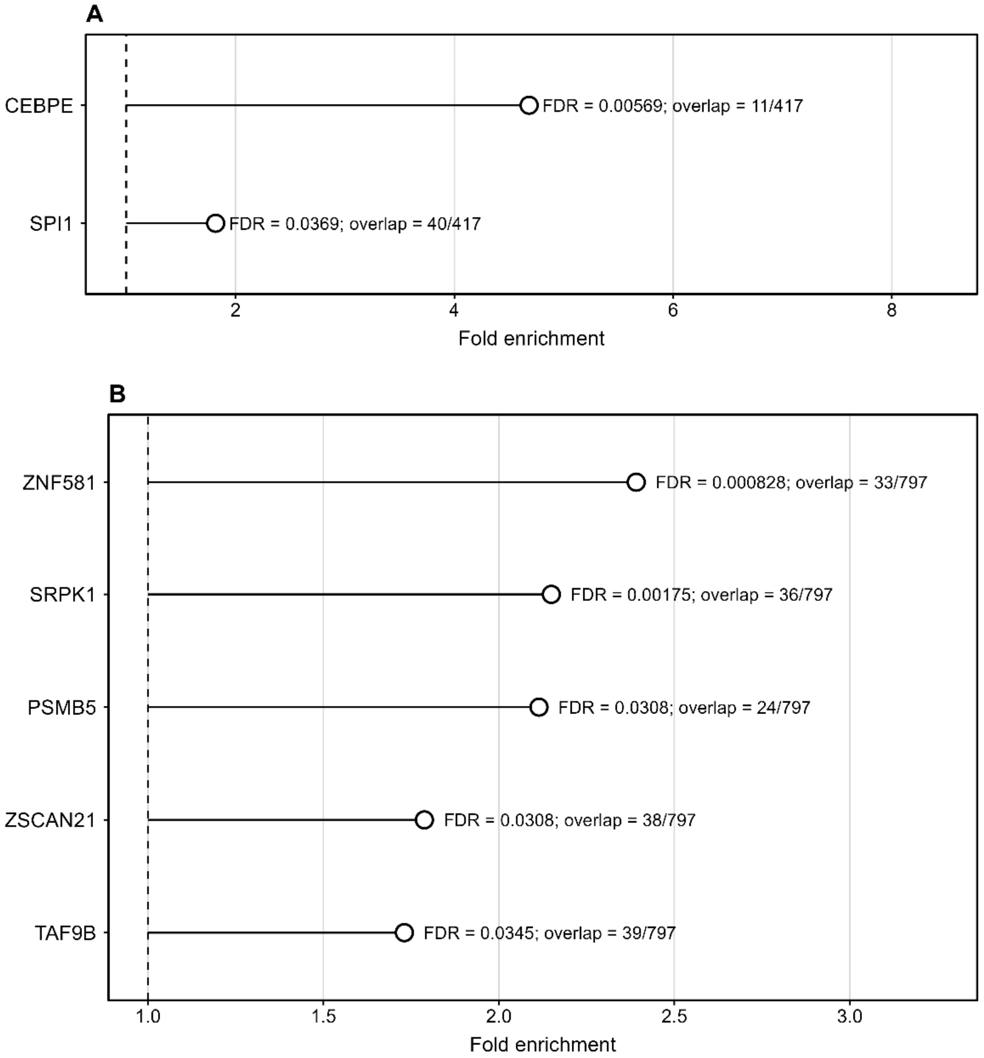

Figure S9. TF–target regulatory-context enrichment within the primary shared leading-edge gene program. (A) FDR-significant regulons identified using the frozen static human CollecTRI network. (B) FDR-significant regulatory target signatures identified using the MSigDB C3:TFT:GTRD collection. Only findings meeting the prespecified Benjamini–Hochberg false-discovery-rate threshold of 0.05 in the primary analysis are shown. Point position indicates fold enrichment relative to the corresponding resource-specific analytical background. Text annotations report the Benjamini–Hochberg-adjusted *P* value (FDR) and the number of overlapping genes relative to the resource-mapped primary query size. The primary input comprised 830 genes before resource-specific mapping, of which 417 were represented in CollecTRI and 797 in the GTRD collection. The two panels use resource-specific x-axis ranges; therefore, visual distances and fold-enrichment magnitudes should not be compared directly across panels. GTRD set names are reported as regulatory target-signature labels and do not establish that every corresponding named entity functioned as an active DNA-binding transcription factor. No finding achieved FDR-significant support in more than one independent TF–target resource. Accordingly, the results indicate source-specific regulatory context and should not be interpreted as evidence of direct TF binding, TF activation or inhibition, causal regulation, independent regulatory validation, or therapeutic efficacy.

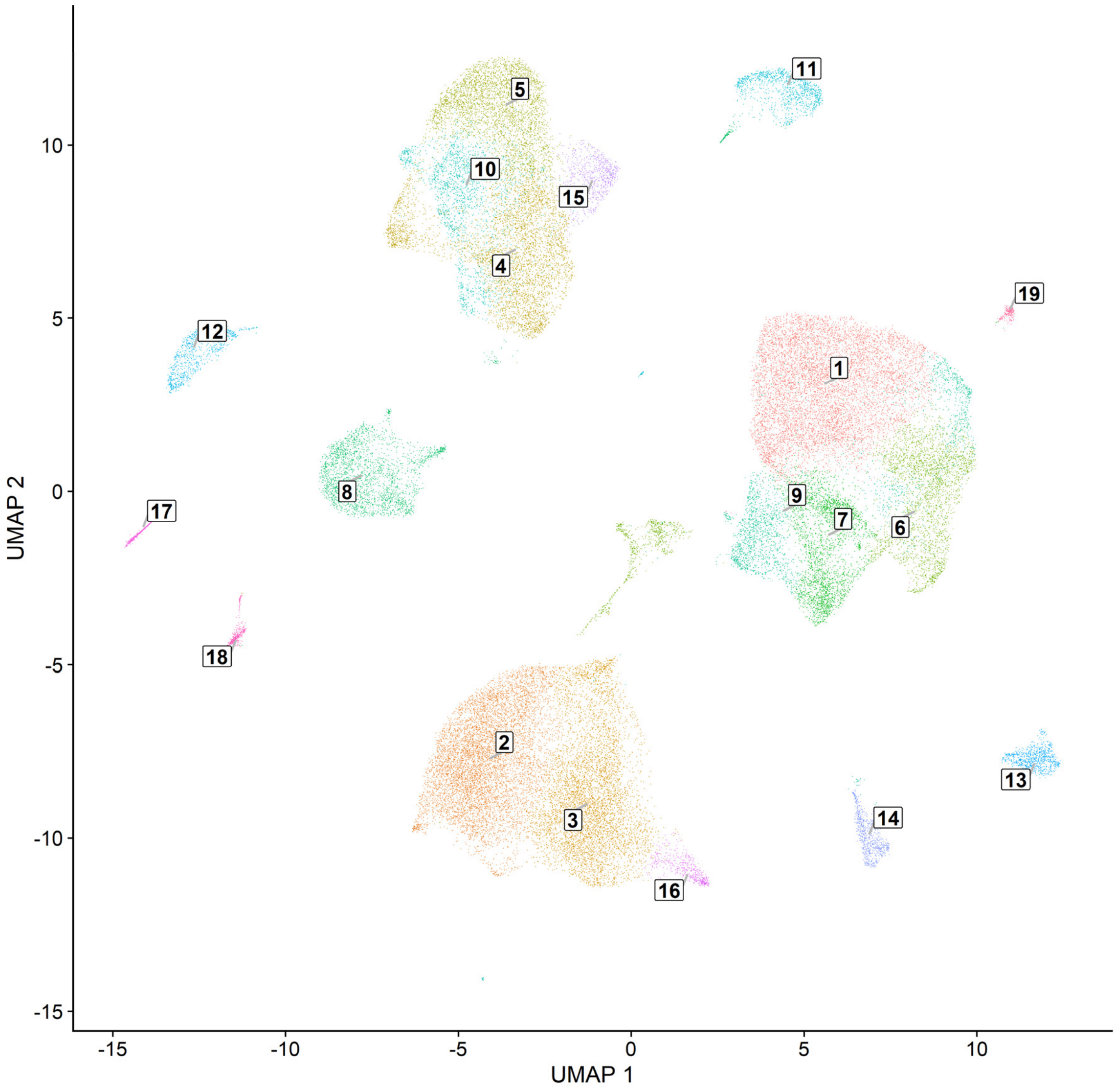

Figure S10. Unsupervised transcriptional architecture of the GSE253903 atherosclerotic single-cell cohort. The fixed RPCA-integrated UMAP embedding displays 49,188 cells partitioned into 19 Leiden clusters. Numbers indicate the corresponding Leiden cluster identifiers and do not represent cell-type labels. This unsupervised cluster map defines the transcriptional framework on which subsequent marker-primary annotation and candidate-localization analyses were based. In contrast to the cell-type representation shown in Figure 8, this figure preserves the underlying cluster resolution and documents intratissue cellular heterogeneity before biological consolidation into broader vascular, stromal, and immune compartments.

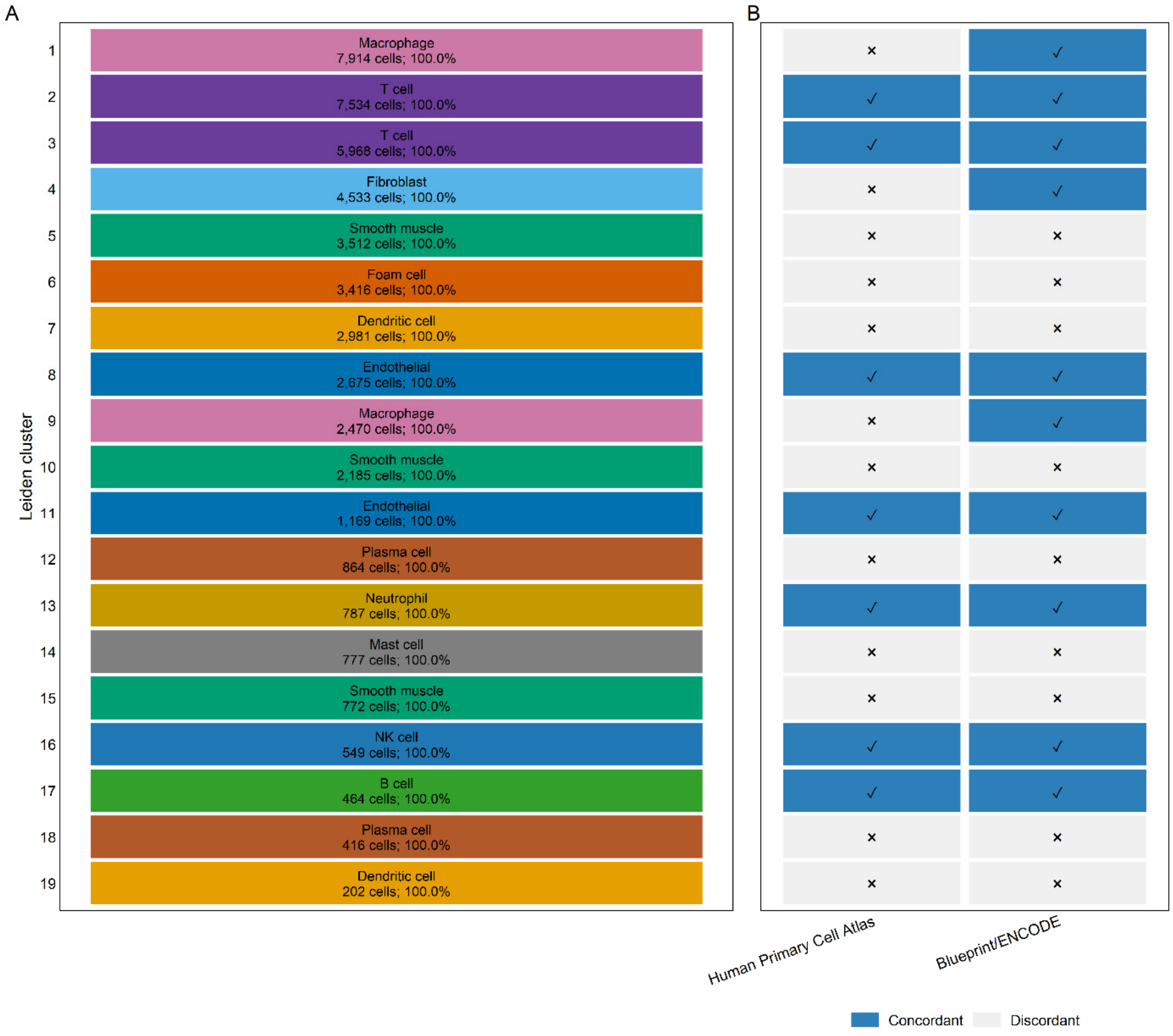

Figure S11. Marker-primary cellular annotation and orthogonal support from independent reference-based classification. (A) Dominant marker-primary cell-type assignment across the 19 Leiden clusters. Each cluster is represented by its principal cellular identity together with the corresponding cell count and within-cluster proportion, illustrating the quantitative relationship between the unsupervised cluster structure and the final biological annotation. (B) Concordance of the marker-primary annotations with independent SingleR classifications based on the Human Primary Cell Atlas and Blueprint/ENCODE reference panels. Check marks indicate concordant assignments, whereas multiplication signs indicate discordance. The reference-based classifications were used as an orthogonal appraisal of annotation robustness and did not supersede the marker-primary framework or induce post hoc reassignment.

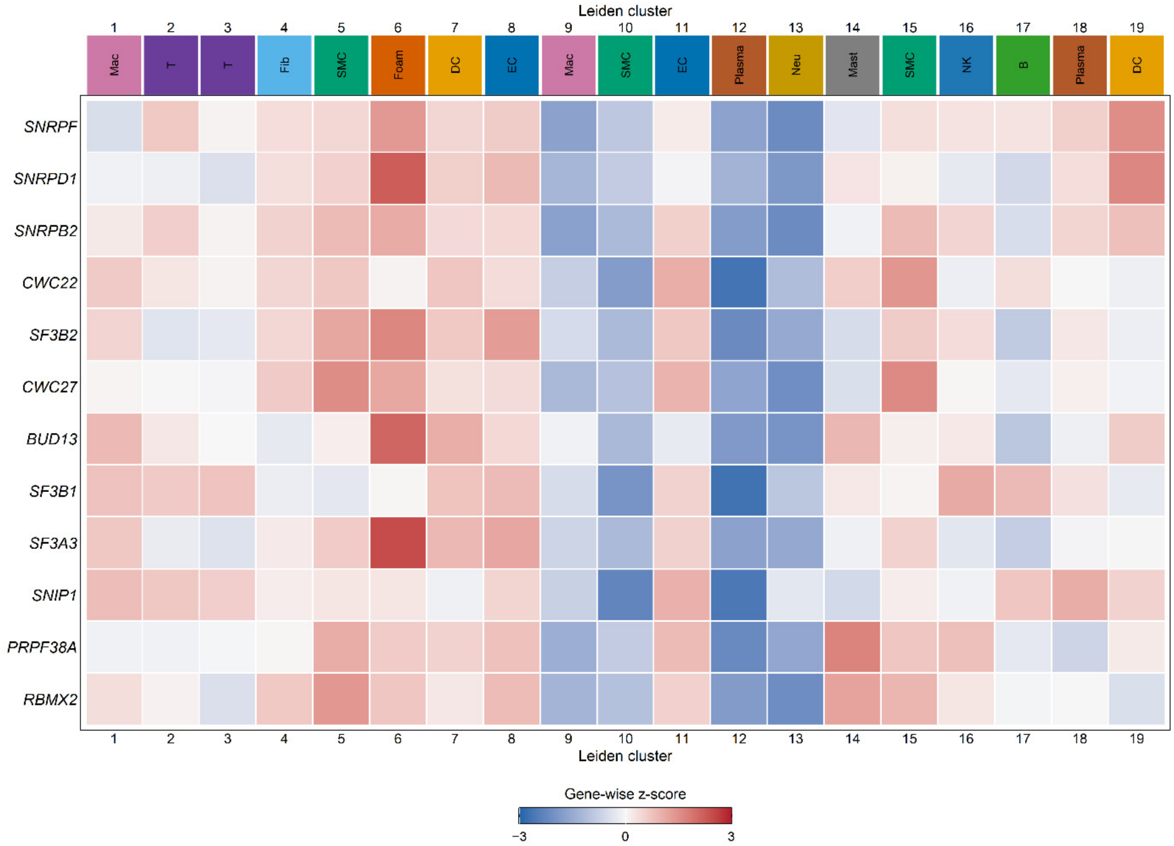

Figure S12. Cluster-resolved localization of the 12 network-prioritized candidates. The upper annotation strip identifies the dominant marker-primary cell type associated with each of the 19 Leiden clusters. The heatmap depicts gene-wise standardized average log-normalized expression of the 12 prespecified Maximal Clique Centrality-supported candidates across the cluster landscape; positive and negative z-scores denote relative enrichment and relative depletion, respectively, for each candidate. By resolving candidate expression below the broader cell-type level, this analysis distinguishes compartment-wide signals from cluster-restricted expression patterns and provides a higher-resolution complement to the cellular localization presented in Figure 8. Candidate identity, ranking, and display order were fixed before interrogation of the single-cell dataset.

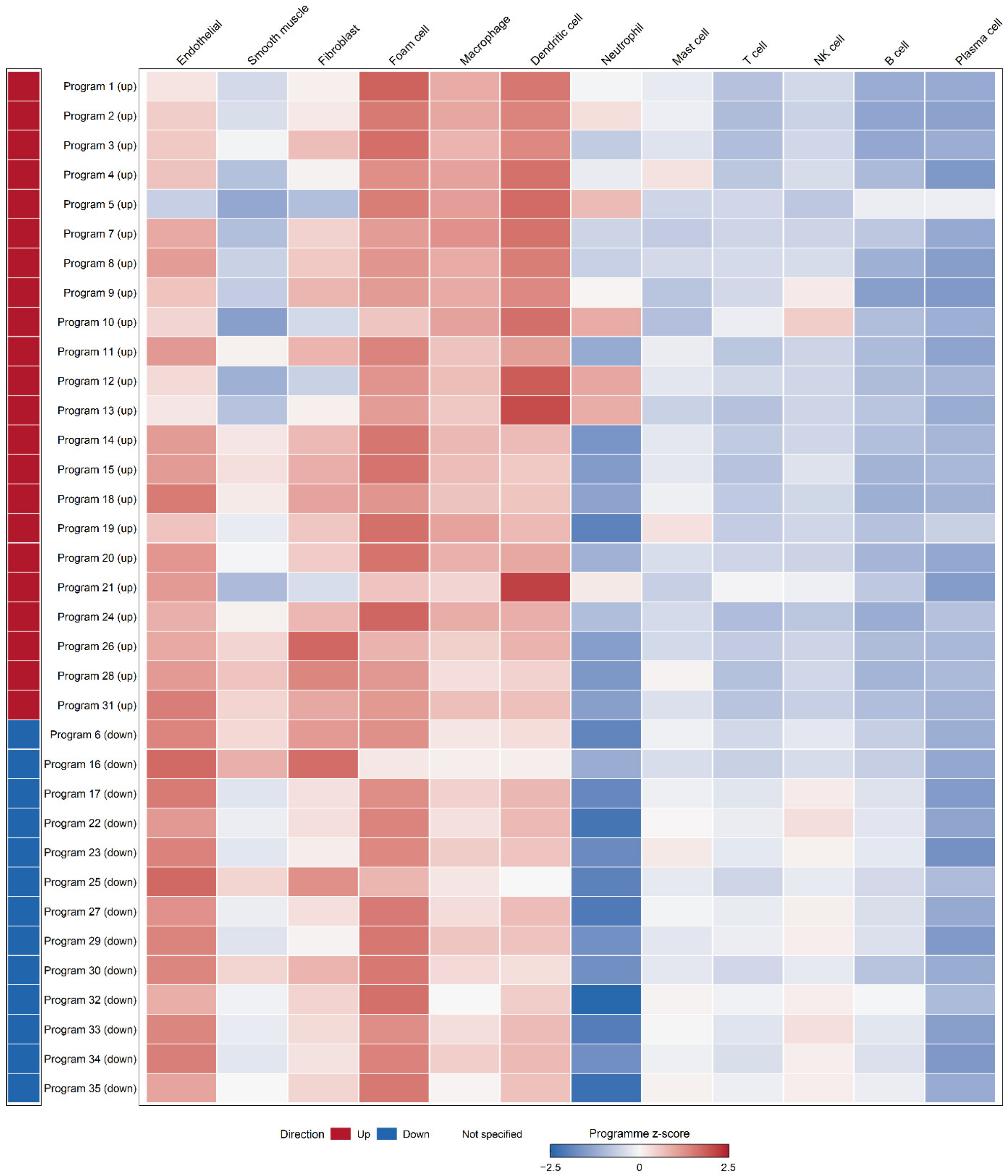

Figure S13. Cellular distribution of the 35 directionally concordant transcriptional programs. The heatmap depicts program-wise standardized scores across the 12 marker-primary cell types, extending the analysis from individual network-prioritized candidates to coordinated transcriptional modules. The 35 programs represent the prespecified directionally concordant set carried forward from the preceding integrative analysis and were not redefined using GSE253903. Their differential localization across vascular, stromal, and immune compartments provides a systems-level view of the cellular contexts in which the shared

sleep–atherosclerosis transcriptional architecture is expressed. These patterns should be interpreted as systems-level cellular contextualization rather than as independent pharmacological validation, evidence of causality, or confirmation of therapeutic efficacy.

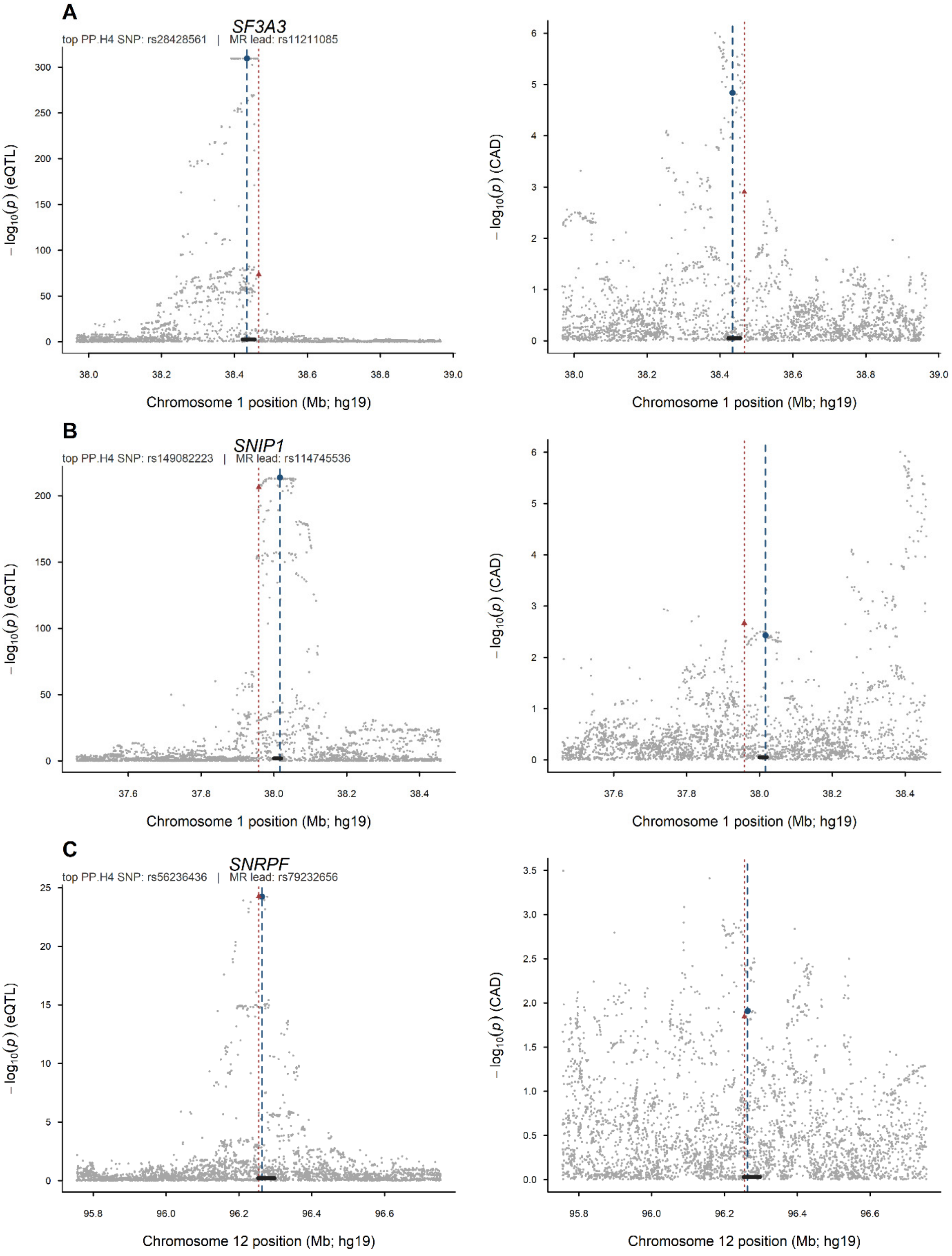

Figure S14. Regional eQTL and coronary-artery-disease association profiles for *SF3A3*, *SNIP1*, and *SNRPF*. Rows (A–C) correspond to *SF3A3*, *SNIP1*, and *SNRPF*, respectively; the left panel in each row shows the

eQTL association profile and the right panel shows the coronary artery disease association profile. Blue dashed lines mark the variant with the highest SNP-level PP.H4 in the corresponding colocalization analysis, whereas red dotted lines mark the lead Mendelian-randomization instrument used to define the regional query. Gene boundaries are shown as horizontal black segments. Track-specific y-axis scales were retained because the magnitude of association evidence differed substantially between the exposure and outcome datasets.

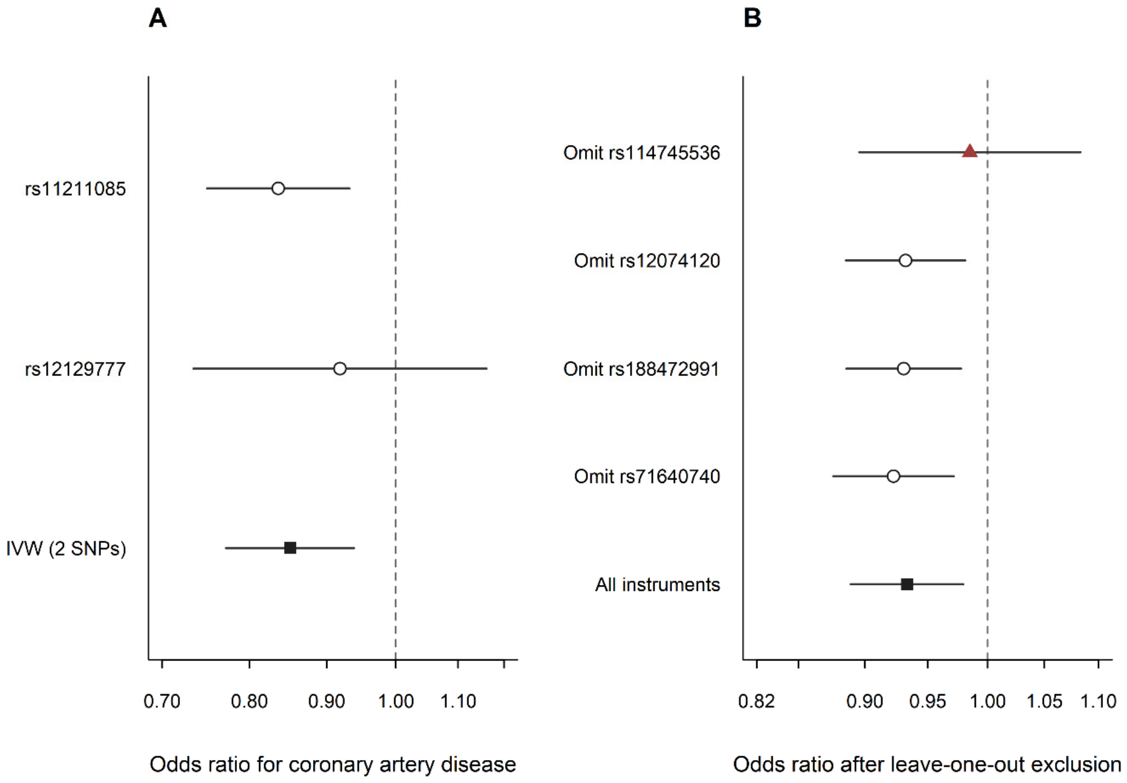

Figure S15. Variant-level influence diagnostics for *SF3A3* and *SNIP1*. (A) Wald-ratio estimates for the two harmonized *SF3A3* instruments together with the primary inverse-variance-weighted estimate. The SNP-specific estimates were directionally concordant, although statistical support was stronger for rs11211085. (B) *SNIP1* leave-one-out analysis. The filled square denotes the estimate using all four instruments, open circles denote estimates obtained after exclusion of each non-influential variant, and the red triangle denotes exclusion of rs114745536. Removal of rs114745536 attenuated the estimate toward the null, supporting classification of the *SNIP1* result as single-variant-sensitive.

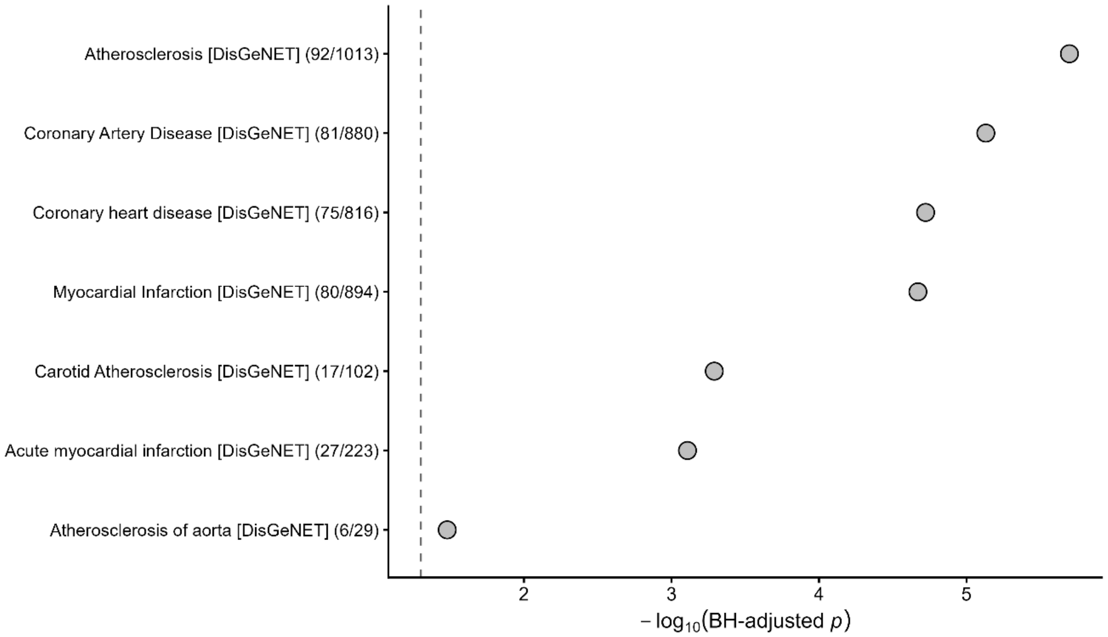

Figure S16. Atherosclerotic and coronary disease enrichment among the 830 locked shared leading-edge genes. One-sided hypergeometric over-representation analysis was performed using the 16,674 HGNC-recognized genes measured in the study (Table S12) as the custom background, rather than the whole genome. The disease-oriented Enrichr libraries queried were DisGeNET, Jensen_DISEASES_Experimental_2025, GWAS_Catalog_2025, ClinVar_2025, and OMIM_Disease. Seven unique FDR-significant terms met the prespecified disease-domain criteria for atherosclerosis, coronary artery disease, myocardial infarction, peripheral arterial disease, or carotid atherosclerosis and are shown. Duplicate library–term combinations were removed before plotting, and no disease term was manually added. Point position represents $-\log_{10}$ of the Benjamini–Hochberg-adjusted p value calculated within each library. Values in parentheses indicate the number of query genes overlapping the term followed by the number of term-associated genes present in the custom background. The dashed vertical line indicates an adjusted p value of 0.05. Sleep-related disease terms, including non-significant results, are reported in Table S54. Because the 830-gene set was analyzed without regard to direction of expression, the enrichment results indicate disease-associated gene-set overlap rather than directional regulation or causality.

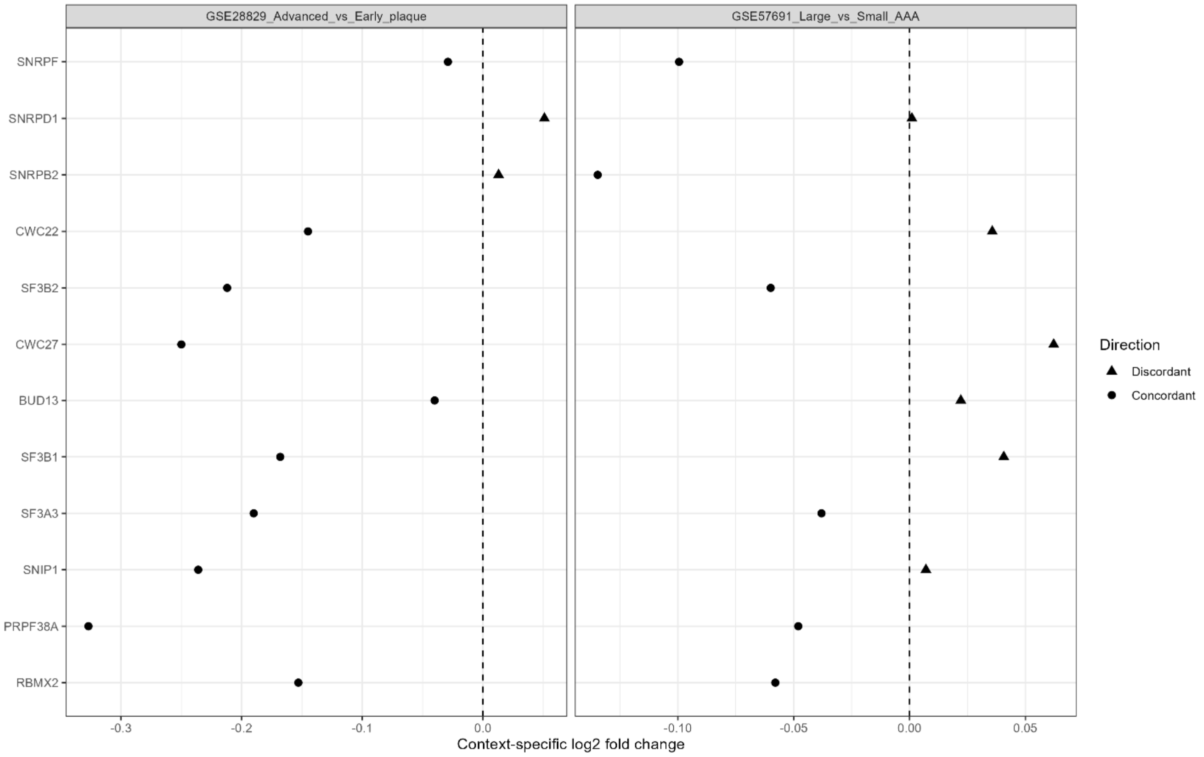

Figure S17. Descriptive cross-dataset behavior of the 12 prespecified MCC-supported candidate genes. Context-specific log_2 fold changes are shown for the 12 prespecified MCC-supported candidates in advanced versus early atherosclerotic plaque (GSE28829) and large versus small abdominal aortic aneurysm (GSE57691). The vertical dashed line denotes no differential expression. Candidate-level results are presented descriptively because the genes arise from a shared spliceosomal network and were not treated as independent inferential units. Directional behavior was interpreted relative to the locked GSE43292 reference effects; the analysis was not designed as external validation or replication.

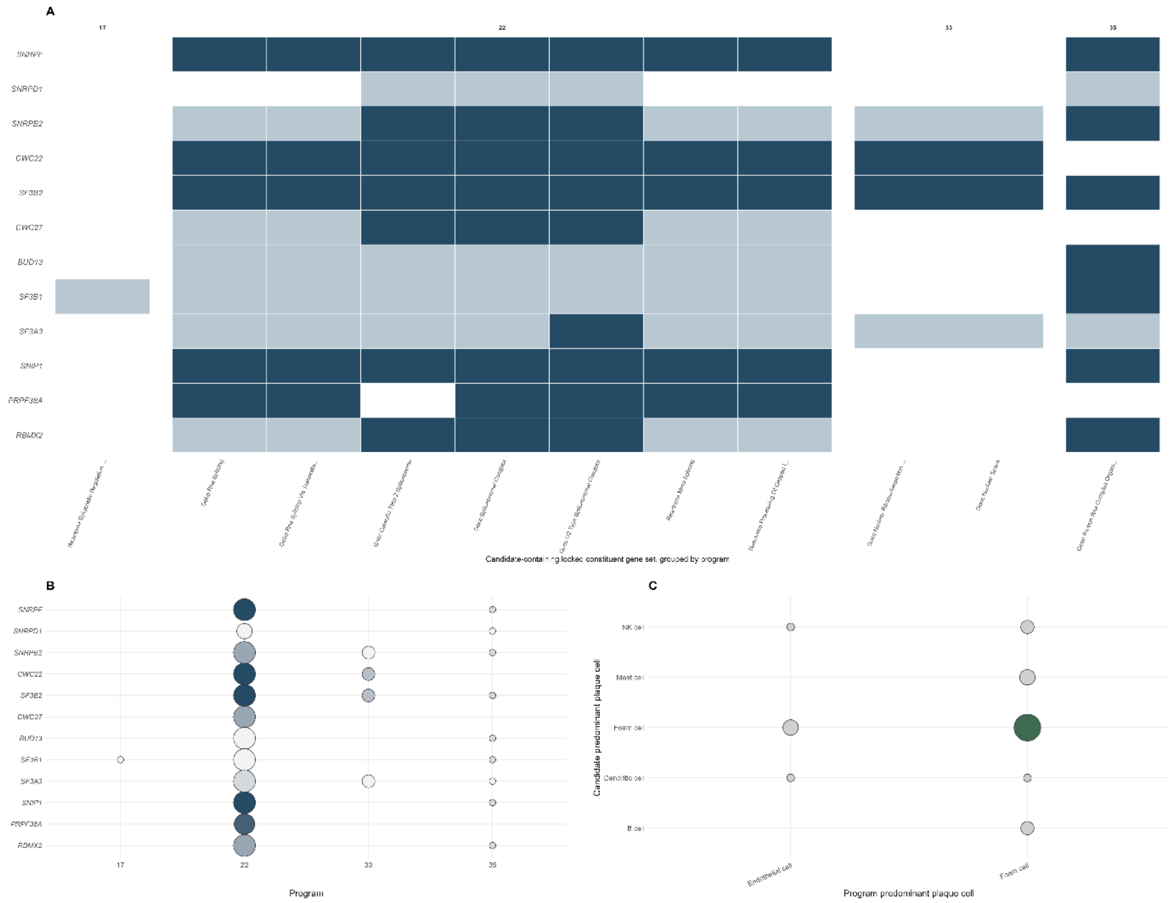

Figure S18. Detailed candidate membership across candidate-containing locked gene sets and the cellular composition of candidate–program links. (A) Matrix showing the 11 locked constituent gene sets containing at least one of the 12 prespecified candidates in a cohort-specific leading edge. The complete mapping universe comprised 71 locked gene sets; the remaining 60 sets contained none of the prespecified candidates. Dark blue cells indicate candidate membership in the same locked gene set in both cohort-specific leading-edge maps, whereas light blue cells indicate membership in one map. Gene sets are grouped by fixed immune–vascular program. (B) Strength and cross-cohort composition of the 26 candidate–program links. Point size represents the total number of directly supporting gene sets, and darker fill represents a larger number of cross-cohort-supported sets. (C) Relationship between candidate- and program-level predominant plaque-cell contexts. Point size represents the number of candidate–program links for each cellular pairing; green indicates concordant predominant cellular context and grey indicates distinct contexts.

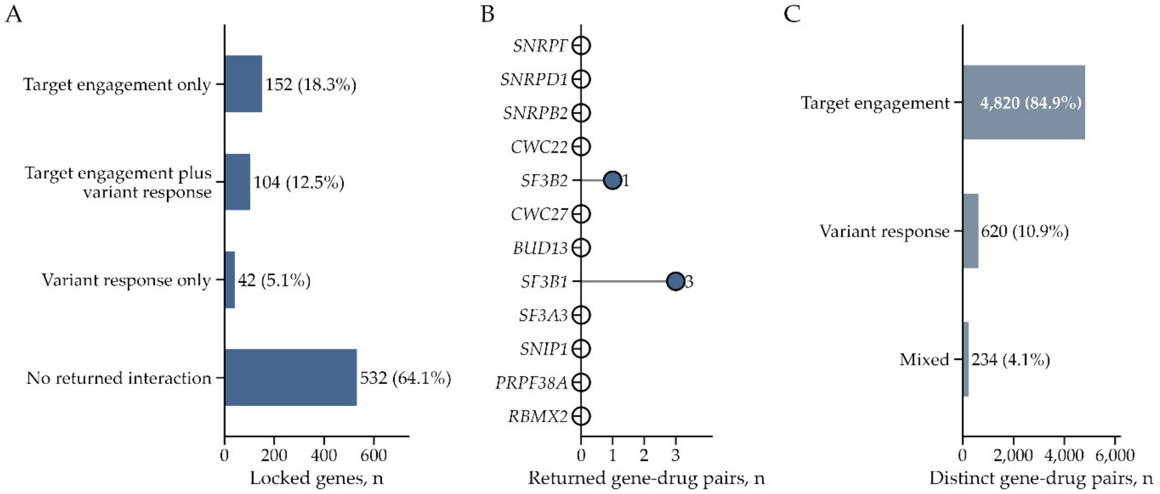

Figure S19. DGIdb-based pharmacological interaction annotation of the locked sleep-loss–atherosclerosis gene set. (A) Gene-level distribution of pharmacological evidence classes across the 830 locked shared leading-edge genes. Mixed gene–drug pairs were counted as contributing to both target-engagement and variant-response evidence at the gene level. (B) Numbers of DGIdb-recorded gene–drug pairs for the 12 prespecified MCC-supported candidates, shown in their fixed order. Only SF3B2 and SF3B1 had returned pairs, with one and three pairs, respectively. (C) Source-class distribution of the 5,674 distinct DGIdb gene–drug pairs. Target-engagement, variant-response, and mixed classes were assigned from the contributing DGIdb sources according to the prespecified source dictionary reported in Table S59. These panels describe database-recorded pharmacological coverage and source composition only; they do not indicate transcriptomic reversal, compound prioritization, therapeutic efficacy, causal target engagement, or clinical actionability.

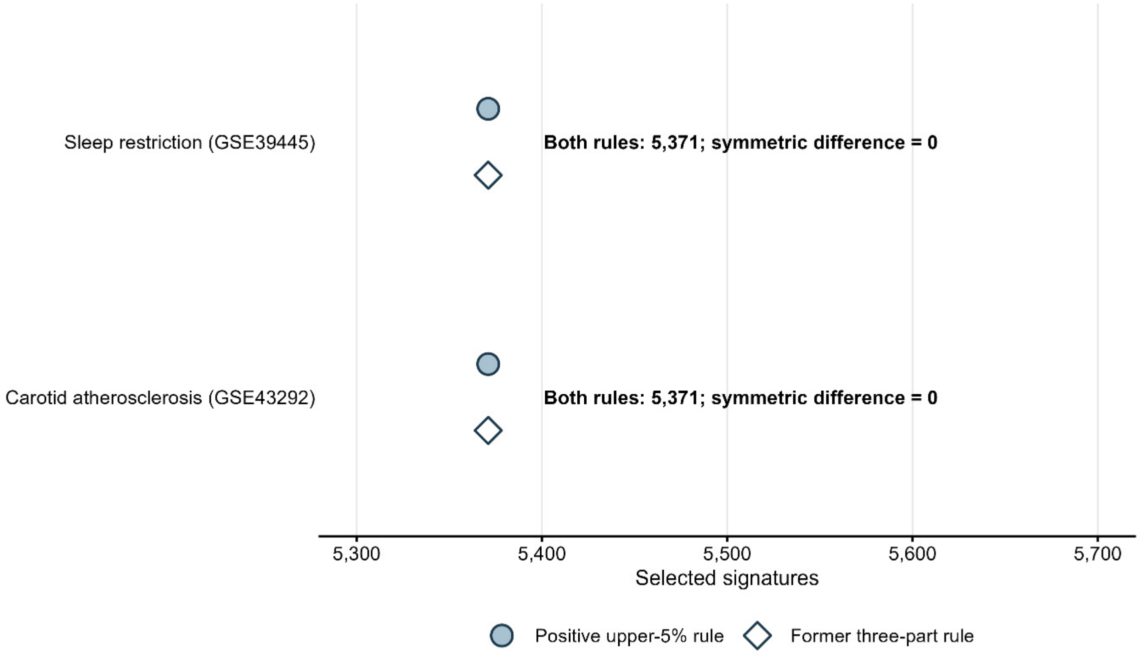

Figure S20. Identity of the positive upper-5% release-relative rule and the former three-part rule. Circles denote the positive upper-5% rule, and diamonds denote the former three-part rule, which additionally required a pooled permutation Benjamini–Hochberg-adjusted q value below 0.05. Both rules selected 5,371 signatures in each primary BING cohort, with a symmetric difference of zero in both cohorts. Thus, every signature selected by the positive upper-5% rule also satisfied the pooled-q condition, and addition of this condition did not alter signature- or perturbagen-level membership. Permutation-derived q values were therefore retained only as calibration annotations and were not used to determine membership.

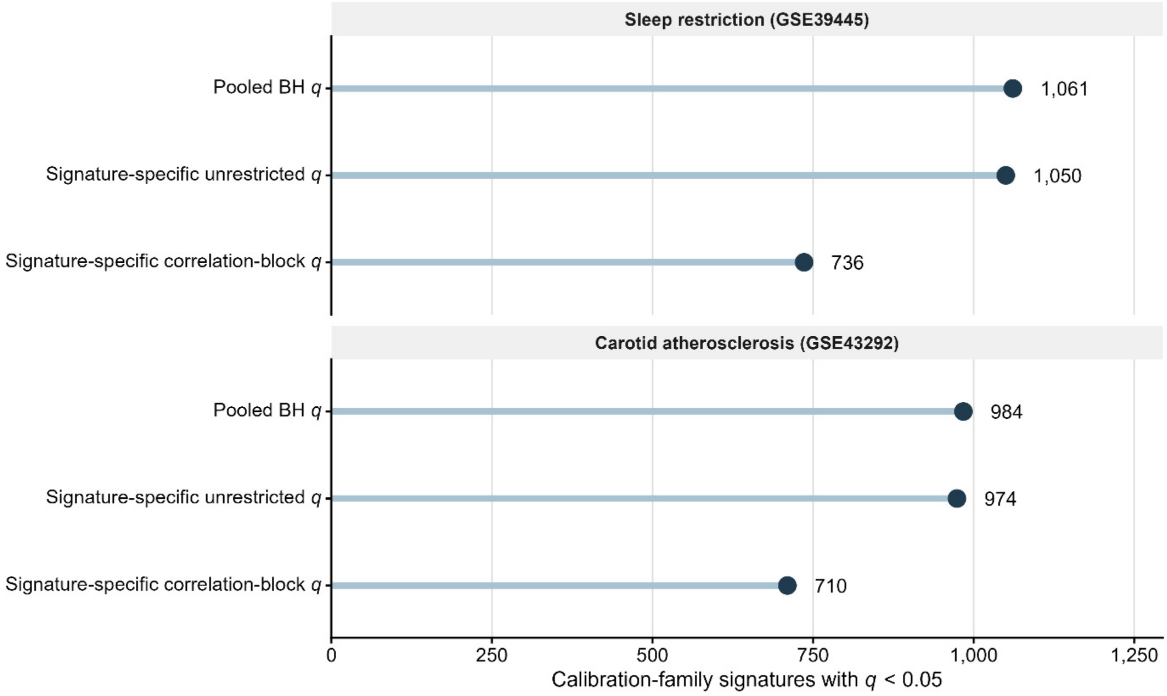

Figure S21. BING calibration under alternative permutation-null specifications. Counts of signatures with permutation-derived q values below 0.05 are shown within the fixed 5,000-signature calibration family for the pooled, signature-specific unrestricted, and signature-specific correlation-block nulls. The corresponding counts were 1,061, 1,050, and 736 in the sleep-restriction cohort and 984, 974, and 710 in the carotid atherosclerosis cohort. All three specifications yielded zero q-value-positive signatures in the locked landmark subset in both cohorts. These results characterize null-model calibration only and did not determine perturbagen membership or establish statistical significance.

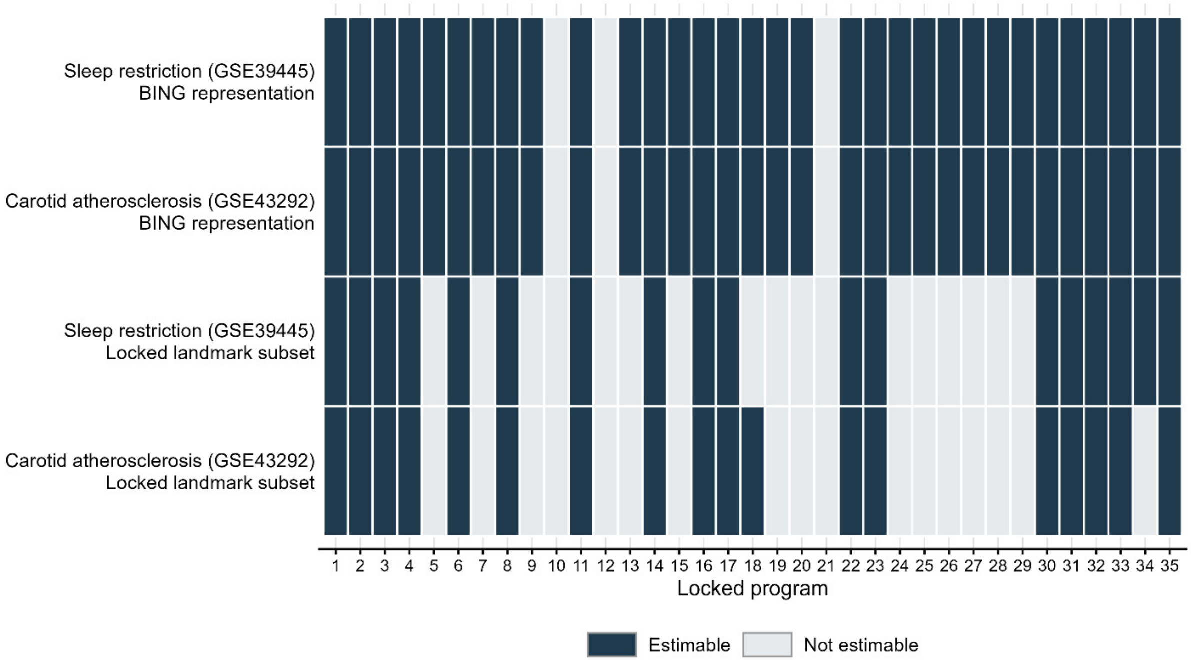

Figure S22. Program estimability across cohorts and LINCS gene representations. Dark tiles indicate estimable program–cohort–representation combinations, whereas light-gray tiles indicate combinations that were not estimable. In the BING representation, 32 of the 35 locked programs were estimable in both primary cohorts; Programs 10, 12, and 21 were not estimable in either cohort and therefore could not contribute cross-cohort program context. Landmark-subset estimability is shown as a directional sensitivity layer. Program estimability provided descriptive mechanistic context and was not an inferential selection gate.

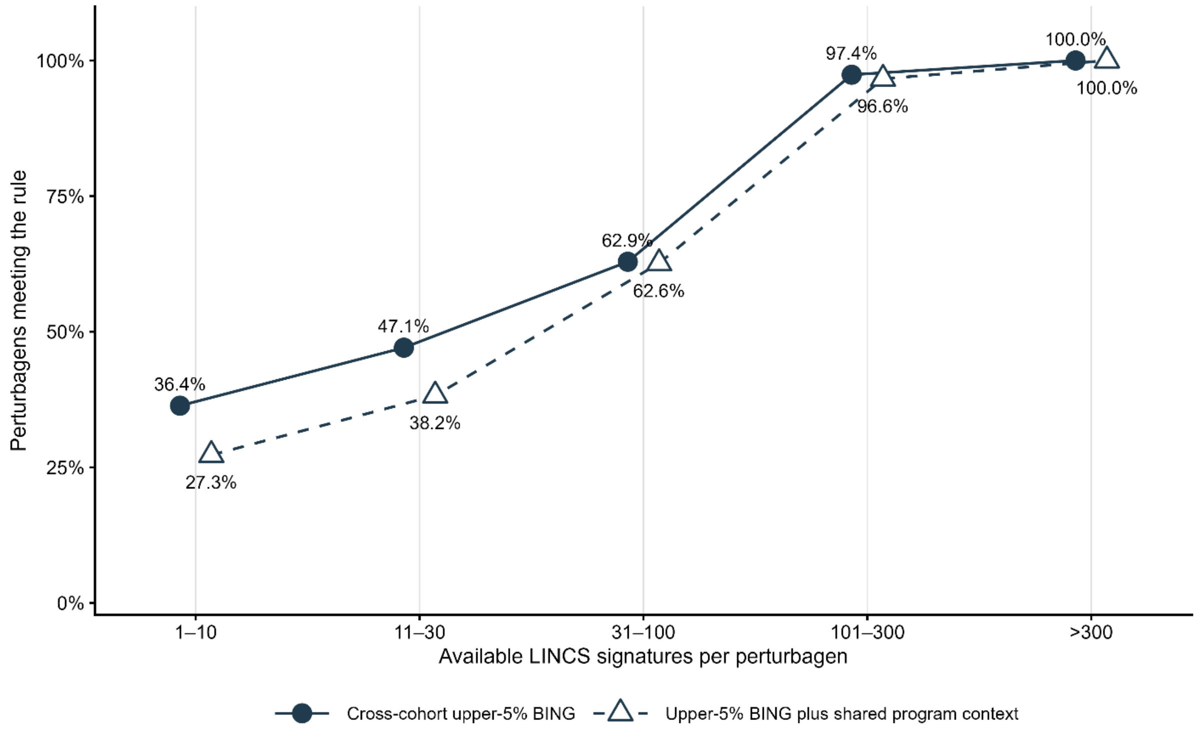

Figure S23. Dependence of perturbagen-level membership on LINCS signature coverage. Filled circles connected by a solid line denote cross-cohort upper-5% BING membership, whereas open triangles connected by a dashed line denote the subset additionally having shared locked program context. Point labels give the observed proportions within each signature-count group. Across the 1–10, 11–30, 31–100, 101–300, and >300 groups, cross-cohort upper-5% BING membership was 36.4%, 47.1%, 62.9%, 97.4%, and 100%, respectively; the corresponding proportions with shared program context were 27.3%, 38.2%, 62.6%, 96.6%, and 100%. Lines are visual guides rather than fitted models. Coverage dependence was retained as a limitation; no coverage-adjusted ranking, weighting procedure, corrective threshold, or post hoc membership modification was applied.

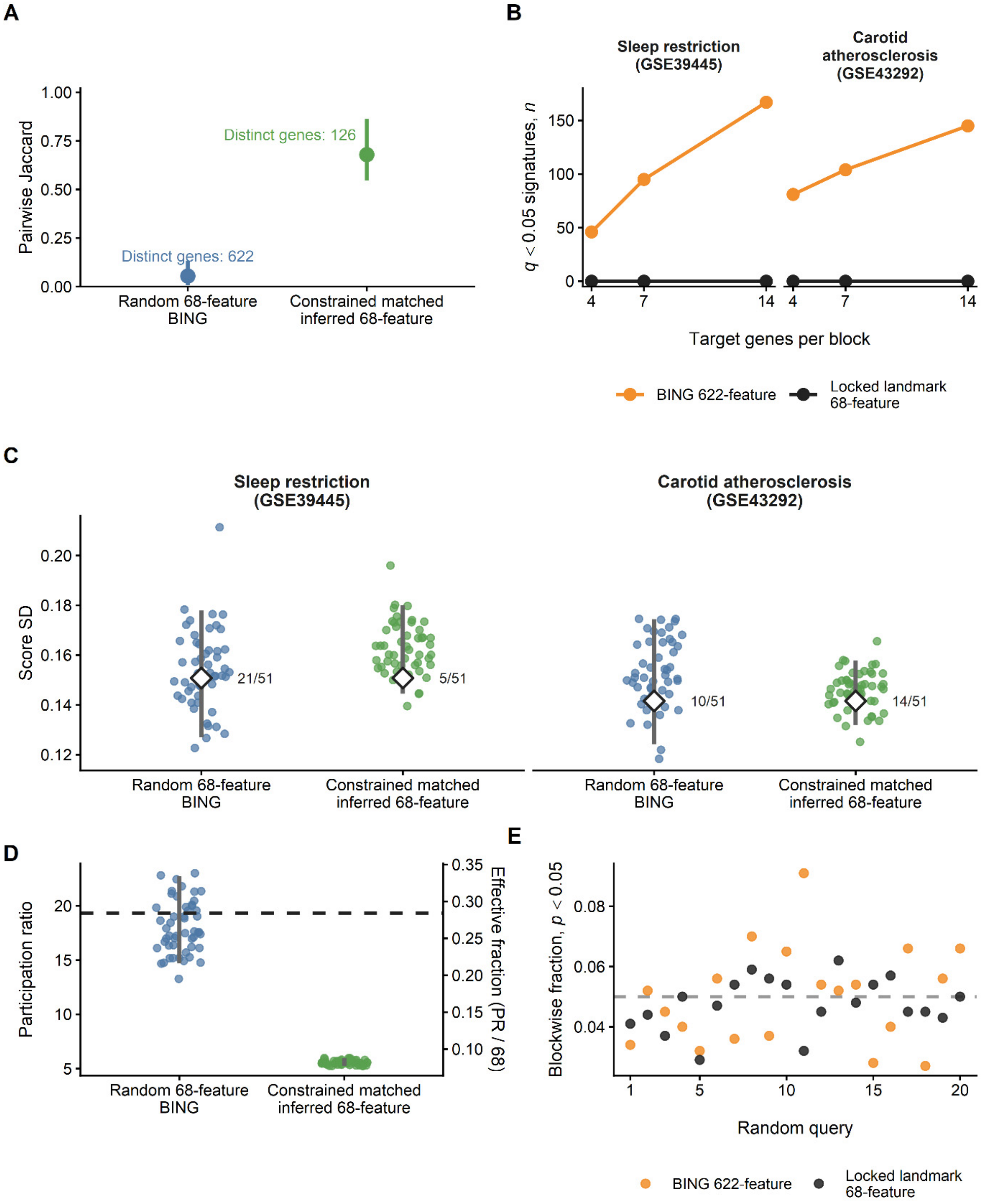

Figure S24. Fixed-size calibration and sensitivity analysis of LINCS gene-space behavior. (A) Structural characteristics of the fixed-size comparator arms. Points denote median pairwise Jaccard overlap and vertical ranges the corresponding minimum-to-maximum values across 50 random 68-feature BING subsets and 50 constrained matched 68-feature inferred subsets; annotations indicate the total number of distinct genes represented within each comparator arm. (B) Sensitivity of blockwise $q < 0.05$ signature

counts to correlation-block granularity. Counts are shown at target block sizes of 4, 7, and 14 genes for the 622-feature BING representation and the locked 68-feature landmark subset, separately for the sleep-restriction and carotid-atherosclerosis cohorts. (C) Score-dispersion calibration at a fixed nominal size of 68 features. All 50 comparator-set score standard deviations are shown for each comparator arm and cohort. Vertical bars denote empirical 2.5th–97.5th percentile ranges, diamonds denote the locked landmark value, and annotations give its rank within the combined set of 50 comparator values and the landmark value (n = 51). These percentile ranges are descriptive comparator ranges and are not confidence intervals. (D) Participation-ratio effective dimensionality of the two 68-feature comparator arms. All 50 values are shown without cohort stratification because participation ratio is a structural property of the gene space estimated from the common disjoint reference family. The dashed horizontal line denotes the locked landmark participation ratio; the secondary axis expresses the same quantity as effective fraction (participation ratio/68) and is not an independent measure. (E) Random-query calibration of the BING and locked landmark gene spaces. Points denote the blockwise fraction of signatures with $p < 0.05$ across 20 random queries in each gene space; the dashed horizontal line denotes the nominal 0.05 reference level. The constrained matched comparator arm exhibited substantial between-set overlap and lower effective dimensionality than the locked landmark subset and was therefore treated as a constrained comparator rather than as an isolated test of gene composition.

Supplementary Tables

Table S1. Complete gene-level differential-expression results for GSE39445 comparing sleep restriction with sleep extension (19,541 gene-symbol features).

Table S2. Genes meeting both prespecified differential-expression criteria in GSE39445 (1,352 genes; 645 upregulated and 707 downregulated during sleep restriction relative to sleep extension).

Table S3. Complete gene-level differential-expression results for GSE43292 comparing carotid plaque with paired macroscopically intact arterial tissue (21,147 gene-symbol features).

Table S4. Genes meeting both prespecified differential-expression criteria in GSE43292 (3,719 genes; 1,946 upregulated and 1,773 downregulated in plaque relative to paired intact arterial tissue).

Table S5. Complete gene-level differential-expression results for the GSE28829 plaque-progression analysis comparing advanced with early atherosclerotic plaque (24,442 gene-symbol features).

Table S6. Genes meeting both prespecified differential-expression criteria in GSE28829 (2,218 genes; 1,202 upregulated and 1,016 downregulated in advanced relative to early plaque).

Table S7. Summary crosswalk between gene-symbol features and distinct selected-probe measurements in GSE28829 (24,442 gene-symbol features corresponding to 22,807 distinct selected-probe measurements; 2,218 significant gene-symbol features corresponding to 2,091 distinct selected-probe measurements).

Table S8. Thirty genes with the largest positive and thirty genes with the largest negative $\log_2$ fold changes among genes meeting both prespecified differential-expression criteria in the GSE28829 plaque-progression analysis.

Table S9. Summary of the GSE57691 gene-level aneurysm-severity analysis comparing large with small abdominal aortic aneurysms (25,235 gene-symbol features; no gene met the Benjamini–Hochberg false-discovery-rate threshold).

Table S10. Thirty genes with the smallest nominal p values in the GSE57691 aneurysm-severity analysis; none met the Benjamini–Hochberg false-discovery-rate threshold.

Table S11. Computational audit of the negative false-discovery-rate result in the GSE57691 aneurysm-severity analysis, including alternative probe-to-gene summarization rules, variance specifications, and permutation-based assessments of the nominal gene count and moderated-t-statistic dispersion.

Table S12. Shared measured-gene universe and differential-expression overlap between GSE39445 and GSE43292 (16,869 shared measured genes; 274 genes meeting the prespecified criteria in both cohorts), including the hypergeometric overlap assessment.

Table S13. Effect directions and concordance classification for the 274 genes meeting the prespecified differential-expression criteria in both discovery cohorts (134 directionally concordant genes).

Table S14. Complete transcriptome-wide gene set enrichment analysis results for the two discovery cohorts, including normalized enrichment scores for the 8,284 gene sets evaluable in both datasets.

Table S15. Ninety-one gene sets significant in both discovery cohorts, classified by directional concordance (71 concordant and 20 discordant; 45 positively enriched in both cohorts and 26 negatively enriched in both).

Table S16. Program membership under the prespecified reference clustering specification based on the overlap coefficient, average linkage, and a similarity threshold of 0.50, yielding 50 redundancy-collapsed programs.

Table S17. Thirty-five directionally concordant programs, including program direction, program size, median normalized enrichment scores in the sleep-loss and atherosclerosis discovery cohorts, and constituent gene sets.

Table S18. Sensitivity analysis across the prespecified 24-cell clustering grid comprising alternative similarity metrics, linkage methods, and similarity thresholds.

Table S19. Program-level directional-generalization results in the primary GSE56931 acute total sleep-deprivation analysis comprising 209 samples (32 of 35 programs directionally concordant).

Table S20. Program-level results from the GSE56931 matched-clock sensitivity analysis restricted to the first 24 hours of sleep deprivation (28 of 35 programs directionally concordant).

Table S21. Observed areas under the receiver operating characteristic curve and participant-cluster-bootstrap confidence intervals for the nine prespecified feature–model combinations in the GSE39445 within-cohort participant-grouped separability analysis.

Table S22. Permutation-analysis summary for the participant-grouped separability models, including observed and empirical-null results, fold assignments, and random-seed specifications.

Table S23. Hallmark gene set variation analysis results for GSE43292 (50 Hallmark pathways; 31 Benjamini–Hochberg false-discovery-rate-significant, of which 30 showed higher inferred activity in plaque and one showed higher inferred activity in paired intact arterial tissue).

Table S24. Co-expression module sizes for the weighted gene co-expression network constructed from 14,655 genes in the GSE39445 whole-blood discovery cohort using a signed network and a soft-thresholding power of 7.

Table S25. Module–eigengene associations with the sleep-restriction contrast for the sixteen non-grey co-expression modules in the primary covariate-adjusted linear mixed-effects analysis. Positive coefficients indicate higher eigengene expression during sleep restriction.

Table S26. Comparison of primary covariate-adjusted and subject-only sensitivity estimates for the four co-expression modules meeting the Benjamini–Hochberg false-discovery-rate threshold in the primary module–trait analysis.

Table S27. Complete module-membership correlation matrix for all 14,655 network genes across the sixteen non-grey module eigengenes, with descriptive membership p values and the grey eigengene-like summary retained for completeness. The grey category represents genes not assigned to a non-grey co-expression module and was excluded from biological interpretation.

Table S28. Complete intramodular gene rankings for the four sleep-associated co-expression modules, ordered by signed own-module membership (kME) within each module.

Table S29. Hub-gene summary for the four sleep-associated co-expression modules, listing the five genes with the highest signed own-module membership values in each module.

Table S30. Complete GSE39445 MCP-counter results**.** Primary covariate-adjusted mixed-effects estimates, standard errors, confidence intervals, nominal *p* values, FDR values, and reduced-model sensitivity results for all ten evaluated populations.

Table S31. Complete GSE39445 prespecified marker-score results. Primary covariate-adjusted mixed-effects estimates, standard errors, confidence intervals, nominal *p* values, FDR values, and reduced-model sensitivity results for all ten evaluated populations.

Table S32. GSE39445 cross-method concordance. Population-level comparison of MCP-counter and prespecified marker-score estimates, within-cohort coefficient-direction concordance, and sample-level Spearman correlations.

Table S33. Complete GSE43292 MCP-counter results. Patient-paired primary-model estimates, standard errors, confidence intervals, nominal *p* values, FDR values, and corresponding unpaired sensitivity-model results for all ten evaluated populations.

Table S34. Complete GSE43292 prespecified marker-score results**.** Patient-paired primary-model estimates, standard errors, confidence intervals, nominal *p* values, FDR values, and corresponding unpaired sensitivity-model results for all ten evaluated populations.

Table S35. GSE43292 cross-method concordance. Population-level comparison of MCP-counter and prespecified marker-score estimates, coefficient-direction concordance, joint FDR status, and sample-level Spearman correlations for all ten evaluated populations.

Table S36. Cross-cohort integration and marker definitions. Complete two-cohort directional-integration table for all ten populations, MCP-counter cross-cohort concordance, marker-score cross-cohort concordance, four-layer directional-concordance status, prespecified marker-gene membership, and dataset-specific marker measurement coverage.

Table S37. Complete primary TF-target regulatory-context over-representation results across the three prespecified resources. Resource-specific mapped backgrounds and query sizes are reported explicitly.

Table S38. FDR-significant primary TF-target regulatory-context findings. CollecTRI results are reported as curated regulons, whereas GTRD results are reported as regulatory target signatures.

Table S39. Sensitivity of the seven primary FDR-significant findings across three prespecified input-gene definitions. No result was interpreted as direct TF binding, TF activity, causal regulation, or independent cross-resource validation.

Table S40. Complete locked 830-gene shared leading-edge input used for STRING protein–protein interaction analysis. The gene set was defined as the intersection between the cohort-specific unions of core-enrichment genes across the 71 directionally concordant gene sets. The table reports the complete submitted query-gene list before STRING identifier mapping and is not partitioned according to enrichment direction.

Table S41. Complete STRING functional enrichment results for the 828 mapped protein nodes. The table contains all 2,762 FDR-significant terms returned by STRING version 12.0 across 14 annotation sources in the downloaded enrichment output, excluding PubMed. The locked input comprised 830 unique human gene symbols; *GGT3P* and *HSP90AB3P* were not represented as protein nodes. Term identifiers, observed and background gene counts, enrichment strength, signal, false discovery rate, and matching protein identifiers and gene labels are reproduced directly from the STRING export without term selection or recalculation.

Table S42. Complete node-level network metrics and maximal clique centrality scores for the STRING-derived PPI network. Degree and clustering coefficient were calculated with Analyze Network in Cytoscape 3.10.4 for all 828 mapped protein nodes. Betweenness and closeness centrality were calculated within the 626-node largest connected component, ensuring that shortest-path-based values were derived within a common connected topology; these fields are therefore reported as not applicable for the 202 proteins outside that component. Maximal Clique Centrality scores were obtained with cytoHubba for the complete 828-node network. The network was treated as undirected. Five cytoHubba values affected by floating-point representation were rounded to the nearest integer, consistent with the integer-valued definition of MCC; equal corrected scores share the same competition rank. Minimal score differences within the highest-scoring group were not interpreted as a meaningful within-group hierarchy. Network position does not establish causal importance, disease specificity, direct regulation, or therapeutic efficacy.

Table S43. Cluster-level marker-primary annotation with independent SingleR concordance. The table reports the complete annotation record for the 19 Leiden clusters, including the original cluster identifier, cell count, marker-primary assignment, second-ranked marker group, marker-score margin, Human Primary Cell Atlas and Blueprint/ENCODE SingleR classifications, corresponding broad-label mappings, reference-specific concordance indicators, independent-support count, and annotation status. Marker-primary classification provided the definitive cellular assignment, whereas SingleR served as an orthogonal reference-based assessment. Seven clusters were concordant with both reference collections, three were concordant with one reference collection, and nine had no concordant broad reference label.

Table S44. Cluster-level marker-primary cell-type assignment and abundance. The table reports the marker-primary cell-type assignment and cell count for each of the 19 Leiden clusters, together accounting for all 49,188 retained cells. Because cellular identities were assigned at cluster level and transferred to the constituent cells, each cluster carries one marker-primary category and therefore has a within-cluster percentage of 100%. This percentage documents the deterministic cluster-to-cell-type mapping and should not be interpreted as a probabilistic estimate of cluster purity.

Table S45. Cell-type localization of the 830 locked Table S40 genes in GSE253903. Average log-normalized expression and detection percentage are reported across the 12 marker-primary cell types. For each measured gene, the table provides the predominant cell type, maximum average expression, corresponding detection percentage, maximum cell-type z-score, and complete cell-type-specific expression and detection values. Of the 830 genes, 822 were represented in the final expression matrix. CST11, DEFB114, GGT3P, HSP90AB3P, RNASE8, SLC2A2, SPAG11B, and UTP14C were not measured and therefore have blank localization fields. Gene-wise z-scores describe relative localization across cell types and are not differential-expression effect sizes.

Table S46. Cell-type localization of the 12 locked MCC-supported candidates. The table reports the prespecified MCC display order and complete cell-type localization metrics for SNRPF, SNRPD1, SNRPB2, CWC22, SF3B2, CWC27, BUD13, SF3B1, SF3A3, SNIP1, PRPF38A, and RBMX2. All 12 candidates were represented in the final GSE253903 expression matrix. For each candidate, the table provides the predominant marker-primary cell type, maximum average log-normalized expression, corresponding detection percentage, maximum cell-type z-score, and complete average-expression and detection values

across the 12 cell types. Candidate identity and display order follow the preceding network-topology analysis.

Table S47. Cell-type localization of the 35 fixed directionally concordant programs. The table retains the program number, size, median normalized enrichment scores in the sleep-loss and atherosclerosis discovery cohorts, direction, constituent gene sets, total number of unique MSigDB genes, and number of genes measured in GSE253903. For each program, the predominant marker-primary cell type, maximum localization score, maximum cell-type z-score, and complete cell-type-specific localization scores and z-scores are reported. The 35 programs comprised 22 concordant-up and 13 concordant-down programs. Measured-gene coverage ranged from 61.3% to 100.0%, with a median of 90.8%. Program definitions, sizes, directions, and constituent gene sets were inherited from the prespecified bulk-transcriptomic program architecture and were not redefined using GSE253903. The reported scores describe cellular localization and do not constitute condition-level differential-expression evidence or direct pharmacological validation.

Table S48. Prespecified human genetic analysis rules, locked candidate-gene exposure datasets, hg19 gene coordinates, corrected coronary artery disease metadata, and final candidate-level instrument availability. The table documents the complete 12-candidate analysis universe, exact-cis and instrument-selection criteria, candidate-specific exposure identifiers and observed sample-size ranges, coordinate mapping, the derivation of 99,121 controls from the total sample size and case count, and the final number of exposure, exact-cis, clumped, strong, outcome-matched, and harmonized instruments retained for each candidate. Candidates without an available genome-wide-significant exposure instrument are retained as non-estimable results.

Table S49. Complete exact-cis exposure-instrument audit, linkage-disequilibrium-clumped instruments, harmonized coronary artery disease associations, and instrument disposition. Panel A contains all 5,219 genome-wide-significant exposure associations satisfying the exact hg19 cis definition and $F > 10$. Panel B reports the 19 independent instruments retained after European-reference linkage-disequilibrium clumping. Panel C reports the 18 exact-SNP coronary artery disease associations retained after conservative allele harmonization. Panel D documents the non-retained SF3A3 instrument rs28525112, whose C/G allele pair was palindromic and whose exposure effect-allele frequency was close to the ambiguity threshold. No post hoc substitute instrument was introduced.

Table S50. Primary and sensitivity Mendelian-randomization results for candidate-gene expression and coronary artery disease. The table reports the primary Wald-ratio or inverse-variance-weighted estimate for each estimable candidate, all prespecified alternative estimators, Cochran Q heterogeneity tests, MR-Egger intercept tests, single-SNP Wald estimates, leave-one-out analyses, sensitivity analyses excluding palindromic SNPs, and Steiger directionality analyses across three assumed coronary artery disease prevalences and both total and effective outcome sample-size definitions. Separate panels summarize the directionally concordant two-SNP SF3A3 decomposition and the single-variant-sensitive SNIP1 classification. All values are reproduced from the locked audit without recalculation.

Table S51. Regional colocalization status, primary posterior results, and p_{12}-prior sensitivity for SF3A3, SNIP1, and SNRPF. Panel A reports regional-query completion and the numbers of complete shared SNPs.

Panel B reports coloc.abf posterior probabilities under the primary $p_{12} = 1 \times 10^{-5}$ prior. Panel C reports sensitivity analyses at $p_{12} = 1 \times 10^{-6}$, 1×10^{-5}, and 5×10^{-5}. SF3A3 showed high posterior support for a shared signal under the primary prior, but the magnitude of PP.H4 was sensitive to the prior specification. SNIP1 favored distinct signals under the primary prior, and SNRPF remained inconclusive because its absolute PP.H4 was low.

Table S52. Shared regional eQTL–coronary-artery-disease summary statistics and SNP-level posterior probabilities used in the colocalization analyses. Panel A contains 9,542 complete shared regional SNP records across SF3A3, SNIP1, and SNRPF. The source-reported outcome sample-size field is preserved for traceability, whereas the colocalization calculations used the corrected coronary artery disease metadata (N = 141,217; 42,096 cases; 99,121 controls), repeated in dedicated columns. Panel B reports the 50 SNPs with the highest SNP-level PP.H4 for each candidate region (150 rows in total). The highest SNP-level posterior probability for SF3A3 was assigned to rs28428561 (PP.H4 = 0.987).

Table S53. Complete disease and phenotype over-representation results for the 830 locked shared leading-edge genes. One-sided hypergeometric over-representation analysis was performed using 16,674 unique HGNC-recognized measured genes as the custom background. Table S12 contained 16,869 locked valid gene-symbol entries before deduplication; duplicated symbols were counted once when constructing the enrichment universe. Benjamini–Hochberg adjustment was applied separately within each library across all eligible tested terms with a minimum background term size of five genes. The table reports the query overlap, background overlap, enrichment ratio, sample odds ratio, raw p value, within-library BH-adjusted p value, FDR significance status, and contributing genes for all 12,968 eligible terms tested across DisGeNET, Jensen_DISEASES_Experimental_2025, GWAS_Catalog_2025, ClinVar_2025, and OMIM_Disease. The 830-gene set was analyzed without regard to expression direction. An em dash indicates that the tested term had zero contributing query genes.

Table S54. Prespecified cardiovascular and sleep-related disease enrichment results for the 830 locked shared leading-edge genes. This table reports eligible tested terms whose clinical meaning matched the prespecified atherosclerosis, coronary artery disease, myocardial infarction, peripheral arterial disease, carotid atherosclerosis, insomnia, sleep deprivation or sleep loss, sleep duration or short sleep, and circadian rhythm or sleep disorder domains. Substring-only false-positive matches and exact cross-domain duplicates were excluded, whereas all retained terms are shown irrespective of statistical significance. No disease term was manually added. Statistical values are unchanged from the complete analysis reported in Table S53 and include the query overlap, background overlap, enrichment ratio, sample odds ratio, raw p value, within-library BH-adjusted p value, FDR significance status, and contributing genes. An em dash indicates either zero contributing query genes or that no eligible tested term matched the prespecified domain.

Table S55. Disease-domain annotation overlap of the 12 locked MCC-supported candidate genes. Panel A summarizes, for each candidate gene, whether it occurred among the contributing genes of any clinically curated term reported in Table S54. Candidate-domain relationships are classified as FDR-significant when the candidate occurred in at least one matched term with a within-library BH-adjusted p value below 0.05, non-significant when the candidate occurred only in matched terms with BH-adjusted p values of 0.05 or greater, and no overlap when the candidate was absent from all matched terms. Panel B lists every observed

candidate–term overlap together with the corresponding disease domain, source library, matched term, query overlap, background overlap, raw p value, BH-adjusted p value, and FDR significance status. These descriptive annotations do not constitute independent validation, candidate-specific enrichment analysis, causal inference, Mendelian-randomization reanalysis, or colocalization reanalysis.

Table S56. Cross-dataset directional and pathway concordance of the locked sleep-loss-associated vascular transcriptional architecture. Table S56 presents the prespecified cross-dataset analysis in advanced versus early atherosclerotic plaque (GSE28829) and large versus small abdominal aortic aneurysm (GSE57691). Table S56A summarizes program- and gene-level concordance; Table S56B reports directional concordance across the 35 fixed transcriptional programs; Table S56C provides the complete GSEA results for the 71 locked constituent gene sets; Table S56D reports gene-level directional concordance across the 830 locked shared leading-edge genes; Table S56E presents the descriptive cross-dataset behavior of the 12 prespecified MCC-supported candidates; and Table S56F provides definitions, interpretation notes, and abbreviations. Program- and gene-level directions were evaluated relative to the locked GSE43292 reference architecture. These analyses characterize disease-context preservation or divergence rather than external validation or replication.

Table S57. Multilayer characterization of the 12 prespecified MCC-supported candidates within the sleep-loss–atherosclerosis framework. Table S57 presents candidate-level profiles integrating network degree, membership in retained sleep-associated WGCNA modules, regulatory-context overlap, predominant expression across human atherosclerotic plaque cell populations, primary exact-cis Mendelian randomization, regional colocalization, and directional behavior during plaque progression.

Table S58. Candidate–program–plaque-cell integrative mapping of the 12 prespecified MCC-supported candidates within the sleep-loss–atherosclerosis framework. The table reports the 26 direct candidate–program links defined by candidate membership in the leading-edge genes of at least one locked constituent gene set, together with supporting gene-set number and identity, cross-cohort leading-edge context, candidate and program plaque-cell localization, cellular concordance, and prespecified genetic annotations.

Table S59. DGIdb-based pharmacological interaction annotation of the 830 locked shared leading-edge genes and the nested set of 12 prespecified MCC-supported candidates. DGIdb application v5.0.11 was queried through the live GraphQL endpoint on 2 August 2026. At the gene level, 152 genes had target-engagement evidence only, 104 had both target-engagement and variant-response evidence, 42 had variant-response evidence only, and 532 had no returned DGIdb interaction. Mixed pairs contributed evidence to both source classes at the gene level. Returned gene–drug pairs were classified according to contributing source type as target-engagement, variant-response, or mixed evidence, and all records were retained without compound ranking or filtering. Among the 12 prespecified candidates, no named target-engagement compound was identified: the three SF3B1 pairs were classified as variant-response records, whereas the single SF3B2 target-engagement record did not provide a compound name. None of the four candidate-associated gene–drug pairs contained DGIdb directionality or interaction-type annotations. Source-based classification may underrepresent direct target relationships when a pharmacologically active compound is contributed solely by a variant-response source; H3B-8800 is an example in the present dataset. The table reports locked-gene coverage, candidate-level coverage, distinct gene–drug pairs, source-

class assignments, DGIdb-provided interaction-type and directionality annotations, contributing sources, publication identifiers, interaction scores, and approval annotations where available. The candidate subset was retained in its fixed prespecified order. No gene, candidate, or compound reselection, cross-layer ranking, composite scoring, perturbation-signature reversal, or inference of therapeutic efficacy was performed. Absence of a returned DGIdb interaction should not be interpreted as absence of pharmacological tractability.

Table S60. Descriptive, release-relative LINCS perturbation-signature prioritization for the locked shared sleep-loss–atherosclerosis gene set. Panels S60A–S60J summarize the prespecified evidence flow, identity of the upper-5% and former three-part rules, permutation-calibration diagnostics, participation-ratio effective dimensionality, program estimability, perturbagen coverage, cross-cohort dependence, analytical boundaries, and complete perturbagen-level results. Of 1,796 evaluated perturbagens, 1,216 met the cross-cohort upper-5% BING membership criterion, 1,206 additionally showed shared program context, and 962 additionally showed bilateral directional landmark context.

Table S61. Extended fixed-size calibration of the 68-feature LINCS gene space against random and constrained matched comparator subsets. The locked 68-feature landmark representation was evaluated against 50 random 68-feature subsets of the best inferred genes (BING) space and 50 constrained matched 68-feature inferred subsets to characterize its behavior independently of the gross difference in nominal feature count between the 622-feature BING and 68-feature landmark representations. Random subsets sampled the BING feature space broadly, whereas the constrained matched subsets exhibited substantial between-set overlap and markedly reduced effective dimensionality, precluding their interpretation as an isolated test of gene-composition effects (S61A). In both cohorts, the standard deviation of landmark reversal scores lay within the empirical 2.5th–97.5th percentile ranges of both 68-feature comparator distributions (S61B). The landmark participation ratio likewise lay within the random-BING distribution but outside the constrained matched distribution, consistent with the pronounced covariance structure of the latter. Blockwise and unrestricted $q < 0.05$ counts were uniformly zero across all three 68-feature arms and were therefore non-discriminating; this floor effect was not interpreted as evidence of equivalence (S61C). The absence of blockwise q-positive landmark signatures persisted across target block sizes of 4, 7, and 14 genes, whereas the corresponding BING counts varied with block granularity (S61D). Random-query analyses provided an additional calibration reference for the two gene spaces (S61E). Participation ratio and effective fraction were derived from the common disjoint reference family and represent structural properties of the gene space rather than cohort-specific quantities. Taken together, the fixed-size analyses indicate that the score-dispersion behavior of the locked landmark subset was not exceptional among the evaluated 68-feature comparators, while the dependency structure of the constrained matched arm prevents clean attribution of the BING–landmark contrast to feature composition. These analyses were calibration and sensitivity procedures only and did not redefine perturbagen membership, alter compound prioritization, or provide evidence of therapeutic efficacy or clinical actionability.

Table S62. Sensitivity of the GSE39445 discovery analysis to the representative-probe selection rule. Gene-level, gene-set-level, and program-level results obtained using the submitted rule (smallest adjusted *p* value, with the largest absolute $\log_2$ fold change resolving exact ties) and a contrast-independent maximum-IQR rule applied to the same annotated probe-level expression matrix and samples. The differential-expression design and covariates were unchanged, and both specifications used the independently reproduced full-probe consensus within-participant correlation. The GSE43292 arm, gene-

set search space, and prespecified 50-program membership were held fixed; no re-clustering was performed.
